# Compliance and Toxicity of Total Neoadjuvant Therapy in Locally Advanced Rectal Cancer: A Systematic Review and Network Meta-analysis

**DOI:** 10.1245/s10434-025-17421-7

**Published:** 2025-05-05

**Authors:** Warren Seow, Ishraq Murshed, Zachary Bunjo, Sergei Bedrikovetski, Jennifer Stone, Tarik Sammour

**Affiliations:** 1 Department of Surgical Specialties, Adelaide Medical School, Faculty of Health and Medical Sciences, University of Adelaide, Adelaide, SA Australia; 2 JBI, Faculty of Health and Medical Sciences, University of Adelaide, Adelaide, SA Australia; 3 Colorectal Unit, Department of Surgery, Royal Adelaide Hospital, Adelaide, SA Australia

**Keywords:** Total neoadjuvant therapy, Rectal cancer, Chemoradiation, Chemotherapy, Radiotherapy

## Abstract

**Purpose:**

The individual chemotherapy- and radiotherapy-related toxicities between induction (iTNT) and consolidation total neoadjuvant therapy (cTNT) remain unclear. This network meta-analysis (NMA) comparing iTNT, cTNT, and traditional neoadjuvant chemoradiation (nCRT) evaluated the comparative treatment-related toxicities and compliance of the TNT schemas.

**Methods:**

A systematic review of randomized clinical trials and nonrandomized studies of interventions was performed as per Preferred Reporting Items for Systematic Reviews and Meta-Analyses (PRISMA)-NMA guidelines. A Bayesian NMA was conducted, and odds ratios (OR) with 95% credible intervals (CrI) are reported for all outcomes.

**Results:**

Eighteen studies including 5730 patients were identified. iTNT ranked highest on rate of rectal bleeding (cTNT: OR 0.23 95% CrI 0.05–0.93; nCRT: OR 0.33, 95% CrI 0.09–0.96), proctitis (cTNT: OR 0.2, 95% CrI 0.06–0.55; nCRT: OR 0.2, 95% CrI 0.06–0.51), and postoperative diarrhea (cTNT: OR 0.37, 95% CrI 0.18–0.73; nCRT: OR 0.33, 95% CrI 0.15–0.71); cTNT ranked highest on rate of vomiting (iTNT: OR 0.24, 95% CrI 0.05–0.96; nCRT: OR 0.29, 95% CrI 0.06–0.89) and a higher rate of lymphopenia than iTNT (iTNT: OR 0.56, 95% CrI 0.34–0.99). Radiotherapy compliance was highest in cTNT (iTNT: OR 0.23, 95% CrI 0.05–0.72; nCRT: OR 0.18, 95% CrI 0.04–0.58). There was no difference in overall toxicity and mortality, chemotherapy compliance, and remaining individual system-based toxicities and postoperative complications.

**Conclusions:**

Across all treatment strategies, iTNT had higher radiation-related gastrointestinal toxicities and postoperative diarrhea; cTNT had higher vomiting and lymphopenia rates. While no treatment strategy was superior in chemotherapy compliance, radiotherapy compliance was ranked highest in cTNT.

**Supplementary Information:**

The online version contains supplementary material available at 10.1245/s10434-025-17421-7.

Rectal cancer is the eighth most common cancer globally, with rising incidence in individuals younger than 50 years projected to quadruple by 2030.^1,2^ Around a third of such patients present with locally advanced rectal cancer (LARC), defined as transmural tumor invasion (T3 or T4) or node-positive malignancy without distant metastases.^3,4^.While traditional treatment of LARC, consisting of neoadjuvant chemoradiation therapy (nCRT) prior to surgery with selective adjuvant chemotherapy, achieves excellent local control, distant metastasis rates remain high at 25–30% within 5 years.^5–7^ A more recent therapeutic approach, known as total neoadjuvant therapy (TNT), delivers upfront neoadjuvant chemotherapy and radiotherapy,^6,8–11^ and evidence from randomized controlled trials (RCTs)^12–15^ and meta-analyses^6,9^ suggests improved metastases-free survival, enhanced tumor downstaging, and facilitated organ preservation compared with traditional nCRT alone. Consequently, TNT has become the standard of care for LARC.^16^

Despite its benefits, there are concerns that TNT may increase the risk of treatment-related toxicities, potentially reducing compliance, dose intensity, and overall survival.^17,18^ TNT is most commonly delivered as induction (chemotherapy followed by radiotherapy, iTNT) or consolidation (radiotherapy followed by chemotherapy, cTNT). While toxicities of chemotherapy and radiotherapy alone are well documented, the effect of treatment sequence on toxicity remains uncertain. Therefore, understanding this is crucial for tailoring treatment to individual patient needs.

Trials such as CAO/ARO/A10-12 and OPRA have highlighted differences in pathological complete response and organ preservation between iTNT and cTNT.^19,20^ Compliance appears to vary by sequence, with patients starting with radiotherapy less likely to complete chemotherapy, and vice versa. These findings underscore the need to balance efficacy with toxicity and compliance in TNT regimens.^15,21^

Currently, no NMAs have systematically evaluated the compliance and toxicities of TNT sequences comprehensively. Given the limited direct comparisons between iTNT and cTNT owing to their recency, we sought to perform an NMA to enable a pooled analysis of direct evidence between iTNT and cTNT, and indirect evidence across trials comparing each specific TNT regimen with traditional nCRT.^22^ Hence, this methodology offers a comprehensive synthesis of comparative ranking of compliance and toxicity patterns within TNT beyond individual trials and pairwise meta-analyses. This study aims to comprehensively evaluate treatment-related toxicities and compliance between different TNT regimens in LARC management.

## Methods

The systematic review adhered to the PRISMA-NMA^23^ guidelines (Appendix 1) and was carried out following the JBI methodology for systematic reviews.^24–26^ The protocol was prospectively registered with PROSPERO with assigned reference CRD42024491150.

### Search Strategy

In accordance to JBI methodology, an initial preliminary search of MEDLINE analyzed keywords in titles, abstracts, and index terms (Appendix 2).^27^ Following this, a systematic search used these terms across PubMed, Embase (Ovid), CINAHL, and Cochrane Central from 1 January 2012 to 17 April 2024 (Appendix 3). Citations were collated in EndNote 21 (Clarivate Analytics, PA, USA), duplicates were removed, and the results were uploaded to Covidence (Veritas Health Innovation Ltd, Melbourne, Australia). Two authors (W.S. and I.M.) independently screened titles and abstracts and excluded irrelevant studies. Eligible full texts were reviewed, and bibliographies were searched for additional studies. Discrepancies were resolved by a third reviewer (Z.B.), with exclusion reasons documented.

### Eligibility Criteria

RCTs and nonrandomized studies of interventions (NRSIs) of adult patients (16 years and above) with LARC (AJCC stage II/III^28^) who received iTNT, cTNT, or nCRT were included. Neoadjuvant chemotherapy was defined as four or more cycles of treatment. Outcomes measured were as followed:

- Overall toxicity (defined as National Cancer Institute (NCI) CTCA version 4.0 grade ≥ 3 treatment-related adverse events^29^)
- Compliance with chemotherapy (defined as patients that received ≥ 90% of the prescribed chemotherapy course)
- Compliance with radiotherapy (defined as patients that received ≥ 4500 Gy of the prescribed radiotherapy course)
- CTCA grade ≥ 3 individual treatment-related adverse events (i.e., gastrointestinal, neurological, cardiovascular, infection, immunological-related adverse events)
- Treatment-related mortality
- Postoperative complications (Clavien–Dindo grade 3 and above^30^)

### Data Extraction

Data extracted included patient demographics, treatment characteristics, and outcomes. All data were extracted into a predefined proforma in Microsoft Word (Microsoft Corporation, 2018) (Appendix 4). The extracted information was compared between the two reviewers (W.S. and I.M.), and any disagreements were resolved through consensus. All results underwent double data entry to reduce data extraction errors. Additionally, the authors of the included studies were contacted to request any missing or additional data.

### Assessment of Methodological Quality

Methodological quality assessment was performed independently by two investigators (W.S. and Z.B.). Risk of bias was assessed using the Risk of Bias (ROB) 2.0 tool for RCTs and the Risk of Bias in Nonrandomized Studies of Interventions (ROBINS-I) tool for NRSIs.^31,32^ Certainty of evidence for all outcomes was evaluated using the Grading of Recommendations Assessment, Development and Evaluation (GRADE) approach, and nonrandomized studies assessed with the ROBINS-I tool were initially rated high but downgraded for nonrandomized biases.^33^

### Data Synthesis

The network meta-analysis was performed within a Bayesian random-effects model, implemented via the *gemtc* package in R (R Foundation for Statistical Computing, Vienna, Austria).^34^ Noninformative priors were applied owing to uncertainty regarding treatment effectiveness. Convergence testing was performed by verifying that the Monte Carlo error remained below 5% of the standard deviation, and model fit was assessed through leverage plots and deviance information criterion (DIC).^35^ Convergence was achieved after 60,000 iterations, following an initial burn-in period of 30,000. DIC of fixed- and random-effects model were compared, and the model with the lower DIC value was selected. Odds ratio (OR) with 95% credible intervals (CrI) were calculated for categorical data.

Geometric network maps visualized direct comparisons between interventions, with nodes representing treatments and line thickness reflecting the number of comparisons.^36^ Rankograms and sum under the cumulative ranking (SUCRA) scores were generated to rank the likelihood of each intervention being classified from best to worst on the basis of effectiveness of the assessed outcome^36^.

Subgroup analyses focused on induction/consolidation chemotherapy regimens (i.e., FOLFOX, CAPOX, and FOLFIRINOX). Further subgroup analysis compared radiotherapy regimens (i.e., short-course radiotherapy versus long-course radiotherapy) and neoadjuvant chemoradiation agents (i.e., oral capecitabine versus infusional 5-FU). Where possible, RCTs and NRSIs were analyzed separately, with sensitivity analyses addressing high-bias studies. Funnel plots assessed publication bias for outcomes with 10+ studies.^37^.

A valid NMA relies on transitivity and consistency assumptions.^22^ To evaluate transitivity, we analyzed data regarding any interventions that could act as effect modifiers in each direct comparison across treatment groups. To evaluate consistency, a node-splitting model was utilized to compare direct evidence for a specific treatment against the indirect evidence obtained from the broader network.^38^

## Results

**Fig. 1:**
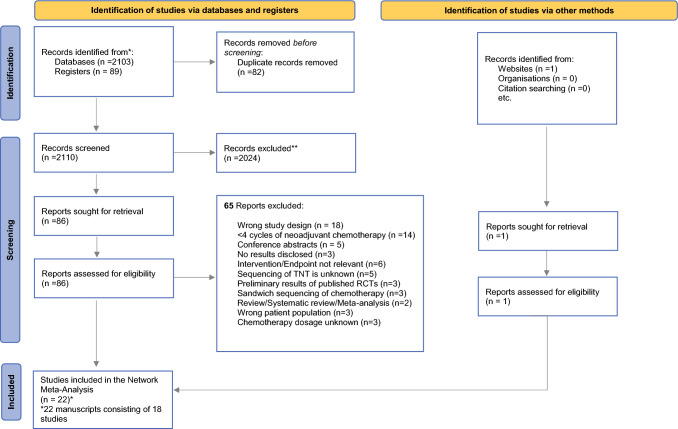
PRISMA 2020 flow diagram

Of 2192 identified articles, 18 (22 published manuscripts) met the inclusion criteria for the NMA. Eighty-two articles were removed owing to duplicates before screening. Out of 2110 articles, 2024 were excluded following title and abstract screening. A total of 86 articles were assessed for full-text analysis. Sixty-five articles did not meet inclusion criteria, with the majority of studies containing the wrong study design and utilizing ≤ 4 neoadjuvant chemotherapy cycles. The recently published long-term results of the UNICANCER-PRODIGE 23 trial (8 July 2024) were included after completion of the search strategy. The PRISMA flow diagram is summarized in Fig. 1.

### Characteristics of Included Studies

**Fig. 2:**
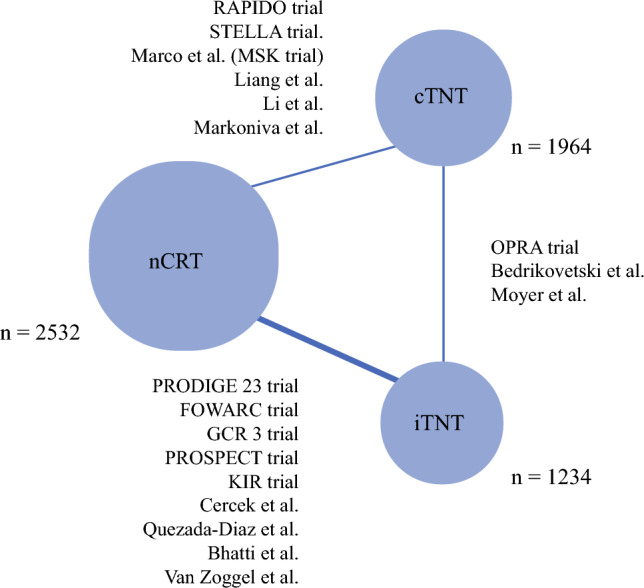
Geometrical Network Map of all included studies

**Fig. 3:**
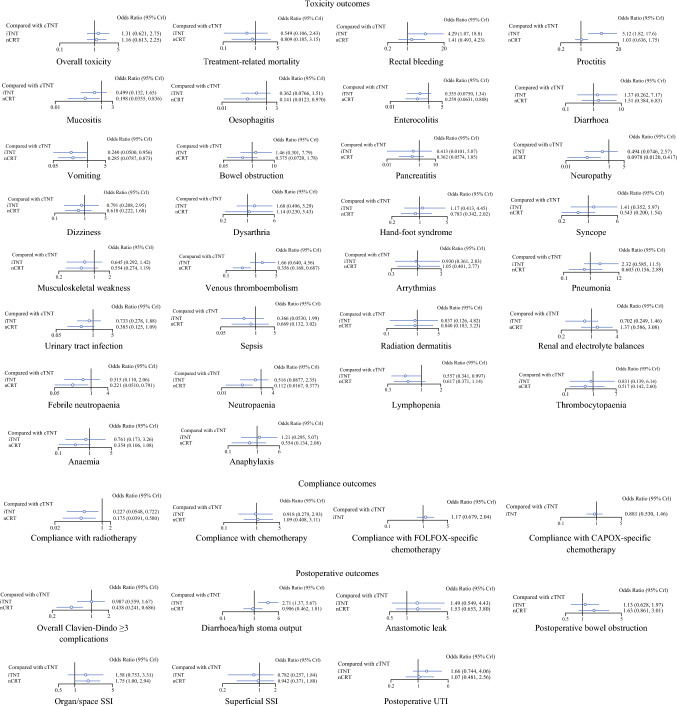
Forest plot for all outcomes

The included studies were published between 2006 and 2024. In total, 5730 patients were included in the study: cTNT (1964 patients), iTNT (1234 patients), and nCRT (2532 patients). A metwork map of all the included studies is summarized in Fig. 2. Direct comparisons from included studies (Table 1) and detailed study characteristics across each intervention (Appendix 5) are also summarized. One study within the cTNT–iTNT arm provided unpublished data that were included in our data analysis.^39^ Data were available for toxicity, compliance, and postoperative outcomes across all interventions. The rankogram for each outcome is summarized in Table 2. The number of included studies across each direct comparison for each outcome is summarized in Appendix 7. Relative estimates (Appendix 8) and forest plots (Fig. 3) for all outcomes are also summarized.

**Table 1:** Direct comparisons of included studies

Study	Type of study	Location	Interval recruitment	No. of patients				Follow-up (months/SD)*
				Overall	nCRT	iTNT	cTNT	
cTNT versus iTNT								
OPRA (2022) Garcia-Aguilar et al.^60^	RCT	USA	2014–2020	324	–	158	166	36
Moyer et al. (2023)^44^	Retrospective cohort study	USA	2016–2020	167	–	83	84	–
Bedrikovetski et al. (2023)^39^	Prospective cohort study	Australia	2019–2022	79	–	33	46	24 (9.1)
cTNT versus nCRT								
RAPIDO (2020) Bahadoer et al.^12^	RCT	Netherlands	2011–2016	912	450	–	462	55.2 (3.7)
STELLAR (2022) Jin et al.^42^	RCT	China	2015–2018	599	297	–	302	35 (9.0)
Li et al. (2017)^50^	RCT	China	2012–2015	80	40	–	40	30
Markoniva et al. (2017)^43^	RCT—Pair-matched subset analysis	USA	2009–2012	138	69	–	69	49
MSKCC Trial Marco et al. (2018)^51^ Garcia-Aguilar et al. (2015)^61^	Quasi-randomised trial	USA	2004–2012	125	60	–	65	59 (22.0)
Liang et al. (2019)^45^	Retrospective cohort study	China	2010–2016	156	80	–	76	31 (15.0)
iTNT versus nCRT								
UNICANCER-PRODIGE 23 Conroy et al. (2020)^13^ Conroy et al. (2024)^62^	RCT	France	2012–2017	461	230	231	–	46.5 (4.4)
GCR 3 Fernándes-Martos et al. (2010)^63^ Fernándes-Martos et al. (2015)^48^	RCT	Spain	2006–2007	108	52	56	–	69.5
FORWAC (2019) Deng et al.^53^	RCT	China	2010–2015	330	165	165	–	45.2 (14.2)
PROSPECT (2023) Schrag et al.^52^	RCT	Canada, USA, Switzerland	2012–2018	1128	543	585	–	58
KIR (2020) Aurelie-Garant et al.^41^	RCT	Canada	2010–2017	180	60	120	–	63
Cercek et al. (2018)^10^	Retrospective cohort	USA	2009–2015	628	320	308	–	24
Quezada-Diaz et al. (2019)^49^	Retrospective cohort	USA	2011–2017	132	34	98	–	22 (7.7)
Bhatti et al. (2015)^47^	Retrospective cohort	Pakistan	2005–2011	154	61	93	–	46 (19.0)
Van Zoggel et al. (2018)^40^	Retrospective cohort	Netherlands	2010–2016	129	71	58	–	36

*Values are mean (SD)

*nCRT* neoadjuvant chemoradiation, *iTNT* induction TNT, *cTNT* consolidation TNT

–Represents N/A or no data available

**Table 2:** Probability of each treatment ranking from best (first) to worst (third) according to surface under the cumulative ranking curve (SUCRA) values

Outcomes	First	Second	Third
Toxicity outcomes			
Overall grade III and above treatment-related adverse events	iTNT: 0.59	nCRT: 0.50	cTNT: 0.62
Rate of treatment-related mortality	cTNT: 0.56	nCRT: 0.48	iTNT: 0.64
Individual grade III and above treatment-related adverse events			
Gastrointestinal			
Diarrhea	nCRT: 0.45	iTNT: 0.39	cTNT: 0.57
Mucositis	cTNT: 0.94	iTNT: 0.82	nCRT: 0.87
Esophagitis	cTNT: 0.91	iTNT: 0.75	nCRT: 0.82
Enterocolitis	cTNT: 0.92	iTNT: 0.63	nCRT: 0.61
Vomiting	cTNT: 0.97	nCRT: 0.60	iTNT: 0.62
Nausea without vomiting	iTNT: 0.55	cTNT: 0.47	nCRT: 0.80
Bowel obstruction	iTNT: 0.68	cTNT: 0.60	nCRT: 0.88
Pancreatitis	cTNT: 0.66	cTNT: 0.31	nCRT: 0.51
Proctitis	iTNT: 0.98	nCRT: 0.75	cTNT: 0.54
Rectal bleeding	iTNT: 0.96	nCRT: 0.72	cTNT: 0.74
Neurological			
Dizziness	cTNT: 0.36	iTNT: 0.55	nCRT: 0.57
Dysarthria	iTNT: 0.56	nCRT: 0.40	cTNT: 0.48
Hand–foot syndrome	iTNT: 0.57	cTNT: 0.52	nCRT: 0.60
Neuropathy	cTNT: 0.81	iTNT: 0.80	nCRT: 0.99
Syncope	iTNT: 0.62	cTNT: 0.68	nCRT: 0.81
Musculoskeletal	cTNT: 0.86	iTNT: 0.65	nCRT: 0.72
Cardiovascular			
Arrythmia	nCRT: 0.40	cTNT: 0.47	iTNT: 0.51
Venous thromboembolism	iTNT: 0.86	cTNT: 0.86	nCRT: 0.99
Infections			
Pneumonia	iTNT: 0.85	cTNT: 0.68	nCRT: 0.74
Urinary tract infection	cTNT: 0.74	iTNT: 0.68	nCRT: 0.80
Sepsis	cTNT: 0.66	nCRT: 0.55	iTNT: 0.73
Dermatological			
Radiation dermatitis	cTNT: 0.40	iTNT: 0.38	nCRT: 0.41
Renal and electrolyte imbalances	nCRT: 0.76	cTNT: 0.65	iTNT: 0.80
Immunological			
Febrile neutropenia	cTNT: 0.83	iTNT: 0.77	nCRT: 0.94
Neutropenia	cTNT: 0.84	iTNT: 0.83	nCRT: 0.98
Lymphopenia	cTNT: 0.94	nCRT: 0.67	iTNT: 0.70
Thrombocytopenia	cTNT: 0.53	iTNT: 0.36	nCRT: 0.61
Anemia	cTNT: 0.64	iTNT: 0.55	nCRT: 0.87
Anaphylaxis	iTNT: 0.58	cTNT: 0.45	nCRT: 0.75
Compliance outcomes			
Compliance with radiotherapy (≥ 4500 Gy)	cTNT: 0.68	iTNT: 0.67	nCRT: 0.90
Compliance with chemotherapy (overall) > 90%	iTNT: 0.62	cTNT: 0.38	nCRT: 0.42
Compliance with FOLFOX chemotherapy (> 90%)	iTNT: 0.71	cTNT:0.69	N/A
Compliance with CAPOX chemotherapy (> 90%)	cTNT: 0.65	iTNT: 0.51	N/A
Postoperative outcomes			
Overall Clavien–Dindo grade III and above postoperative complications	cTNT: 0.49	iTNT: 0.52	nCRT: 0.99
Individual Clavien–Dindo grade III and above postoperative complications			
Anastomotic leak	nCRT: 0.42	iTNT: 0.48	cTNT: 0.72
High stoma output/diarrhea	iTNT: 0.99	cTNT: 0.65	nCRT: 0.61
Postoperative bowel obstruction	nCRT: 0.89	iTNT: 0. 64	cTNT: 0.65
Organ/space surgical site infection (SSI)	nCRT: 0.60	iTNT: 0.50	cTNT: 0.88
Superficial incisional SSI	cTNT: 0.51	nCRT: 0.48	iTNT: 0.59
Urinary tract infection	iTNT: 0.69	nCRT: 0.49	cTNT: 0.55

*nCRT* neoadjuvant chemoradiation, *iTNT* induction TNT, *cTNT* consolidation TNT

### Risk of Bias Assessment

Risk of bias assessments for RCTs and NRSIs are summarized in Appendix 6. Nine studies (50%) had low risk, seven (39%) had moderate risk, and two (11%) had high risk of bias. Four RCTs had moderate risk due to deviations from intended outcomes, largely from inevitable patient unblinding, contributing to potential bias.

### Toxicity Outcomes

#### Overall Toxicity and Treatment-Related Mortality

Across all interventions, there were no statistically significant difference in the rate of overall toxicity and treatment-related mortality (Fig. 3).

#### Gastrointestinal-Related Toxicity

Across all interventions, iTNT ranked highest in grade ≥ 3 rectal bleeding and proctitis; however, there were no statistically significant difference between cTNT and nCRT (Table 2; Fig. 3). There was no statistically significant difference in grade ≥ 3 mucositis, esophagitis, and enterocolitis between iTNT and cTNT (Fig. 3). However, compared with nCRT, cTNT had higher rates of grade ≥ 3 mucositis, esophagitis, and enterocolitis, but no difference was found between iTNT–nCRT (Appendix 8). Across all interventions, cTNT ranked highest in grade ≥ 3 vomiting, but there was no difference between iTNT–nCRT (Table 2; Fig. 3). There was no statistically significant difference in grade ≥ 3 diarrhea, bowel obstruction, and pancreatitis across all three treatment strategies (Fig. 3; Appendix 8).

#### Neurological-Related Toxicity

Both TNT sequences had higher rates of grade ≥ 3 neuropathy as compared with nCRT; however, there was no difference between iTNT and cTNT (Fig. 3; Appendix 8). There was no statistically significant difference in grade ≥ 3 dizziness, dysarthria, hand–foot syndrome (HFS), syncope, and musculoskeletal weakness across all three treatment strategies (Fig. 3; Appendix 8).

#### Cardiovascular-Related Toxicity

Both TNT sequences had higher rates of grade ≥ 3 venous thromboembolism (VTE) as compared with nCRT; however, there was no difference between iTNT and cTNT (Fig. 3; Appendix 8). There was no statistically significant difference in grade ≥ 3 arrythmias across all three treatment strategies (Fig. 3; Appendix 8).

#### Infection-Related Toxicity

There was no statistically significant difference in grade ≥ 3 pneumonia, urinary tract infection (UTI), and sepsis across all three treatment strategies (Fig. 3; Appendix 8).

#### Skin-Related Toxicity

There was no statistically significant difference in grade ≥ 3 radiation dermatitis across all three treatment strategies (Fig. 3; Appendix 8).

#### Renal and Electrolyte Disturbances

There was no statistically significant difference in renal dysfunction and electrolyte derangement across all three treatment strategies (Fig. 3; Appendix 8).

#### Immunological-Related Toxicity

cTNT had higher rates of grade ≥ 3 febrile neutropenia compared with nCRT; however, there were no difference between iTNT–cTNT and iTNT–nCRT (Fig. 3; Appendix 8). Both TNT sequences had higher rates of neutropenia (defined as absolute neutrophil count less than 0.5 × 10^9^/L) as compared with nCRT; however, there were no difference between iTNT and cTNT (Fig. 3; Appendix 8). cTNT had higher rates of lymphopenia (defined as absolute lymphocyte count less than 1.0 × 10^9^/L) compared with iTNT; however, there were no difference between both TNT sequences compared with nCRT (Fig. 3; Appendix 8). There was no statistically significant difference in thrombocytopenia, anemia, and anaphylaxis across all three treatment strategies (Fig. 3; Appendix 8).

### Compliance Outcomes

cTNT ranked highest in compliance with radiotherapy (Table 2; Fig. 3), however, there were no statistically significant difference between iTNT and nCRT. There was no statistically significant difference across all treatment strategies in compliance with chemotherapy (Fig. 3; Appendix 8).

### Subgroup Analysis on Specific Induction/Consolidation Chemotherapy Agents

There was no statistically significant difference between cTNT and iTNT in FOLFOX- and CAPOX-specific chemotherapy compliance (Fig. 3; Appendix 8). Only the PRODIGE-23 trial adopted a FOLFIRINOX-based chemotherapy, and therefore a subgroup analysis of FOLFIRINOX-specific chemotherapy compliance was not possible.

### Postoperative Outcomes

#### Overall Postoperative Complications

There was no statistically significant difference in overall Clavien–Dindo (CD) grade ≥ 3 postoperative complications between cTNT and iTNT. However, both TNT sequences had higher rates of overall postoperative complications compared with nCRT (Table 2; Fig. 3).

#### Individual Postoperative Complications

Across all interventions, iTNT ranked highest in CD grade ≥ 3 postoperative diarrhea and/or high stoma output; however, there were no statistically significant difference between cTNT and nCRT (Table 2; Fig. 3). There were no statistically significant difference in CD grade ≥ 3 postoperative anastomotic leak, bowel obstruction, organ/space surgical site infection (SSI), superficial surgical site infection (SSI), and UTI across all three treatment strategies (Fig. 3; Appendix 8).

Postoperative ileus, pelvic fibrosis, colonic ischemia, VTE, 30-day readmission, mortality, and reoperation were not measured owing to the paucity of data.

### Further Subgroup Analysis

#### Short-Course versus Long-Course Radiotherapy

Six studies utilized short-course radiotherapy (25Gy).^12,40–44^ The subgroup analysis performed with only treatment regimens comprising long-course radiotherapy (≥ 50 Gy) showed no difference from the main analysis in terms of chemotherapy and radiotherapy compliance, toxicity, and postoperative outcomes (Appendix 9). However, the subgroup analysis of treatment regimens comprising short-course radiotherapy (SCRT) showed no difference in radiotherapy compliance across all treatment regimens (Appendix 9). All other chemotherapy compliance, toxicity, and postoperative outcomes in the SCRT subgroup showed no differences as compared with the main analysis.

#### Neoadjuvant Chemoradiation Agents (Oral Capecitabine versus Infusional 5-FU)

Twelve studies utilized oral capecitabine,^12,21,39,40,42–49^ and ten studies utilized infusional 5-FU^10,21,39,41,43,44,50–53^ in the nCRT arm. Subgroup analysis of oral capecitabine nCRT showed no difference in compliance, toxicity, and postoperative outcomes as compared with the main analysis (Appendix 9). While cTNT ranked highest in compliance with radiotherapy in the main analysis, subgroup analysis of infusional 5-FU as the nCRT agent showed no statistically significant difference in radiotherapy compliance across all treatment regimens (Appendix 9).

#### Inconsistency, Transitivity, and GRADE Assessment

Node-splitting models of inconsistency (Appendix 10) were performed for all outcomes. Inconsistencies were detected across all three comparators for grade ≥ 3 mucositis and pneumonia. All other node-splitting inconsistency *p* values were > 0.05.

The transitivity assumption was analyzed for all three comparators as summarized in Appendix 11. There were no major deviations in patient characteristics (i.e., age, gender, and Eastern Cooperative Oncology Group (ECOG) status) and treatment characteristics (i.e., median radiation dose, chemotherapy type, and interval between chemotherapy and radiation) across included studies.

GRADE assessment for all outcomes is summarized in Appendix 12. Additional details on GRADE analyses are outlined in Appendix 13. The overall quality of toxicity, compliance and postoperative outcomes was deemed moderate.

### Sensitivity Analysis

Additional sensitivity analyses for primary outcomes excluded studies with unclear or high bias risk. Results remained consistent with the main analysis, showing no significant differences.

### Publication Bias

Funnel plots generated for outcomes including ≥ 10 studies showed no evidence of asymmetry in the following outcomes (Appendix 14): overall toxicity, rectal bleeding, proctitis, diarrhea, neuropathy, hand–foot syndrome, arrythmias, renal and electrolyte imbalances, neutropenia, lymphopenia, thrombocytopenia, anemia, anaphylaxis, compliance with chemotherapy, and compliance with FOLFOX-specific chemotherapy. The remaining outcomes were not assessed owing to insufficient studies.

## Discussion

This review is the first to explore an extensive list of toxicity and compliance across studies of various TNT sequences, incorporating available RCTs and NRSIs to enable direct and indirect treatment comparisons. Our results suggest that iTNT had the highest rate of rectal bleeding and proctitis while cTNT had the highest rate of vomiting. cTNT had higher rates of lymphopenia compared with iTNT. Compared with nCRT, both TNT sequences had higher rates of neuropathy, VTE, and neutropenia, while cTNT had higher rates of mucositis, enterocolitis, esophagitis, and febrile neutropenia. No difference was found across overall toxicity and the remaining individual toxicity outcomes in all treatment strategies.

Tailoring TNT sequences on the basis of toxicity profiles is crucial for individualizing treatment. This review highlights higher rates of radiotherapy-related GI toxicities (i.e., proctitis and rectal bleeding) in iTNT, suggesting that cTNT may be preferable for patients with GI comorbidities or higher risk (i.e., elderly or those with inflammatory bowel disease (IBD)). Alternatively, for select iTNT patients, strategies such as tailored radiation dose, adapted treatment protocols, or prophylactic measures (i.e., dietary modifications and anti-inflammatory agents) may help attenuate GI toxicities. Predicting and risk-stratifying toxicities by treatment sequence could minimize interruptions and improve quality of life.^54^

Radiotherapy compliance was highest in cTNT, while chemotherapy compliance remained similar across all strategies. Clinically, improved radiotherapy compliance in cTNT may enhance local control of LARC as alluded to in key trials.^55,56^ Meanwhile, comparable chemotherapy adherence allows flexibility in sequencing based on patient preferences and institutional protocols. Nonetheless, several TNT protocols in included studies used short-course (25 Gy in 5 fractions) radiotherapy as compared with long-course (50.4 Gy in 28 fractions) radiotherapy in the control arm.^12,42,44^ Accounting for this heterogeneity, our subgroup analysis of long-course radiotherapy studies showed similar compliance trends.

Chemotherapy delivery in TNT also varies by regimen (i.e., FOLFOX, CAPOX, and FOLFIRINOX) and nCRT agents (i.e., oral capecitabine and infusional 5-FU), but subgroup analysis showed no differences in chemotherapy compliance. However, when limited to infusional 5-FU, radiotherapy compliance was similar across all regimens, unlike the main analysis, where cTNT ranked highest.

This may be influenced by more cTNT studies using oral capecitabine, often with short-course radiotherapy.^12,42,43^ Furthermore, capecitabine’s oral convenience, toxicity profile, and better integration with radiotherapy may contribute to improved adherence.^57,58^

Our study design included only TNT studies with at least four chemotherapy cycles. This aimed to reflect standard LARC treatment, which commonly involves 5–8 cycles of CAPOX or 7–8 cycles of FOLFOX. Similarly, “sandwich” TNT trials were excluded as they remain broadly exploratory. Furthermore, both RCTs and NRSIs were included—a decision guided by Cochrane guidelines—ensuring that rare but severe toxicities (i.e., grade ≥ 3) were captured, which RCTs alone may not adequately assess owing to smaller sample sizes and shorter follow-up period.^59^

The transitivity and consistency assumptions must be carefully reviewed, especially in TNT studies with potential effect modifiers (e.g., chemotherapy and radiation dosage). While our analyses suggest no major deviations, unknown variables such as institutional protocols or population differences may still influence treatment effects. These factors underscore the complexity of NMA where maintaining these assumptions can be challenging, even when effect modifiers appear well matched.

A key limitation is the small sample sizes for toxicity outcomes, reducing precision and increasing uncertainty. For instance, the wide credibility interval (0.3–7.8) for bowel obstruction between cTNT and iTNT suggests that the true effect size is less precisely estimated and lies broadly within a wide range. Additionally, inconsistent outcome definitions, such as renal dysfunction, and challenges in distinguishing treatment effects from comorbidities or cancer may confound results. Variability in follow-up duration across studies also complicates the assessment of acute versus chronic toxicities, as the latter is less explored given the relative youth of TNT.

Lastly, we emphasize the necessity for future high-powered trial-level data on individual treatment-related toxicities. Current evidence often aggregates toxicity data cumulatively rather than distinguishing specific adverse events, which limits clinical applicability. Until such evidence becomes available, our results are important to guide clinicians in choosing a personalized regimen that strikes an optimal balance between effectiveness and toxicity.

Therefore, the findings of this NMA reinforce that no single approach—induction or consolidation—is universally superior in toxicity or compliance. Rather, it underpins the importance of patient selection in determining the most appropriate TNT sequencing, weighing the survival benefits and the risk of individual treatment-related adverse events.

## Supplementary Information

Below is the link to the electronic supplementary material.

Supplementary file1 (DOCX 4138 KB)

## Protocol Registration

PROSPERO CRD42024491150.
