# Supplementary material for: Compliance and Toxicity of Total Neoadjuvant Therapy in Locally Advanced Rectal Cancer: A Systematic Review and Network Meta-analysis
Source: Ann Surg Oncol. 2025 May 5;32(9):6728–39. doi: 10.1245/s10434-025-17421-7 (PMC12317888; doi:10.1245/s10434-025-17421-7)
Supplement: Supplementary file 1 — Supplementary file1 (DOCX 4138 KB) [file 10434_2025_17421_MOESM1_ESM.docx]

**Compliance and Toxicity of Total Neoadjuvant Therapy in Locally Advanced Rectal Cancer: A Systematic Review and Network Meta-analysis**

Warren Seow, MBBS, MClinSc, Ishraq Murshed, MBBS, MS, Zachary Bunjo, MBBS, MS, Sergei Bedrikovetski, BHSc (Hons), PhD, Jennifer Stone BPsySci, GCPH, MEpi (Clin), PhD, Tarik Sammour, MBChB, FRACS, PhD

Table of contents

[Appendix 1: PRISMA-NMA Checklist of Items 2](#_Toc192498113)

[Appendix 2: Initial Search Terms 7](#_Toc192498114)

[Appendix 3: Additional Information on Search Strategies 8](#_Toc192498115)

[Appendix 4: Data extraction template 11](#_Toc192498116)

[Appendix 5: Detailed Characteristics of the Included Studies 14](#_Toc192498117)

[Appendix 6: Risk of Bias analysis 24](#_Toc192498118)

[Appendix 7: Geometrical network maps for all outcomes 25](#_Toc192498119)

[Appendix 8. League table of network meta-analysis relative estimates for all treatment comparison 34](#_Toc192498120)

[Appendix 9: Further subgroup analysis 41](#_Toc192498121)

[Appendix 10: Node-splitting analysis for inconsistencies across treatment comparisons 45](#_Toc192498122)

[Appendix 11: Transitivity analysis 56](#_Toc192498123)

[Appendix 12: GRADE assessment Summary of Findings of all outcomes 0](#_Toc192498124)

[Appendix 13: Additional details of GRADE assessment to assess certainty of findings 9](#_Toc192498125)

[Appendix 14: Funnel plot of all outcomes 0](#_Toc192498126)

## Appendix 1: PRISMA-NMA Checklist of Items

| **Section/Topic** | **Item #** | **Checklist Item** | **Reported on Page #** |
| --- | --- | --- | --- |
| **TITLE** |  |  |  |
| Title | 1 | Identify the report as a systematic review *incorporating a network meta-analysis (or related form of meta-analysis).* | ***1*** |
|  |  |  |  |
| **ABSTRACT** |  |  |  |
| Structured summary | 2 | Provide a structured summary including, as applicable:  **Background:** main objectives  **Methods:** data sources; study eligibility criteria, participants, and interventions; study appraisal; and *synthesis methods, such as network meta-analysis.*  **Results:** number of studies and participants identified; summary estimates with corresponding confidence/credible intervals; *treatment rankings may also be discussed. Authors may choose to summarize pairwise comparisons against a chosen treatment included in their analyses for brevity.*  **Discussion/Conclusions:** limitations; conclusions and implications of findings.  **Other:** primary source of funding; systematic review registration number with registry name. | 2-3 |
|  |  |  |  |
| **INTRODUCTION** |  |  |  |
| Rationale | 3 | Describe the rationale for the review in the context of what is already known*, including mention of why a network meta-analysis has been conducted.* | ***4*** |
| Objectives | 4 | Provide an explicit statement of questions being addressed, with reference to participants, interventions, comparisons, outcomes, and study design (PICOS). | 5 |
|  |  |  |  |
| **METHODS** |  |  |  |
| Protocol and registration | 5 | Indicate whether a review protocol exists and if and where it can be accessed (e.g., Web address); and, if available, provide registration information, including registration number. | 6 |
| Eligibility criteria | 6 | Specify study characteristics (e.g., PICOS, length of follow-up) and report characteristics (e.g., years considered, language, publication status) used as criteria for eligibility, giving rationale. *Clearly describe eligible treatments included in the treatment network, and note whether any have been clustered or merged into the same node (with justification).* | ***6*** |
| Information sources | 7 | Describe all information sources (e.g., databases with dates of coverage, contact with study authors to identify additional studies) in the search and date last searched. | 7 |
| Search | 8 | Present full electronic search strategy for at least one database, including any limits used, such that it could be repeated. | 6 |
| Study selection | 9 | State the process for selecting studies (i.e., screening, eligibility, included in systematic review, and, if applicable, included in the meta-analysis). | 6 |
| Data collection process | 10 | Describe method of data extraction from reports (e.g., piloted forms, independently, in duplicate) and any processes for obtaining and confirming data from investigators. | 7 |
| Data items | 11 | List and define all variables for which data were sought (e.g., PICOS, funding sources) and any assumptions and simplifications made. | 7 |
| **Geometry of the network** | **S1** | Describe methods used to explore the geometry of the treatment network under study and potential biases related to it. This should include how the evidence base has been graphically summarized for presentation, and what characteristics were compiled and used to describe the evidence base to readers. | ***8*** |
| Risk of bias within individual studies | 12 | Describe methods used for assessing risk of bias of individual studies (including specification of whether this was done at the study or outcome level), and how this information is to be used in any data synthesis. | 7 |
| Summary measures | 13 | State the principal summary measures (e.g., risk ratio, difference in means). *Also describe the use of additional summary measures assessed, such as treatment rankings and surface under the cumulative ranking curve (SUCRA) values, as well as modified approaches used to present summary findings from meta-analyses.* | 7 |
| Planned methods of analysis | 14 | Describe the methods of handling data and combining results of studies for each network meta-analysis. This should include, but not be limited to:   - *Handling of multi-arm trials;* - *Selection of variance structure;* - *Selection of prior distributions in Bayesian analyses; and* - *Assessment of model fit.* | 8 |
| **Assessment of Inconsistency** | **S2** | Describe the statistical methods used to evaluate the agreement of direct and indirect evidence in the treatment network(s) studied. Describe efforts taken to address its presence when found. | 8 |
| Risk of bias across studies | 15 | Specify any assessment of risk of bias that may affect the cumulative evidence (e.g., publication bias, selective reporting within studies). | **7** |
| Additional analyses | 16 | Describe methods of additional analyses if done, indicating which were pre-specified. This may include, but not be limited to, the following:   - Sensitivity or subgroup analyses; - Meta-regression analyses; - *Alternative formulations of the treatment network; and* - *Use of alternative prior distributions for Bayesian analyses (if applicable).* | ***8*** |
|  |  |  |  |
| **RESULTS†** |  |  |  |
| Study selection | 17 | Give numbers of studies screened, assessed for eligibility, and included in the review, with reasons for exclusions at each stage, ideally with a flow diagram. | 9 |
| **Presentation of network structure** | **S3** | Provide a network graph of the included studies to enable visualization of the geometry of the treatment network. | ***9*** |
| **Summary of network geometry** | **S4** | Provide a brief overview of characteristics of the treatment network. This may include commentary on the abundance of trials and randomized patients for the different interventions and pairwise comparisons in the network, gaps of evidence in the treatment network, and potential biases reflected by the network structure. | ***9*** |
| Study characteristics | 18 | For each study, present characteristics for which data were extracted (e.g., study size, PICOS, follow-up period) and provide the citations. | 9 |
| Risk of bias within studies | 19 | Present data on risk of bias of each study and, if available, any outcome level assessment. | 9 |
| Results of individual studies | 20 | For all outcomes considered (benefits or harms), present, for each study: 1) simple summary data for each intervention group, and 2) effect estimates and confidence intervals. *Modified approaches may be needed to deal with information from larger networks.* | ***10-12*** |
| Synthesis of results | 21 | Present results of each meta-analysis done, including confidence/credible intervals. *In larger networks, authors may focus on comparisons versus a particular comparator (e.g. placebo or standard care), with full findings presented in an appendix. League tables and forest plots may be considered to summarize pairwise comparisons.* If additional summary measures were explored (such as treatment rankings), these should also be presented. | ***10-12*** |
| **Exploration for inconsistency** | **S5** | Describe results from investigations of inconsistency. This may include such information as measures of model fit to compare consistency and inconsistency models, *P* values from statistical tests, or summary of inconsistency estimates from different parts of the treatment network. | ***13*** |
| Risk of bias across studies | 22 | Present results of any assessment of risk of bias across studies for the evidence base being studied. | 9 |
| Results of additional analyses | 23 | Give results of additional analyses, if done (e.g., sensitivity or subgroup analyses, meta-regression analyses*, alternative network geometries studied, alternative choice of prior distributions for Bayesian analyses,* and so forth). | ***13*** |
|  |  |  |  |
| **DISCUSSION** |  |  |  |
| Summary of evidence | 24 | Summarize the main findings, including the strength of evidence for each main outcome; consider their relevance to key groups (e.g., healthcare providers, users, and policy-makers). | 14 |
| Limitations | 25 | Discuss limitations at study and outcome level (e.g., risk of bias), and at review level (e.g., incomplete retrieval of identified research, reporting bias). *Comment on the validity of the assumptions, such as transitivity and consistency. Comment on any concerns regarding network geometry (e.g., avoidance of certain comparisons).* | 16 |
| Conclusions | 26 | Provide a general interpretation of the results in the context of other evidence, and implications for future research. | 16 |
|  |  |  |  |
| **FUNDING** |  |  |  |
| Funding | 27 | Describe sources of funding for the systematic review and other support (e.g., supply of data); role of funders for the systematic review. This should also include information regarding whether funding has been received from manufacturers of treatments in the network and/or whether some of the authors are content experts with professional conflicts of interest that could affect use of treatments in the network. | ***1*** |

PICOS = population, intervention, comparators, outcomes, study design.

* Text in italics indicateS wording specific to reporting of network meta-analyses that has been added to guidance from the PRISMA statement.

† Authors may wish to plan for use of appendices to present all relevant information in full detail for items in this section.

## Appendix 2: Initial Search Terms

*PubMed*

Search term:

(“rectal cancer” OR “rectal adenocarcinoma”) AND (“total neoadjuvant treatment” OR “total neoadjuvant therapy” OR “neoadjuvant therapy” OR “neoadjuvant chemotherapy”) AND (“induction chemotherapy” OR “consolidation chemotherapy” OR “preoperative chemotherapy” OR “chemotherapy” OR “chemoradiotherapy” OR “chemoradiation”)

*("rectal neoplasms"[MeSH Terms] OR ("rectal"[All Fields] AND "neoplasms"[All Fields]) OR "rectal neoplasms"[All Fields] OR ("rectal"[All Fields] AND "cancer"[All Fields]) OR "rectal cancer"[All Fields] AND ("total"[All Fields] OR "totaled"[All Fields] OR "totaling"[All Fields] OR "totalled"[All Fields] OR "totalling"[All Fields] OR "totals"[All Fields]) AND ("neoadjuvant therapy"[MeSH Terms] OR ("neoadjuvant"[All Fields] AND "therapy"[All Fields]) OR "neoadjuvant therapy"[All Fields]) AND (("consolidation chemotherapy"[MeSH Terms] OR ("consolidation"[All Fields] AND "chemotherapy"[All Fields]) OR "consolidation chemotherapy"[All Fields]) OR ("induction chemotherapy"[MeSH Terms] OR ("induction"[All Fields] AND "chemotherapy"[All Fields]) OR "induction chemotherapy"[All Fields]) OR ("chemoradiotherapy"[MeSH Terms] OR "chemoradiotherapy"[All Fields] OR "chemoradiation"[All Fields]))*

No language and date restrictions

Results: 1059 records retrieved (April 17, 2024)

## Appendix 3: Additional Information on Search Strategies

Date searched: April 17, 2024

Restrictions: No language restrictions, human studies, date limit from January 1, 2012 to April 17, 2024

Total studies included for Screening: 2192 (excluding 1095 duplicates)

| **Sources** | **Keywords** | **Limit** | **Results** |
| --- | --- | --- | --- |
| PubMed | ("rectal cancer" OR "rectal adenocarcinoma") AND ("total neoadjuvant treatment" OR "total neoadjuvant therapy" OR "neoadjuvant therapy" OR "neoadjuvant chemotherapy") AND ("induction chemotherapy" OR "consolidation chemotherapy" OR "preoperative chemotherapy" OR "chemotherapy") OR ("chemoradiotherapy" OR "chemoradiation") | Studies in the last 11 years, Humans, No language restrictions | 826 |
| EMBASE | (("rectal cancer" or "rectal adenocarcinoma") AND ("total neoadjuvant treatment" or "total neoadjuvant therapy" or "neoadjuvant therapy" or "neoadjuvant chemotherapy") AND ("induction chemotherapy" or "consolidation chemotherapy" or "preoperative chemotherapy" or "chemotherapy") or ("chemoradiotherapy" or "chemoradiation")).mp. [mp=title, abstract, heading word, drug trade name, original title, device manufacturer, drug manufacturer, device trade name, keyword heading word, floating subheading word, candidate term word] | (human and yr="2012 - 2024"), "Article" [Publication Type] | 1315 |
| CINAHL | ("rectal cancer" OR "rectal adenocarcinoma") AND ("total neoadjuvant treatment" OR "total neoadjuvant therapy" or "neoadjuvant therapy" or "neoadjuvant chemotherapy") AND ("induction chemotherapy" OR "consolidation chemotherapy" OR "preoperative chemotherapy" OR "chemotherapy") OR ("chemoradiotherapy" OR "chemoradiation") | Published Date: 20120101-20240217, Research Article, Publication Type: Journal Article | 633 |
| Cochrane Central Register of Controlled Trials | ("rectal cancer" OR "rectal adenocarcinoma") AND ("total neoadjuvant treatment" OR "total neoadjuvant therapy" OR "neoadjuvant therapy" OR "neoadjuvant chemotherapy") AND ("induction chemotherapy" OR "consolidation chemotherapy" OR "preoperative chemotherapy" OR "chemotherapy") OR ("chemoradiotherapy" OR "chemoradiation") | None | 211 |
| Web of Science | ("rectal cancer" OR "rectal adenocarcinoma") AND ("total neoadjuvant treatment" OR "total neoadjuvant therapy" OR "neoadjuvant therapy" OR "neoadjuvant chemotherapy") AND ("induction chemotherapy" OR "consolidation chemotherapy" OR "preoperative chemotherapy" OR "chemotherapy") OR ("chemoradiotherapy" OR "chemoradiation") | Document types: Articles, Languages: All Language, Timespan:2012-01-01 to 2024-02-17 | 302 |

## Appendix 4: Data extraction template

| Basic characteristic of the included studies | | | | | | | | | |
| --- | --- | --- | --- | --- | --- | --- | --- | --- | --- |
| Study | Type of study | Country | Interval Recruitment | Type of Intervention | Type of Comparator | Number of participants | | | Follow-up (months) |
|  |  |  |  |  |  | Overall | Intervention arm | Control arm |  |
|  |  |  |  |  |  |  |  |  |  |

| Detailed characteristics of the included studies | | | | | | | | | |
| --- | --- | --- | --- | --- | --- | --- | --- | --- | --- |
| Study | Neoadjuvant Intervention | Characteristic of chemotherapy | | Characteristic of radiotherapy/chemoradiation | | Patient characteristics* | | Oncological characteristics | |
|  |  | Regimen | No. of Cycles | Median Radiation dose, cGy (IQR) | | Gender  M:F | Age (range/SD) | TNM Staging | Distance from anal verge, cm (range/SD) |
|  | Intervention |  |  |  |  |  |  |  |  |
|  | Control |  |  |  |  |  |  |  |  |

| Treatment-related toxicity and mortality outcomes of eligible studies | | | | | | | | | | | | |
| --- | --- | --- | --- | --- | --- | --- | --- | --- | --- | --- | --- | --- |
| Study | Neoadjuvant Intervention | Total participants | Toxicity Grading** (n/%) | | | | Individual Adverse Events (AE) | | | | | Incidence of mortality |
|  |  |  | 1-2 | 3 | 4 | 5 | Haematologic | GI | Cardiac | Neurologic | Infectious |  |
|  | Intervention |  |  |  |  |  |  |  |  |  |  |  |
|  | Control |  |  |  |  |  |  |  |  |  |  |  |

| Compliance outcomes of eligible studies | | | | | | | |
| --- | --- | --- | --- | --- | --- | --- | --- |
| Study | Neoadjuvant Intervention | Total participants | Consolidation/Induction Chemotherapy | | | Chemoradiation | |
|  |  |  | Initiated chemotherapy (n/%) | ≥75% of prescribed course (n/%) | ≥90% of prescribed course (n/%) | Initiated chemoradiation (n/%) | ≥4500 cGy of prescribed radiotherapy course (n/%) |
|  | Intervention |  |  |  |  |  |  |
|  | Control |  |  |  |  |  |  |

| Postoperative outcomes of eligible studies | | | | | | | | | | |
| --- | --- | --- | --- | --- | --- | --- | --- | --- | --- | --- |
| Study | Neoadjuvant Intervention | Clavien-Dindo Grade | | | | Individual Postoperative Complications | | | | Incidence of Mortality within 60-day postoperatively |
|  |  | 1-2 | 3 | 4 | 5 | Infection | Bleeding | Thromboembolic | GI-related |  |
|  | Intervention |  |  |  |  |  |  |  |  |  |
|  | Control |  |  |  |  |  |  |  |  |  |

*Race and ethnicity datapoints will be attempted, where available

**Toxicity Grading based on the National Cancer Institute Common Terminology Criteria for Adverse Events version 4.0

## Appendix 5: Detailed Characteristics of the Included Studies

| **Study** | **Treatment Characteristics** | | | **Patient characteristics** | | | **Oncological characteristics** | | **Time interval between 1^st^ and 2^nd^ sequence (weeks)** |
| --- | --- | --- | --- | --- | --- | --- | --- | --- | --- |
|  | **Comparators** | **Treatment Sequence** | **RT Dose (Gy)** | **Gender**  **M:F** | **Age (Median, IQR)** | **ECOG Status** | **Tumour Staging^a^** | **Distance from anal verge, cm (n, %)** |  |
| **cTNT vs iTNT** | | | | | | | | | |
| OPRA Trial (2022)  - Garcia-Aguilar et al. | cTNT | CRT  ↓  8 FOLFOX / 5 CAPOX | 54 | 3:2 | 56 (49-67) | - | Stage II-III | 4.5 (3-6.5) | - |
|  | iTNT | 8 FOLFOX / 5 CAPOX  ↓  CRT | 54 | 3:2 | 59 (51-68) | - | Stage II-III | 4.3 (3-6.3) |  |
| Moyer et al. (2023) | cTNT | CRT  ↓  8 FOLFOX / 5 CAPOX | 25 | 2:3 | 55 (47–62) | - | Stage II-III | <5cm: 38 (56.7)  5-10cm: 20 (29.9)  >10cm: 9 (13.4) | 2-3 |
|  | iTNT | 8 FOLFOX / 5 CAPOX  ↓  SCRT | 50.4 | 1:1 | 58 (52–67) | - |  | <5cm: 18 (40.9)  5-10cm: 17 (38.6)  >10cm: 9 (20.5) |  |
| Bedrikovetski et al. (2023)* | cTNT | CRT  ↓  8 mFOLFOX / 6 CAPOX | 50 | 1:1 | 65 (54-70) | 0: 15 (46%)  1: 17 (54%) | cT2-T4 and/or cN+ | 4.5 (3.0) | 2 |
|  | iTNT | 8 mFOLFOX / 6 CAPOX  ↓  CRT | 50 | 1:1 | 58 (51-67) | 0: 30 (67%)  1: 15 (34%) | cT2-T4 and/or cN+ | 5.4 (3.2) | 2 |
| **cTNT vs. nCRT** | | | | | | | | | |
| RAPIDO (2020)  - Bahadoer et al. | cTNT | SCRT  ↓  6 CAPOX / 9 FOLFOX  ↓  Sx | 25 | 3:2 | 62 (55-68) | 0: 369 (80%)  1: 93 (20%) | cT2-T4 and/or cN+ | <5cm: 103 (22)  5-10cm: 181 (39)  >10cm: 146 (32) | 2 |
|  | nCRT | CRT  ↓  Sx  ↓  6 CAPOX | 50.4 | 3:2 | 62 (55-68) | 1: 365 (81%)  0: 85 (19%) | cT2-T4 and/or cN+ | <5cm: 115 (26)  5-10cm: 153 (34)  >10cm: 151 (34) | - |
| STELLAR (2022)  - Jin et al | cTNT | SCRT  ↓  4 CAPOX  ↓  Sx  ↓  2 CAPOX | 25 | 7:3 | 55 (20-74) | 0: 259 (85.8)  1: 43 (14.2) | cT3-T4 and/or cN+ | <5cm: 147 (48.7)  5-10cm: 153 (50.1)  >10cm: 2 (0.7) | - |
|  | nCRT | CRT  ↓  Sx  ↓  6 CAPOX | 50.4 | 7:3 | 56 (27-70) | 0: 254 (85.5)  1: 43 (14.5) | cT3-T4 and/or cN+ | <5cm: 148 (49.8)  5-10cm: 149 (50.2)  >10cm: 0 (0) |  |
| Li et al. (2017) | cTNT | CRT  ↓  4 FOLFOX  ↓  Sx  ↓  4 FOLFOX | 50.4 | 4:5 | 57 (39-76) | - | cT3-T4 and/or cN+ | <5cm: 14 (35)  5-10cm: 26 (65) | 2-3 |
|  | nCRT | CRT  ↓  Sx  ↓  8 FOLFOX | 50.4 | 2:3 | 58 (34-80) | - | cT3-T4 and/or cN+ | <5cm: 19 (48)  5-10cm: 21 (52) | - |
| Markoniva et al. (2017) | cTNT | SCRT  ↓  4 mFOLFOX  ↓  Sx | 20 | 7:3 | 57.2 (28.3-84.6) | - | cT3-T4 and/or cN+ | <5cm: 20 (29)  5-10cm: 33 (48)  >10cm: 16 (23) | 3 |
|  | nCRT | CRT  ↓  Sx | 50.4 | 3:2 | 56.6 (31-82.5) | - | cT3-T4 and/or cN+ | <5cm: 26 (38)  5-10cm: 34 (49)  >10cm: 9 (13) | - |
| MSKCC Trial  - Marco et al. (2018)  - Garcia-Aguilar et al. (2015) | cTNT | CRT  ↓  4 or 6 mFOLFOX  ↓  Sx | 50 | 3:2 | 4 mFOLFOX: 56 (44-68)  6 mFOLFOX:  59 (50-68) | 4 mFOLFOX: 0: 44 (83)  1: 7 (13)  6 mFOLFOX:  0: 48 (77)  1: 14 (23) | Stage II/III | 4 mFOLFOX:  7.4 (3.0)  6 mFOLFOX: 6.6 (3.3) | 2 |
|  | nCRT | CRT  ↓  Sx | 50 | 3:2 | 61 (48-74) | 0: 38 (95)  1: 2 (5) | Stage II/III | 6.5 (3.2) | - |
| Liang et al. (2019) | cTNT | CRT  ↓  4 FOLFOX  ↓  Sx | 50.4 | 7:3 | 50 (22-70) | - | cT3-T4 and/or cN+ | ≤5cm: 90 (57.7)  6-10cm: 60 (42.3) | - |
|  | nCRT | CRT  ↓  Sx | 50,4 | 3:2 | 55 (24-80) | - | cT3-T4 and/or cN+ |  |  |
| **iTNT vs. nCRT** | | | | | | | | | |
| PRODIGE 23  - Conroy et al. (2020)  - Conroy et al. (2024) | iTNT | 6 mFOLFIRINOX  ↓  CRT  ↓  Sx  ↓  6 FOLFOX / 4 CAP | 50.4 | 3:2 | 61 (53-66) | 0: 178 (78)  1: 51 (22) | cT3-T4 and/or cN+ | <5cm: 87 (38)  5-10cm: 114 (49)  >10cm: 30 (13) | 2-4 |
|  | nCRT | CRT  ↓  Sx  ↓  12 FOLFOX / 8 CAP | 50.4 | 3:2 | 62 (55-66) | 0: 182 (81)  1: 44 (19) | cT3-T4 and/or cN+ | <5cm: 83 (36)  5-10cm: 118 (51)  >10cm: 29 (13) |  |
| GCR 3  - Fernándes-Martos et al. (2010)  - Fernándes-Martos et al. (2015) | iTNT | 4 CAPOX  ↓  CRT  ↓  Sx | 50.4 | 7:3 | 60 (38-76) | 0: 33 (59)  1: 22 (39) | cT3-T4 and/or cN+ | - | 2 |
|  | nCRT | CRT  ↓  Sx  ↓  4 CAPOX | 50.4 | 2:1 | 62 (42-75) | 0: 36 (69)  1: 15 (29) | cT3-T4 and/or cN+ | - |  |
| FORWAC (2018)  - Deng et al. (2016)  - Deng et al. (2018) | iTNT | 4-6 mFOLFOX  ↓  CRT  ↓  Sx | 50.4 | 7:3 | 52.2 (40-64) | - | Stage II/III | <5cm: 83 (50.3)  5-10cm: 75 (45.5)  >10cm: 7 (4.2) | - |
|  | nCRT | CRT  ↓  Sx  ↓  7 5-FU | 50.4 | 3:2 | 54 (42-66) | - | Stage II/III | <5cm: 90 (54.5)  5-10cm: 70 (42.4)  >10cm: 5 (3.0) |  |
| PROSPECT (2023)  - Schrag et al. | iTNT | 6 mFOLFOX  ↓  CRT  ↓  Sx | 50.4 | 3:2 | 57 (19-91) | 0/1: 582 (99.5) | cT3-T4 and/or cN+ | <5cm: 83 (14.2)  5-10cm: 375 (64.1)  >10cm: 127 (21.7) | 2-3 |
|  | nCRT | CRT  ↓  Sx | 50.4 | 3:2 | 57 (25-84) | 0/1: 540 (99.4) | cT3-T4 and/or cN+ | <5cm: 90 (16.6)  5-10cm: 344 (63.4)  >10cm: 109 (20.1) | - |
| KIR (2020)  - Aurelie Garant et al. | iTNT | 6 FOLFOX  ↓  Sx  ↓  6 FOLFOX | 26 | 7:3 | 62 (24-85) | ≥2: 120 | cT3-T4 and/or cN+ | <5cm: 68 (57)  5-10cm: 49 (41)  >10cm: 3 (2) | 4 |
|  | nCRT | CRT  ↓  Sx  ↓  12 FOLFOX | 26 | 3:2 | 63 (37-80) | ≥2: 60 | cT3-T4 and/or cN+ | <5cm: 32 (53)  5-10cm: 25 (44)  >10cm: 2 (3) | - |
| Cercek et al. (2018) | iTNT | 8 mFOLFOX / 5 CAPOX  ↓  CRT  ↓  Sx | - | 3:2 | 56.7 (43.8-69.6) | - | cT3-T4 and/or cN+ | <5cm: 102 (33.1)  5-10cm: 143 (46.4)  >10cm: 63 (20.5) | 2-3 |
|  | nCRT | CRT  ↓  Sx  ↓ | - | 3:2 | 56.7 (43.8-69.6) | - | cT3-T4 and/or cN+ | <5cm: 98 (30.6)  5-10cm: 175 (54.7)  >10cm: 47 (14.7) | - |
| Quezada-Diaz et al. (2019) | iTNT | 8 mFOLFOX / 5 CAPOX / FLOX  ↓  CRT  ↓  Sx | 50.4 | 1:1 | 53.0 (26–72) | - | Stage I-III | 8 (1.-10.0) | 2-3 |
|  | nCRT | CRT  ↓  Sx  ↓  FOLFOX / CAPOX / FLOX | 50.4 | 3:2 | 54.0 (34–78) | - | Stage I-III | 6.75 (1.0-10.0) | - |
| Bhatti et al. (2015) | iTNT | 4 CAPOX  ↓  CRT  ↓  Sx | 50.4 | 3:2 | 41 (15-72) | - | cT3-T4 and/or cN+ | <5cm: 65 (69.9)  >5cm: 28 (30.1) | 3 |
|  | nCRT | CRT  ↓  Sx | 50.4 | 3:2 | 41 (15-72) | - | cT3-T4 and/or cN+ | <5cm: 47 (77)  >5cm: 14 (27) | - |
| Van Zoggel et al. (2018) | iTNT | 4 FOLFOX  ↓  CRT  ↓  Sx | 30.4 | 8:2 | 64 (33-76) | - | Stage II-III | - | 2-3 |
|  | nCRT | CRT  ↓  Sx | 30.4 | 3:2 | 65 (30-84) | - | Stage II-III | - | - |

^a^ Staging according to American Joint Committee on Cancer (AJCC)

* Individual data soured from author correspondence

RT, Radiation Therapy; ECOG, Eastern Collaborative Oncology Group; cTNT, consolidation total neoadjuvant therapy; iTNT, induction total neoadjuvant therapy; nCRT, standard neoadjuvant chemoradiation

## Appendix 6: Risk of Bias analysis

*Risk of Bias 2.0 (ROB 2.0) assessment for randomised controlled trials*


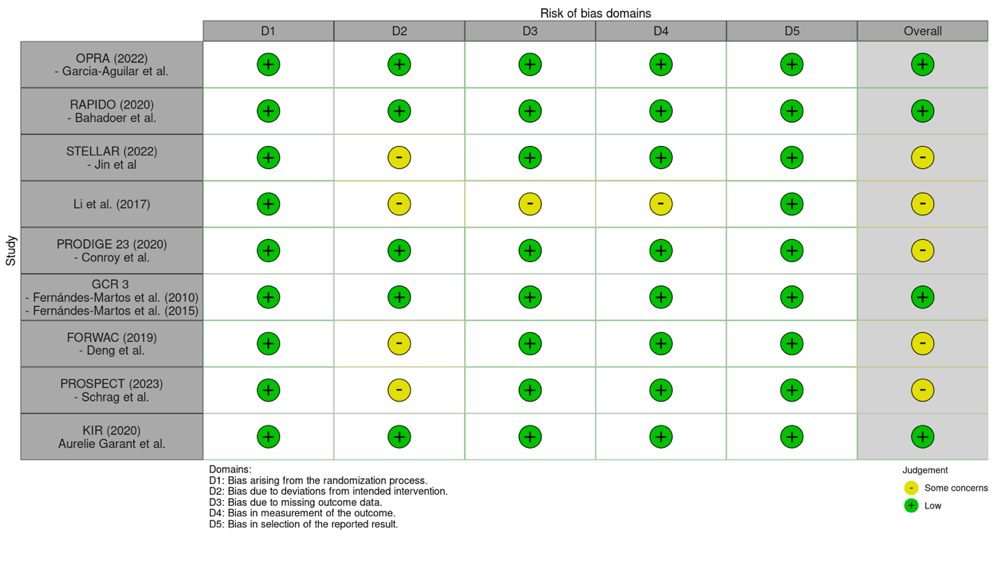


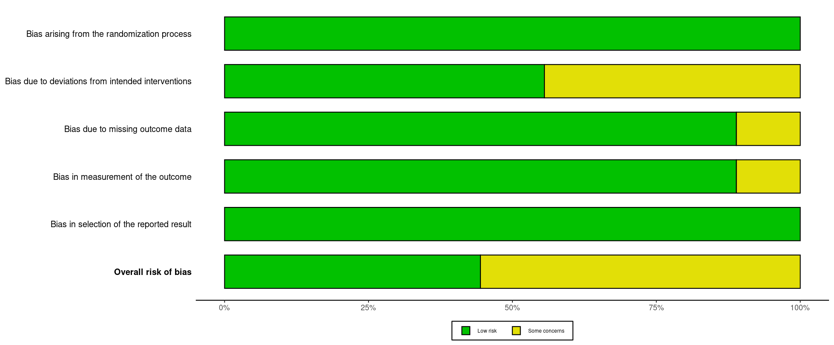


*Risk Of Bias In Non-randomised Studies - of Interventions (ROBINS-I) assessment for non-randomised studies*


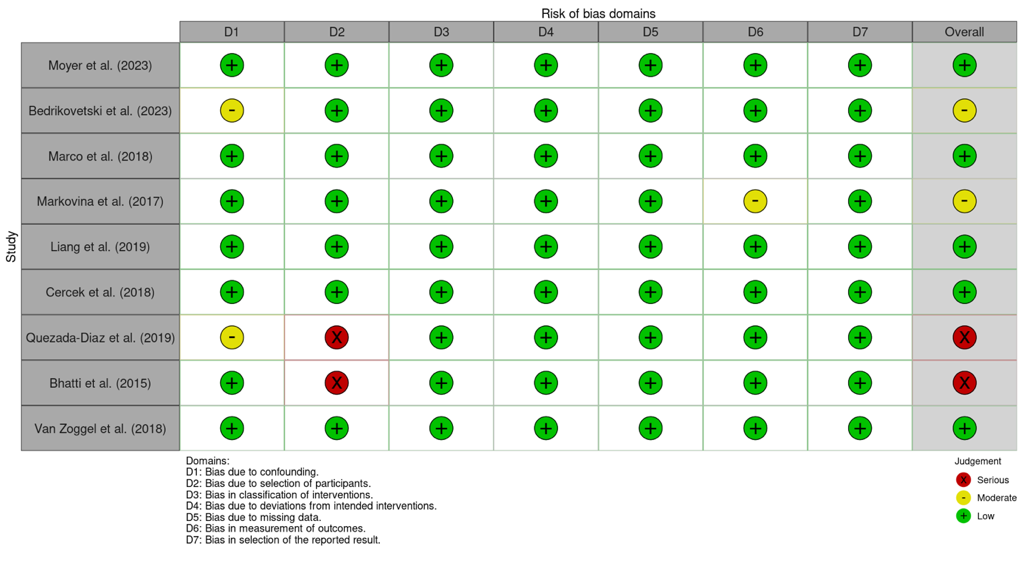


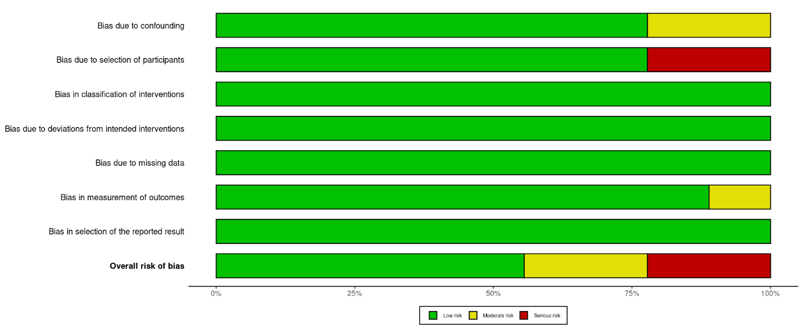


## Appendix 7: Geometrical network maps for all outcomes

| **Toxicity outcomes** | |
| --- | --- |
| 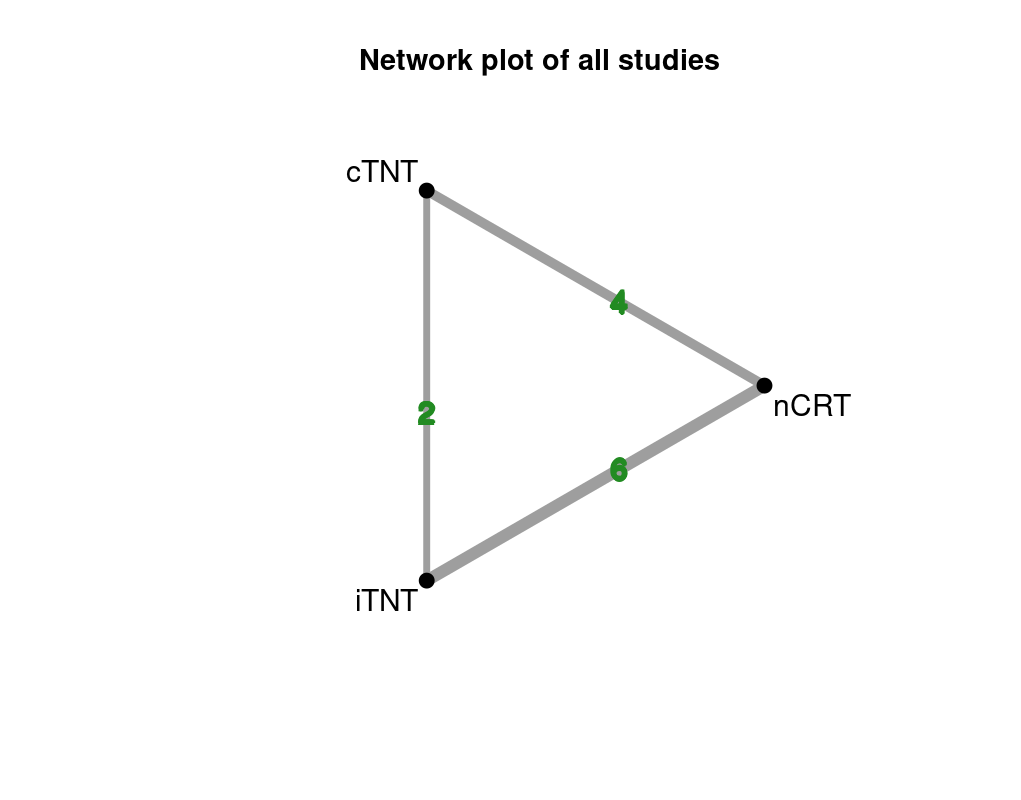 | 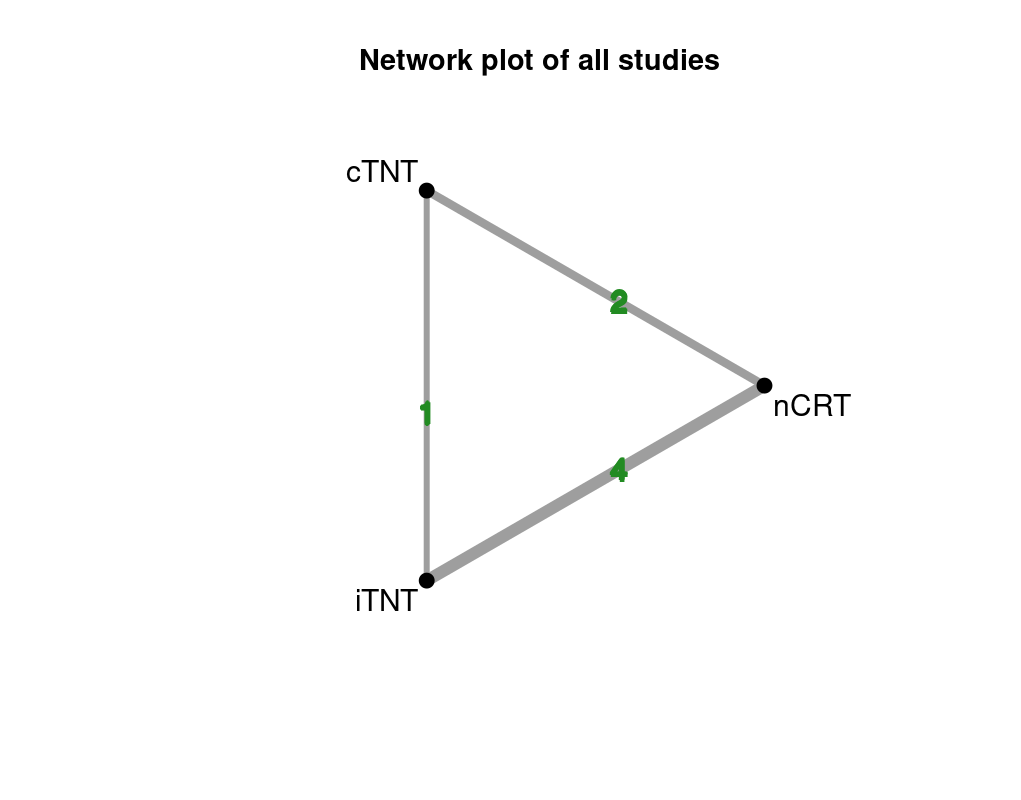 |
| Overall Grade III and above treatment-related adverse events | Rate of treatment-related mortality |
| Individual rate of treatment-related adverse events | |
| 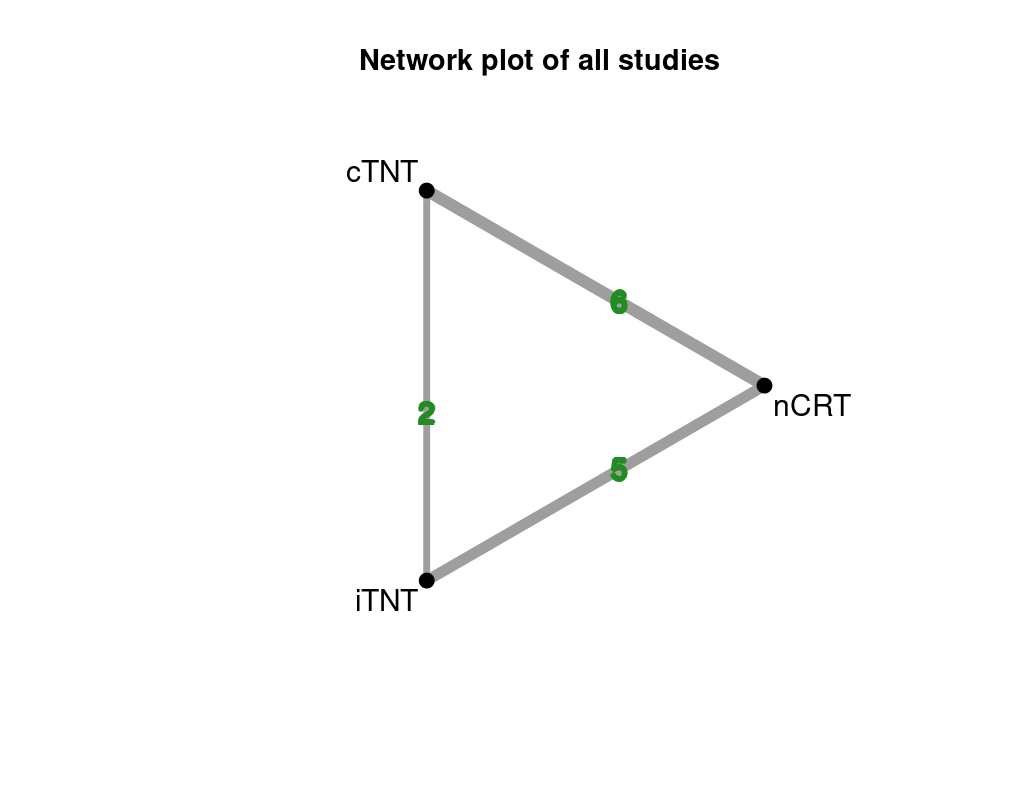 | 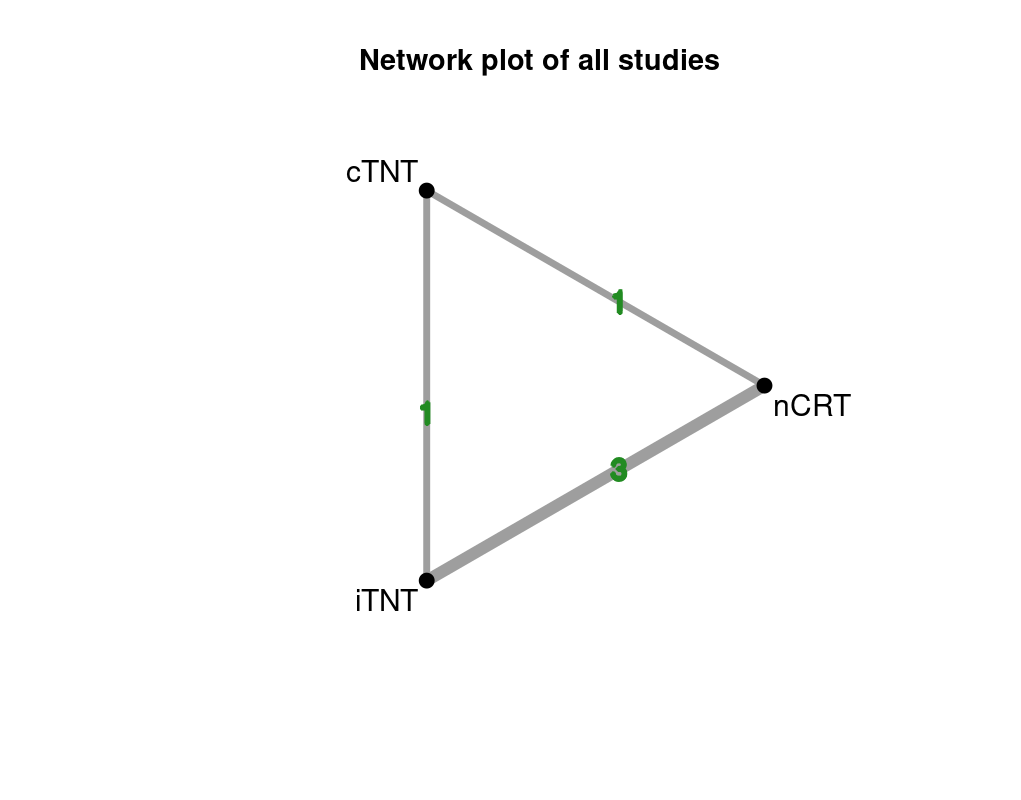 |
| Diarrhoea | Mucositis |
| 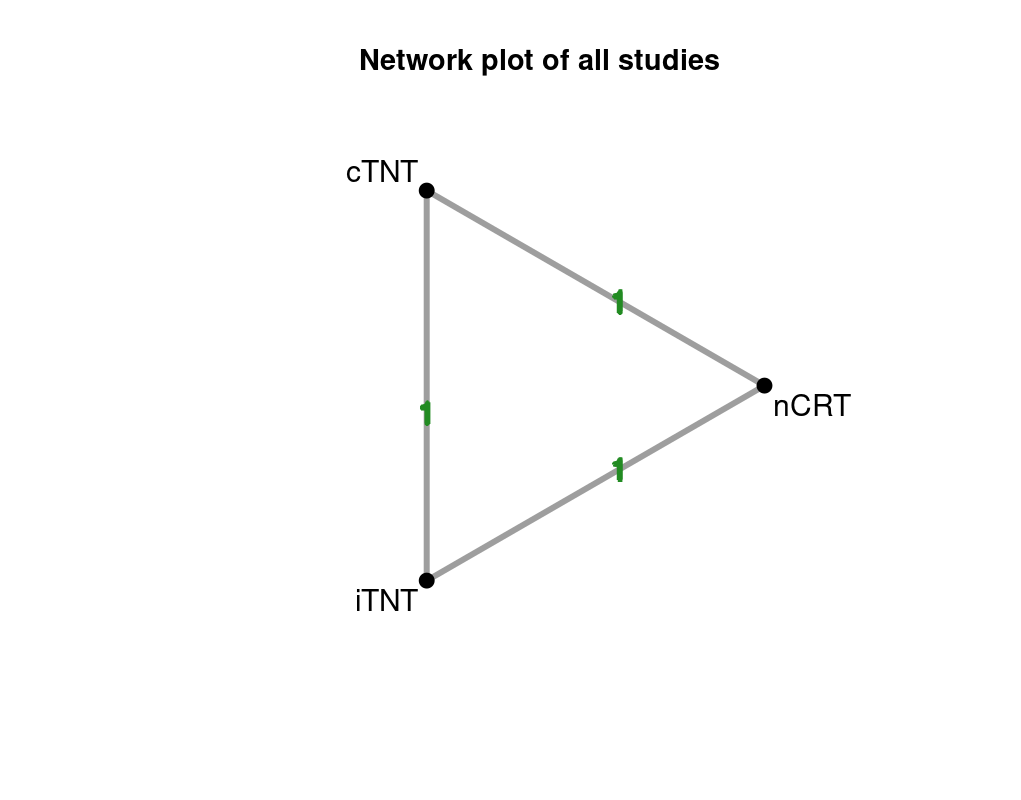 | 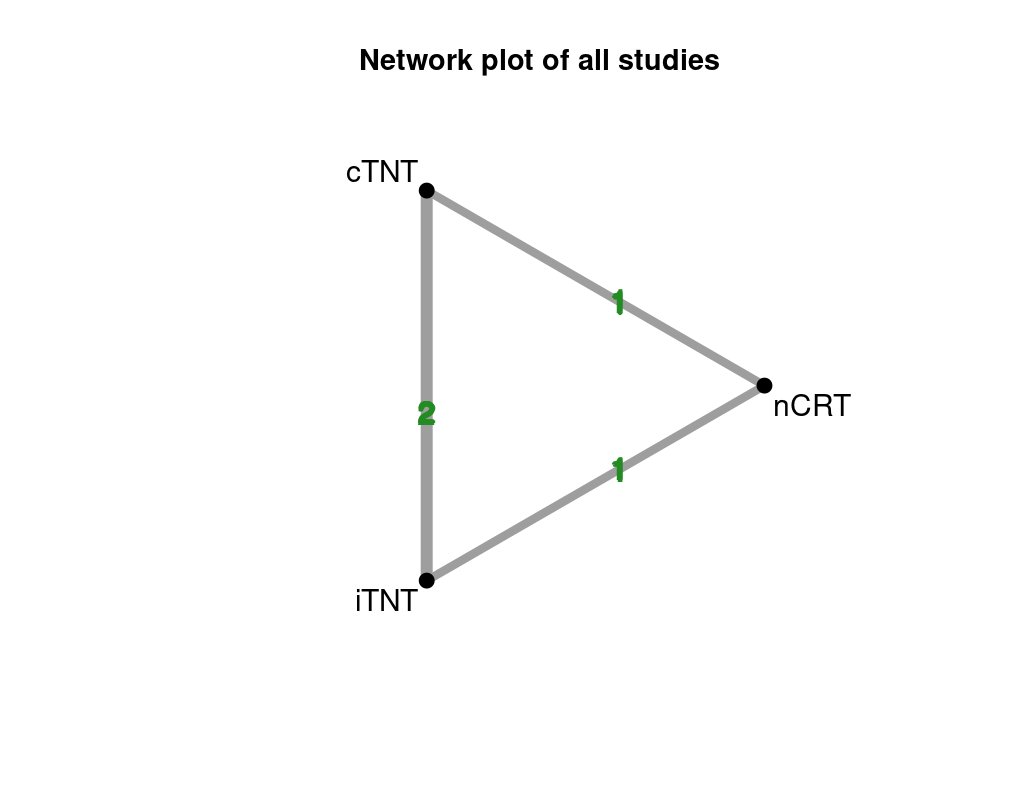 |
| Oesophagitis | Enterocolitis |
| 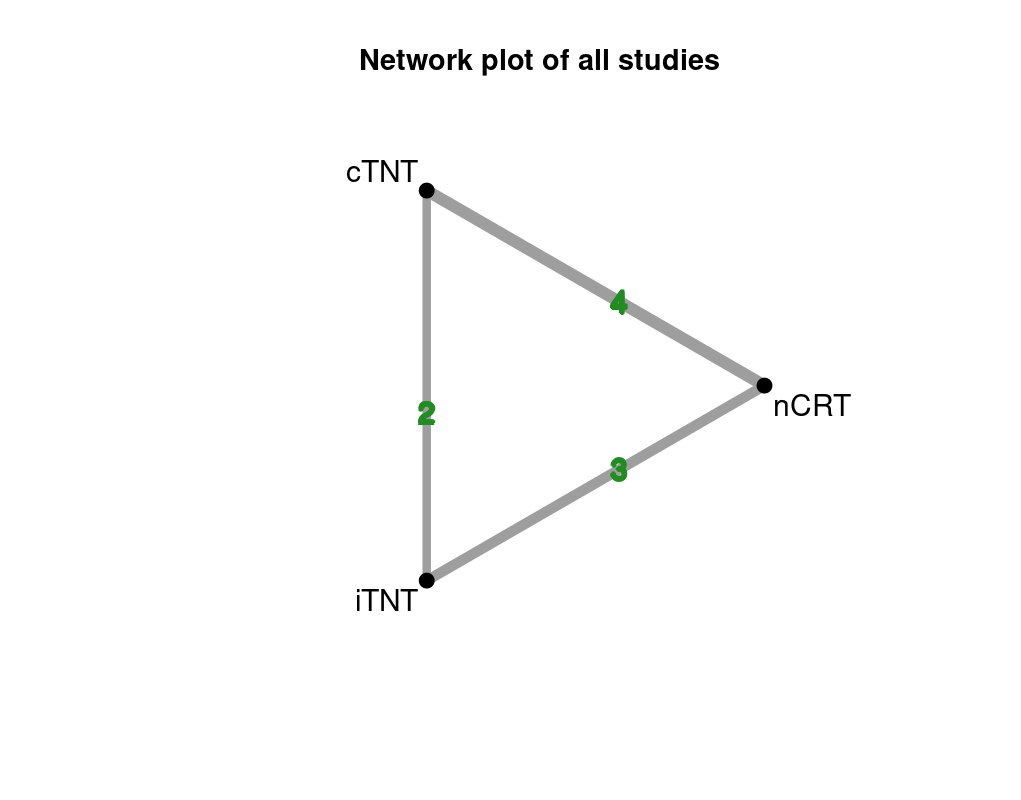 | 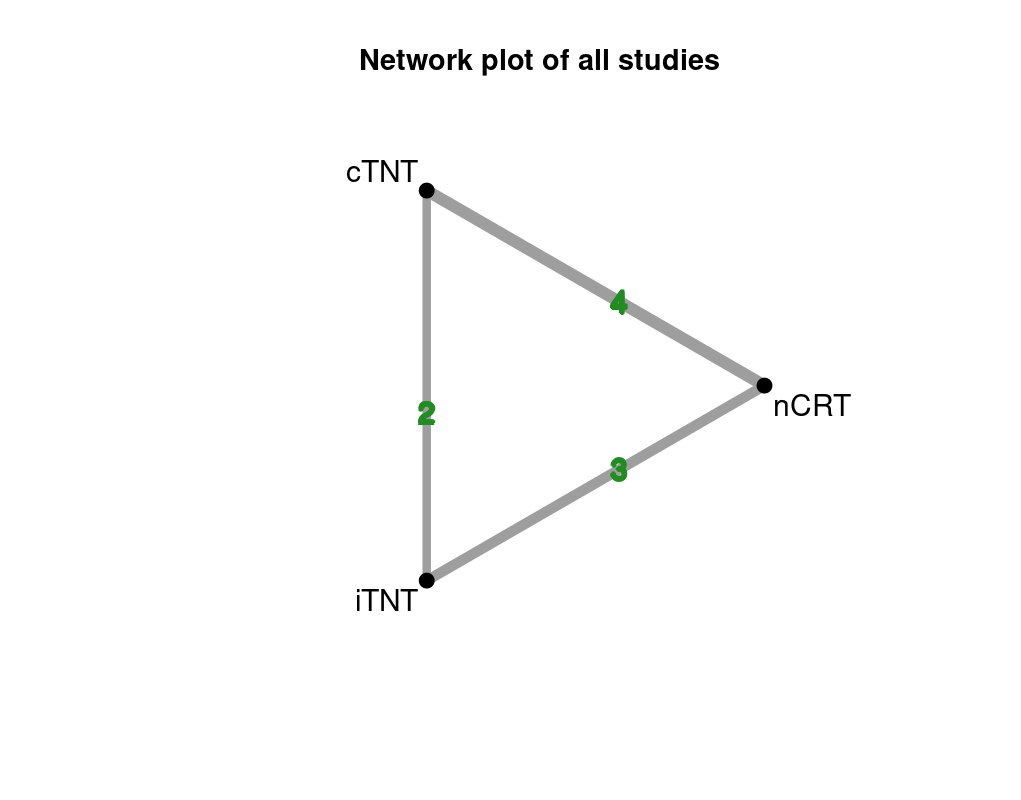 |
| Vomiting | Nausea without vomiting |
| 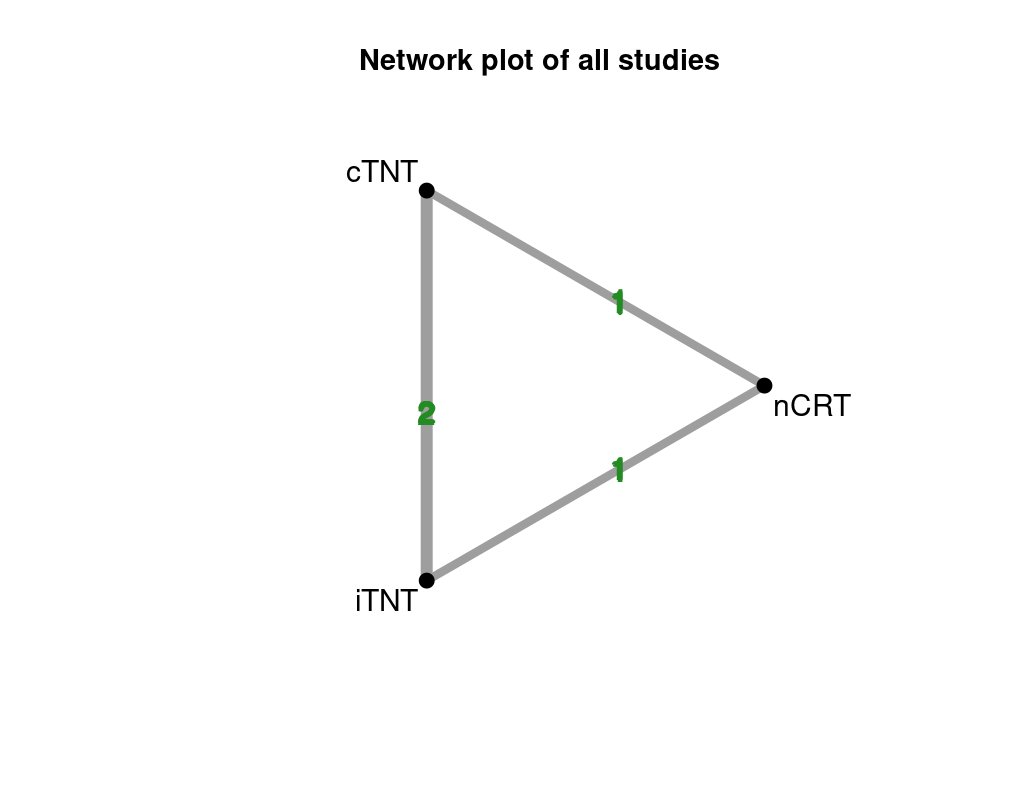 | 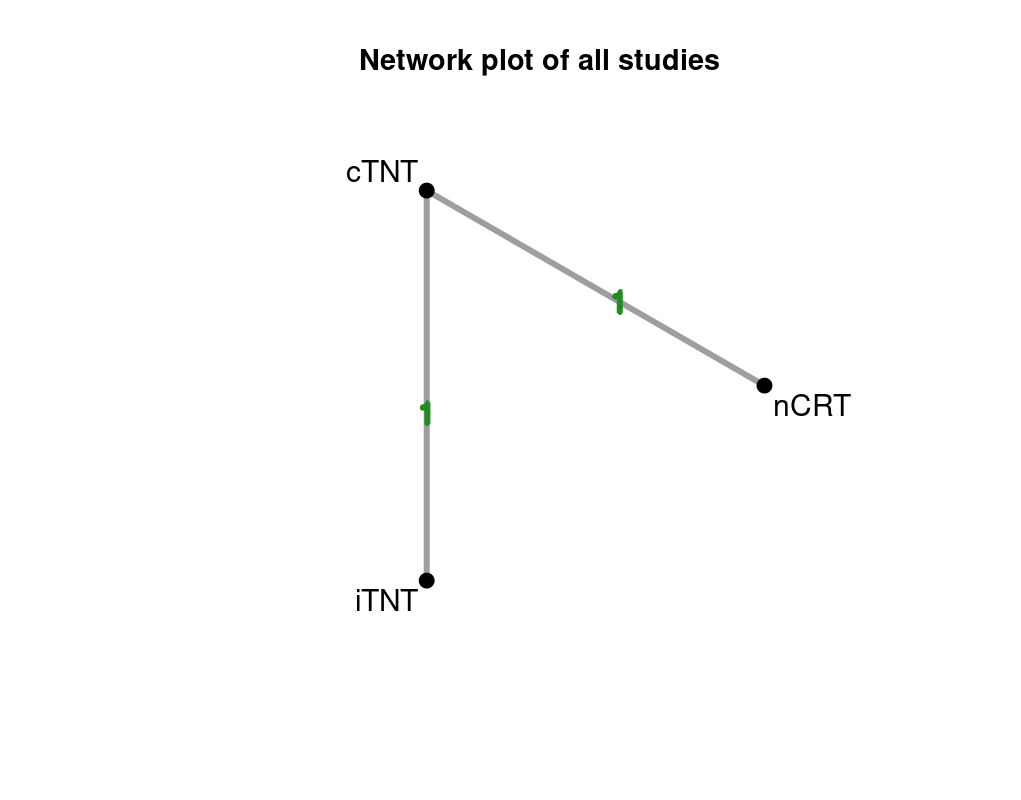 |
| Bowel obstruction | Pancreatitis |
| 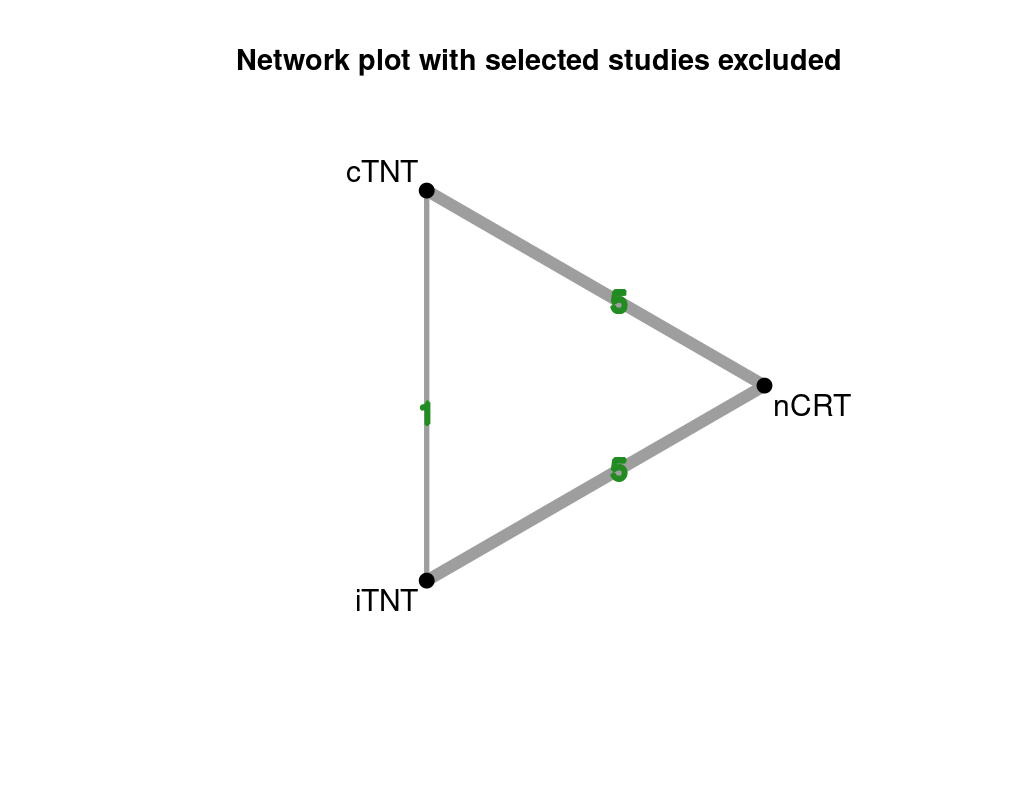 | 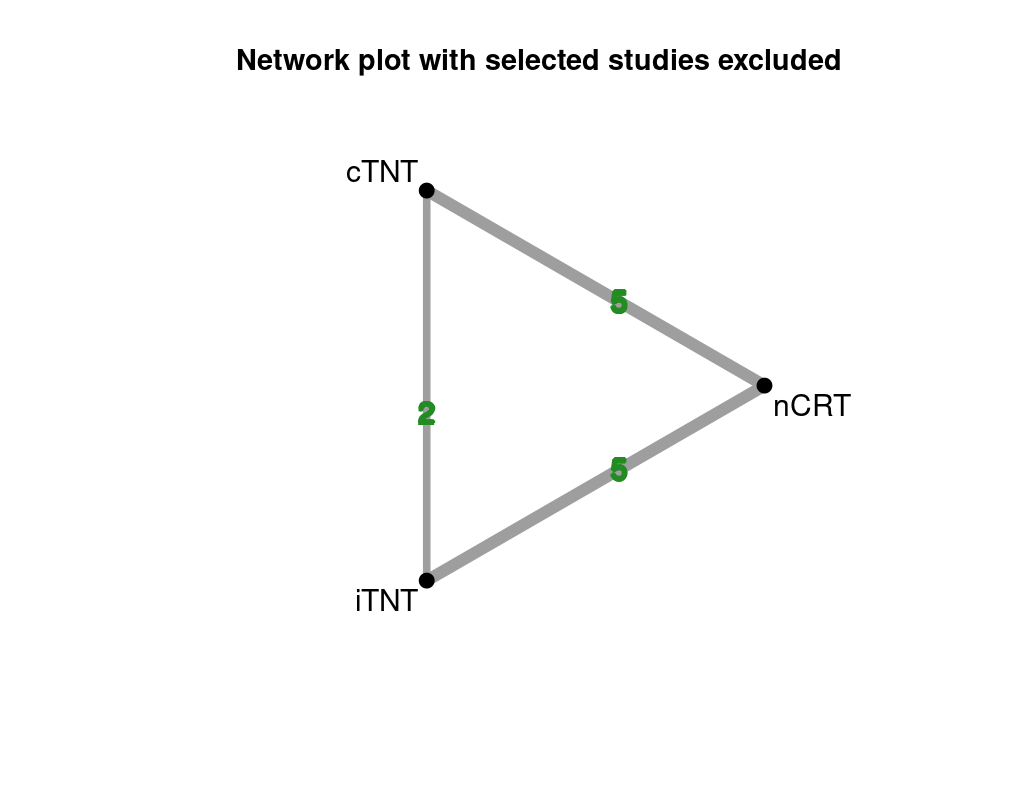 |
| Proctitis | Rectal bleeding |
| 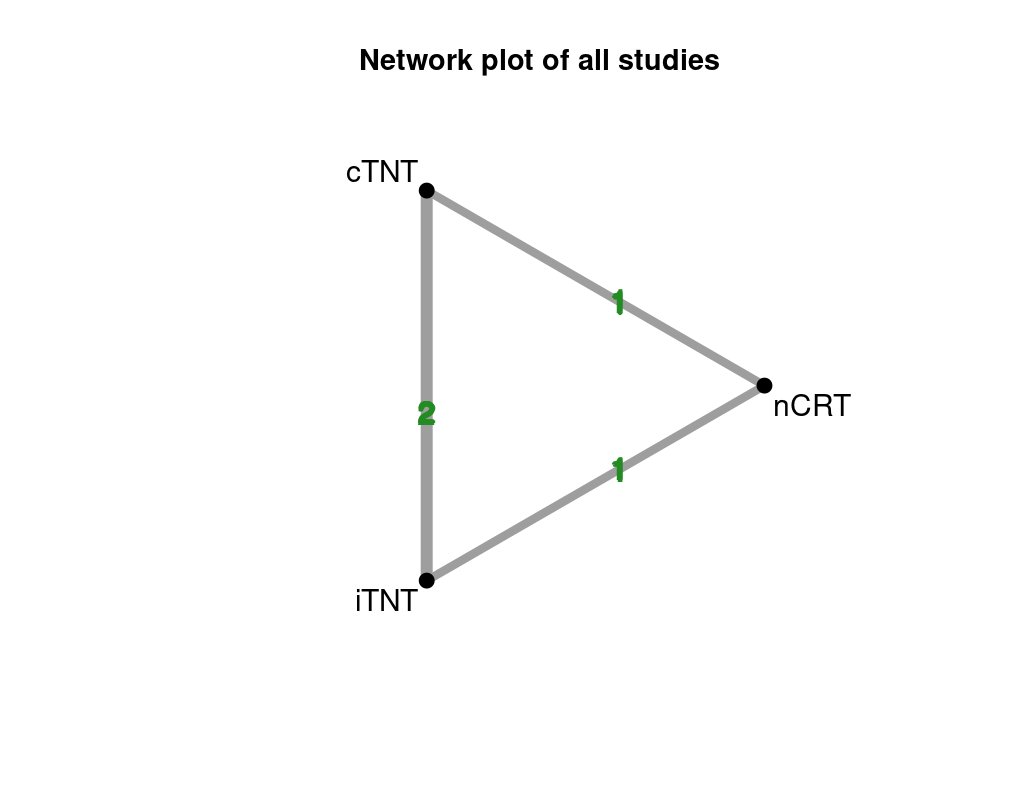 | 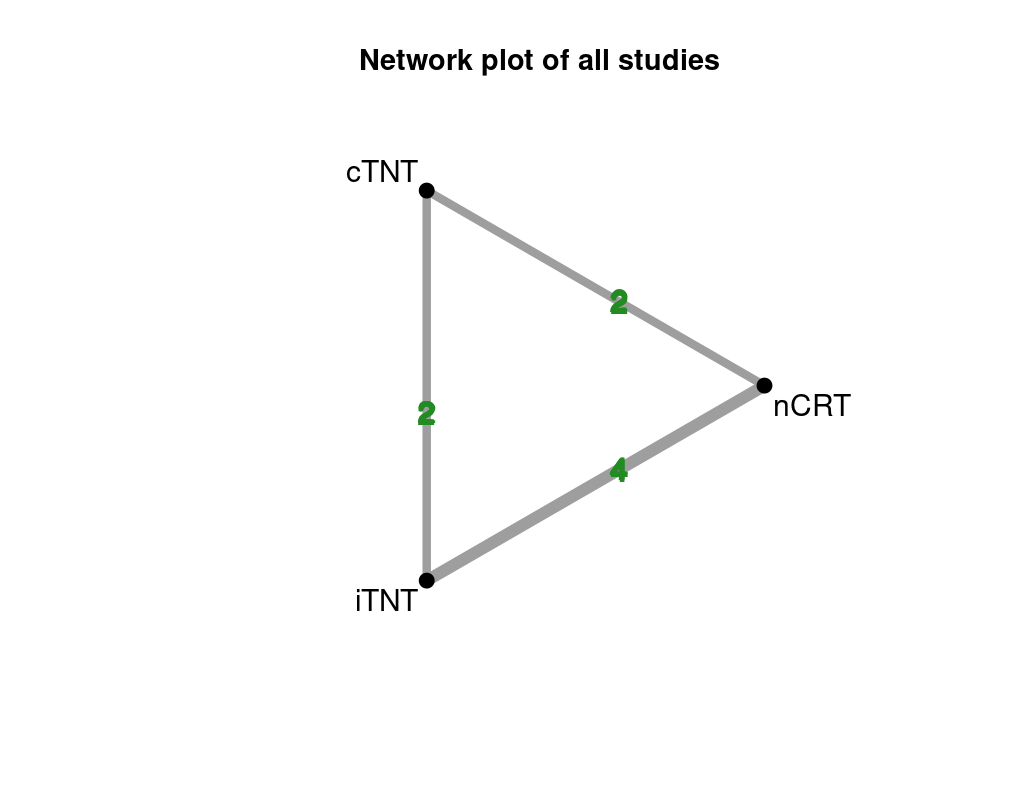 |
| Dizziness | Dysarthria |
| 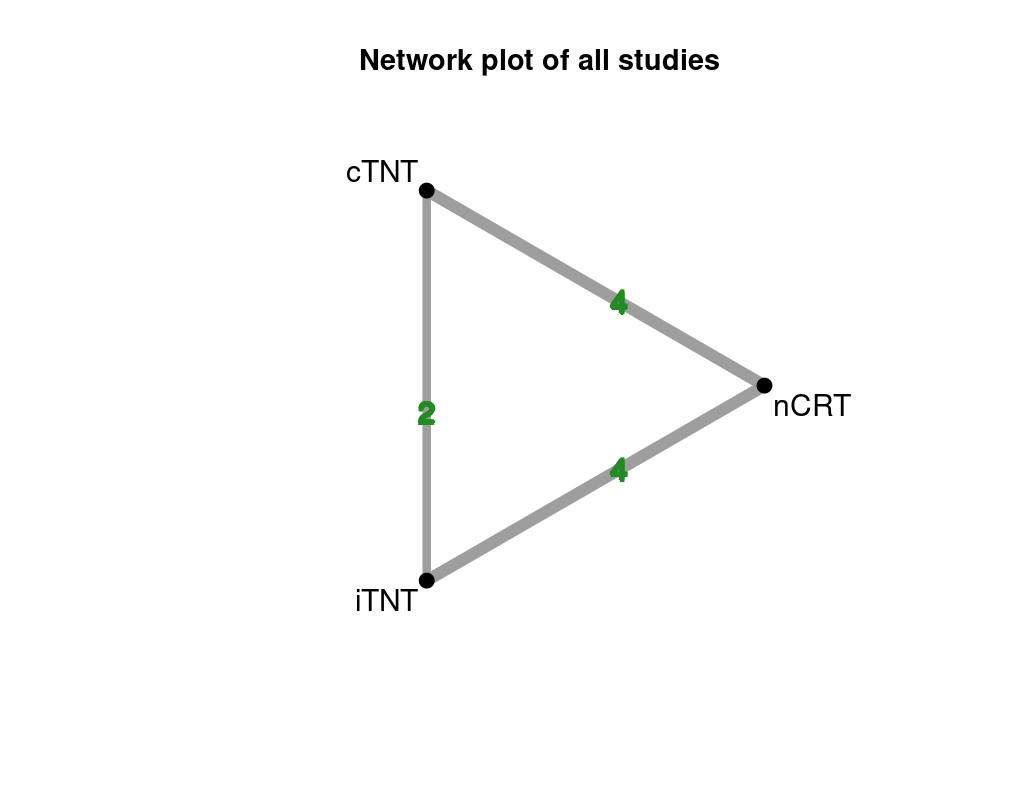 | 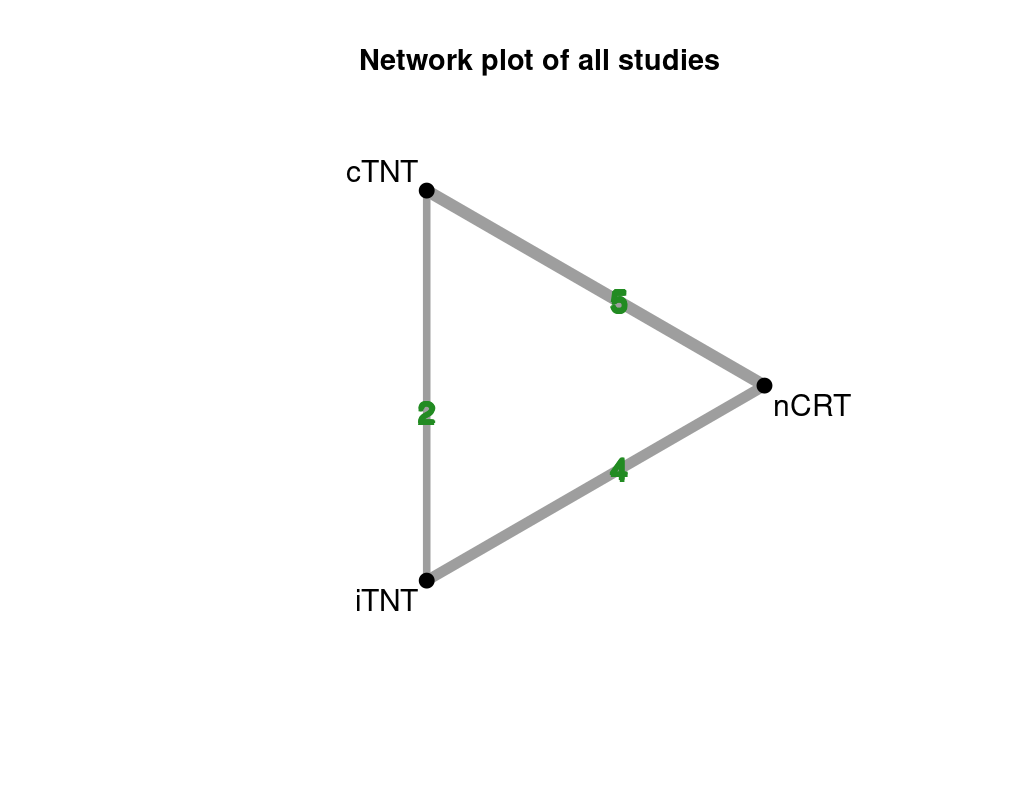 |
| Hand-foot syndrome | Neuropathy |
| 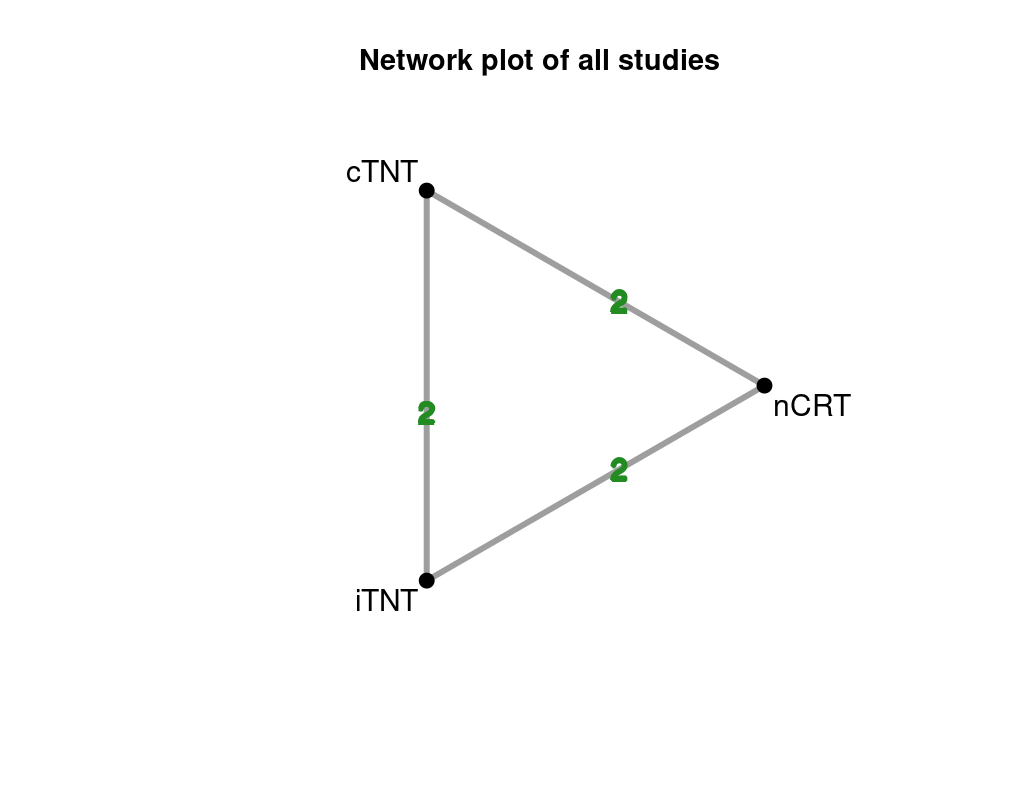 | 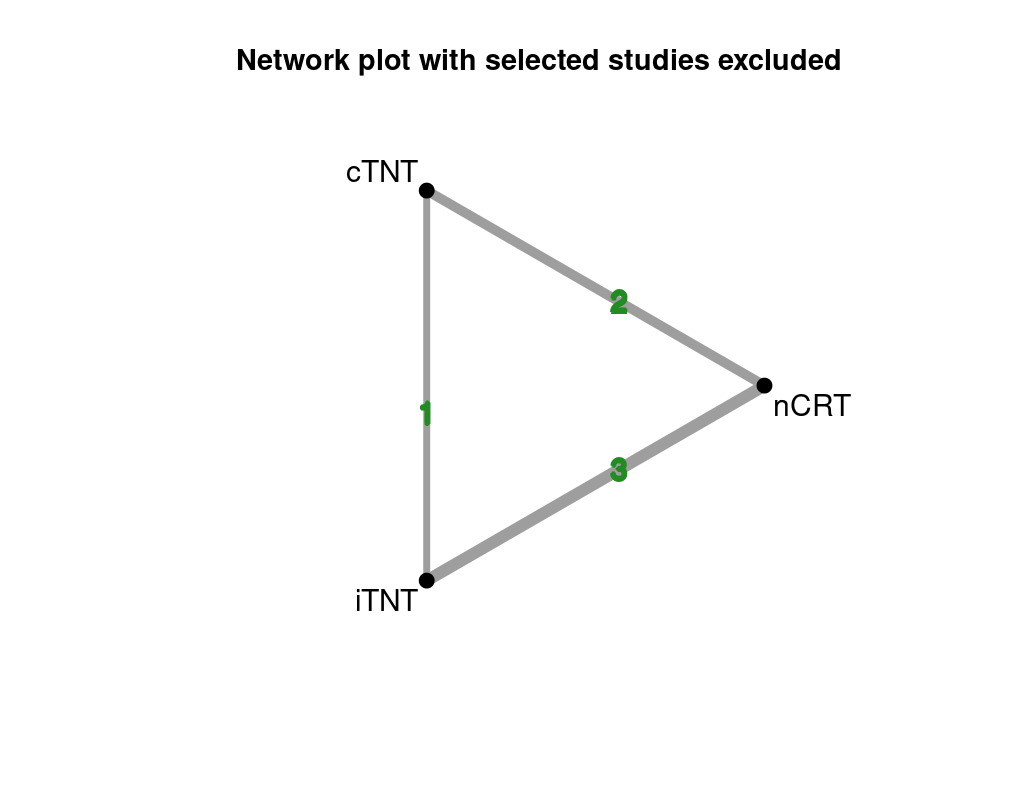 |
| Syncope | Musculoskeletal weakness |
| 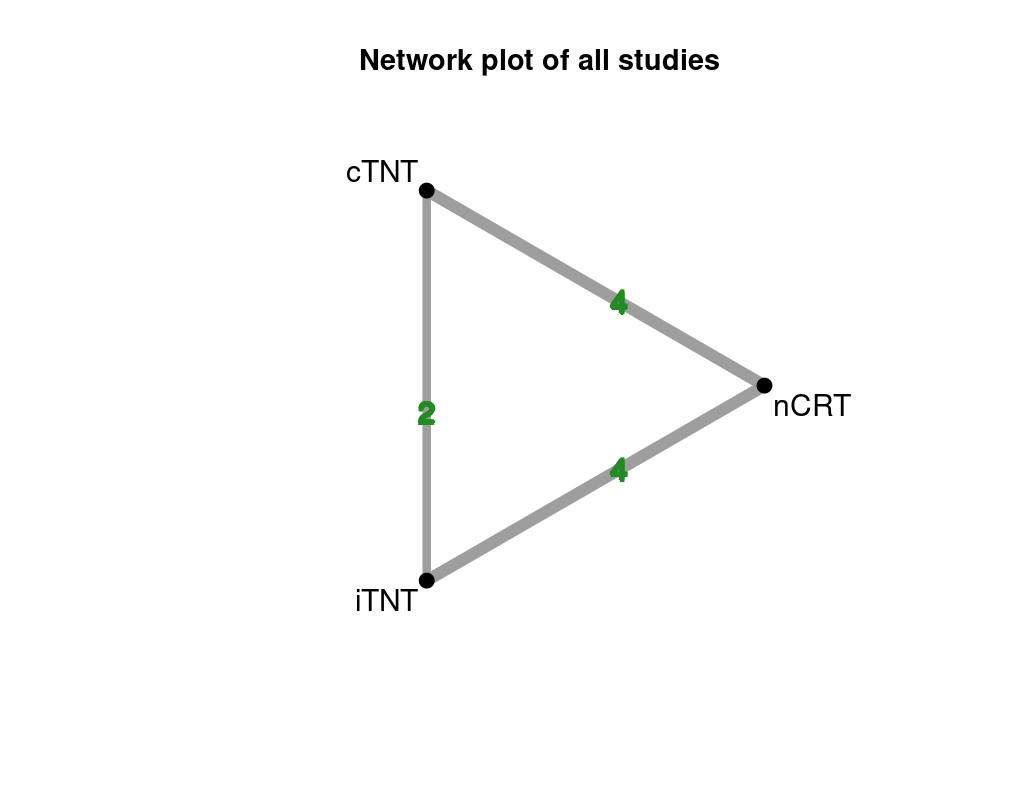 | 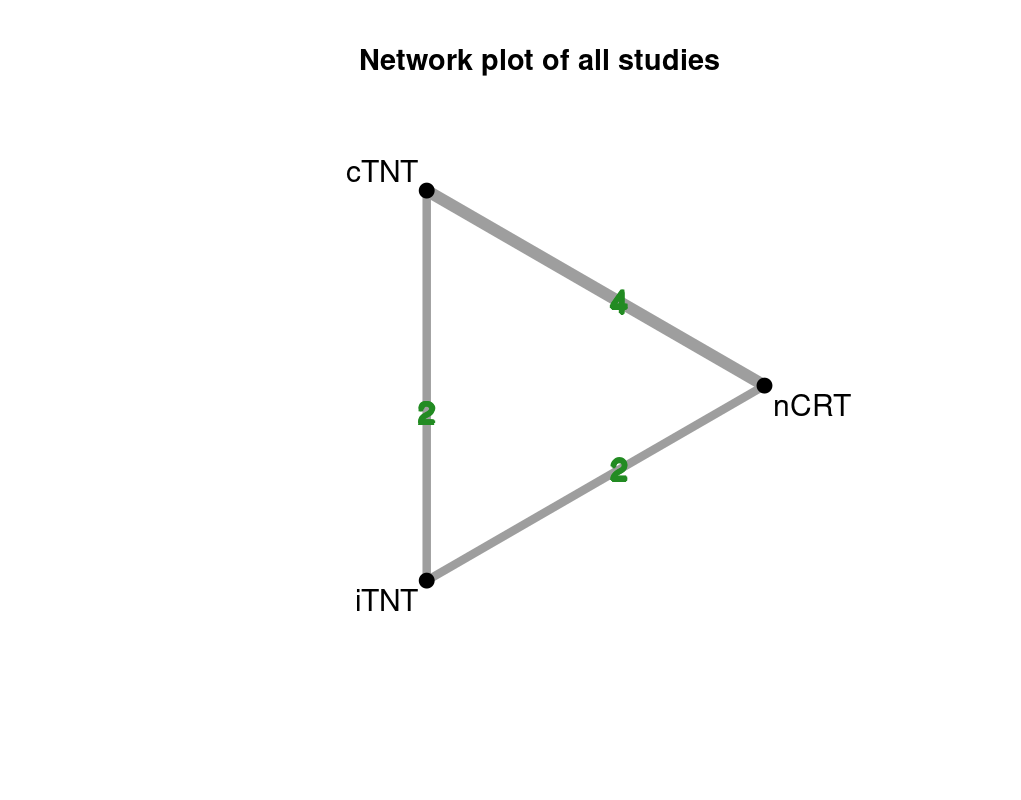 |
| Arrythmias | Venous thromboembolism |
| 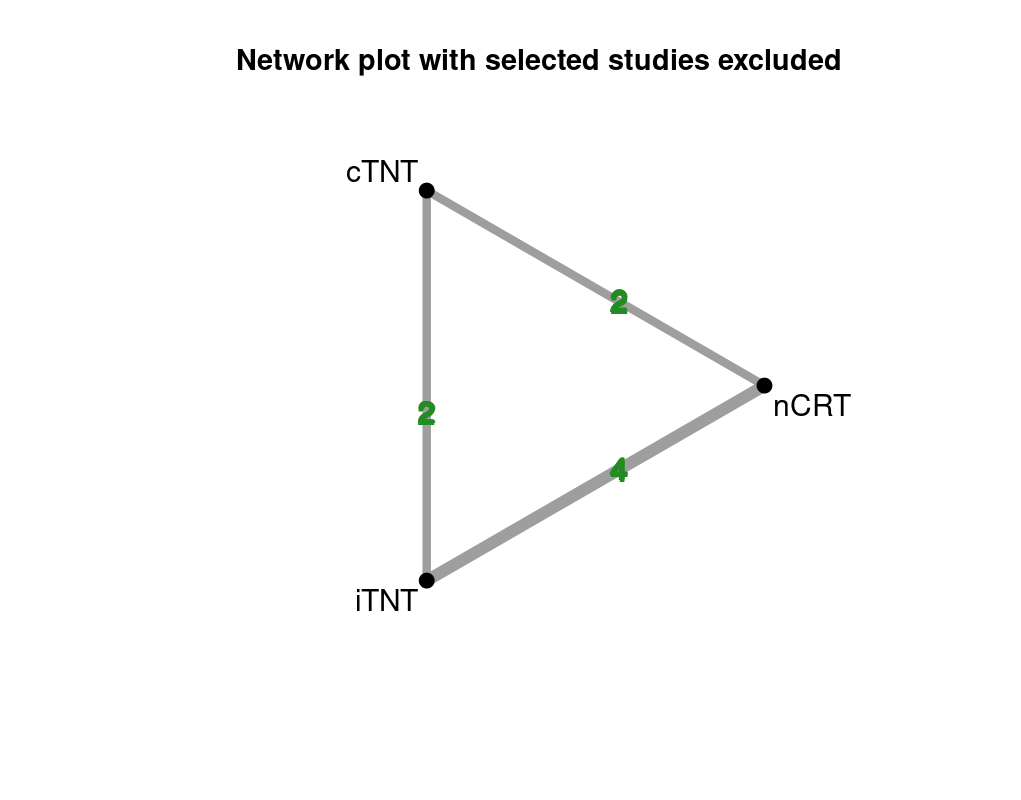 | 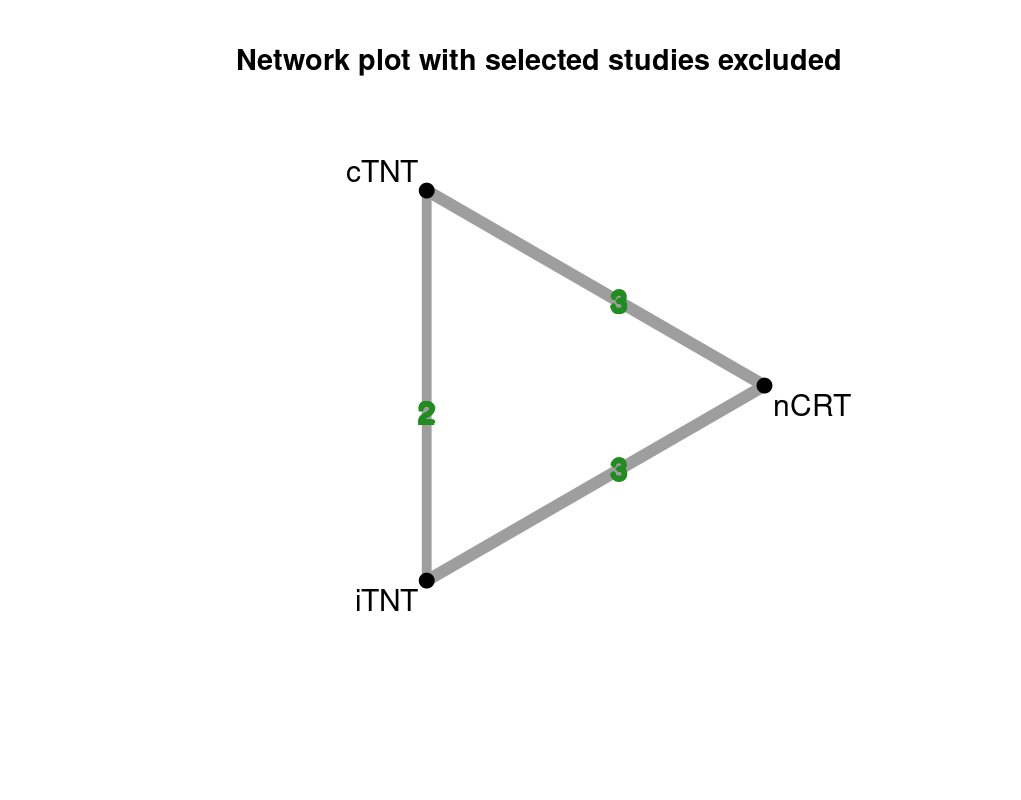 |
| Pneumonia | Urinary tract infection |
| 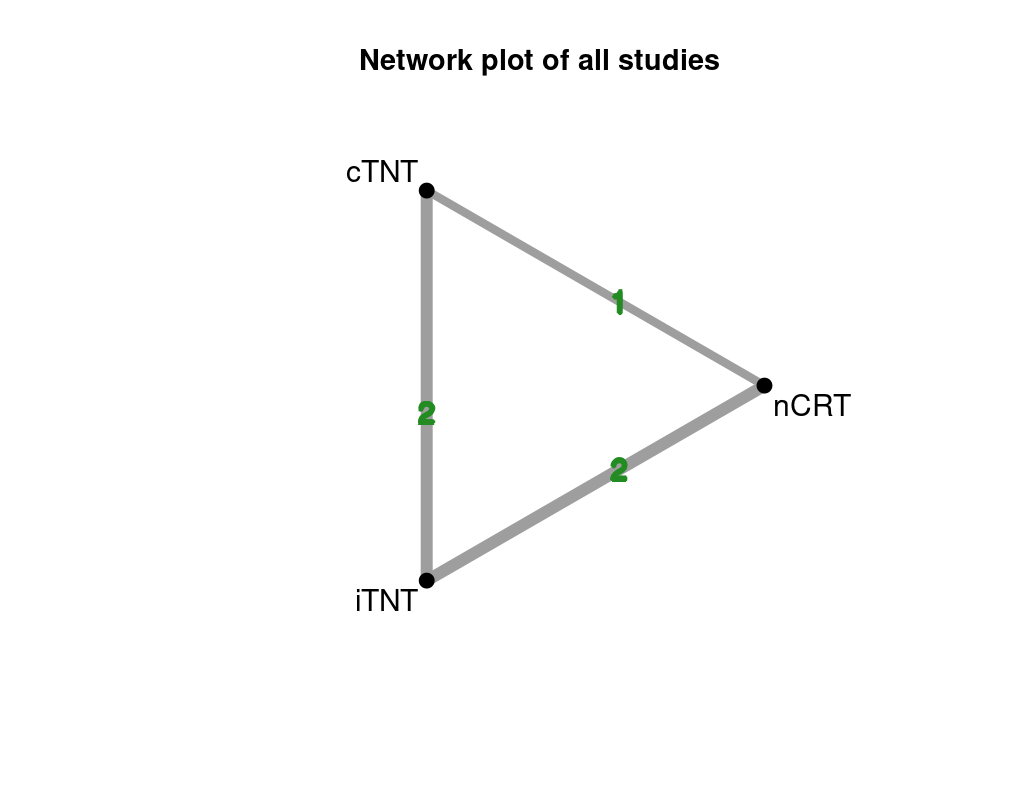 | 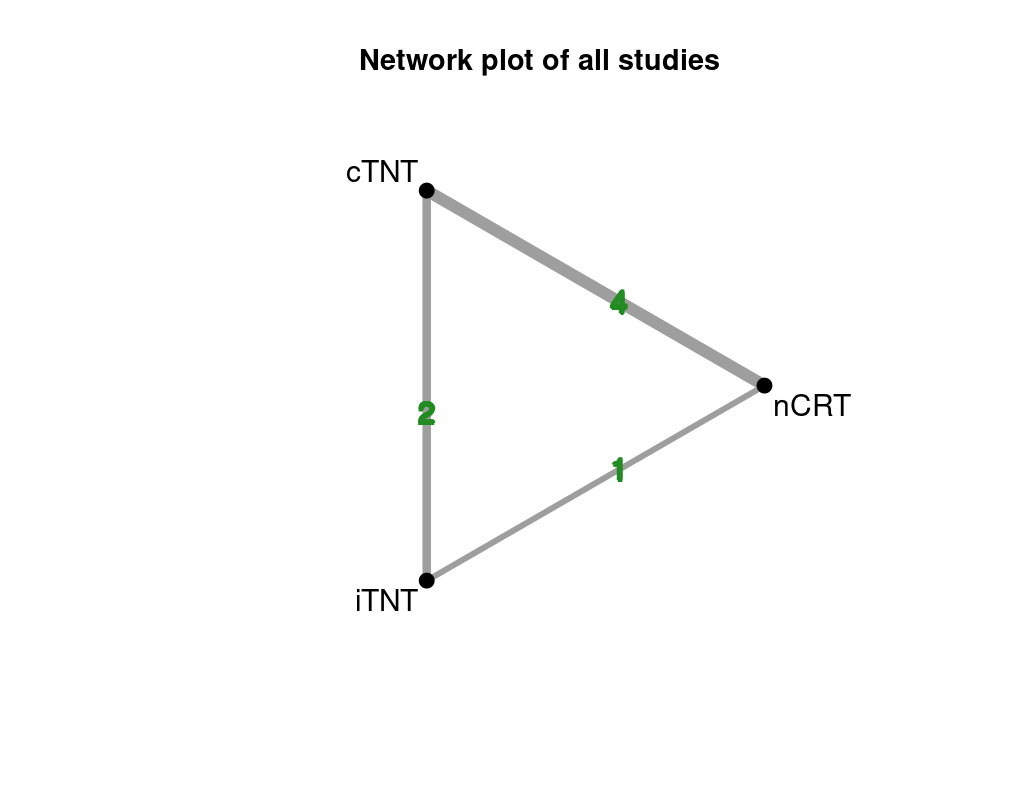 |
| Sepsis | Radiation dermatitis |
| 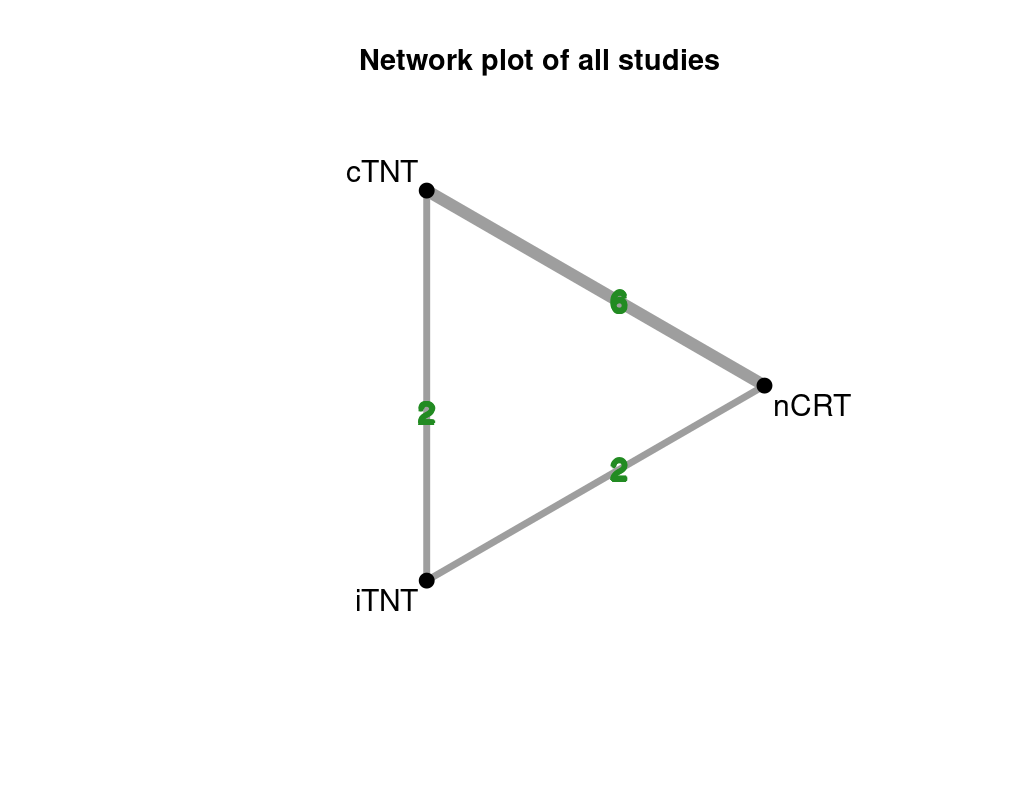 | 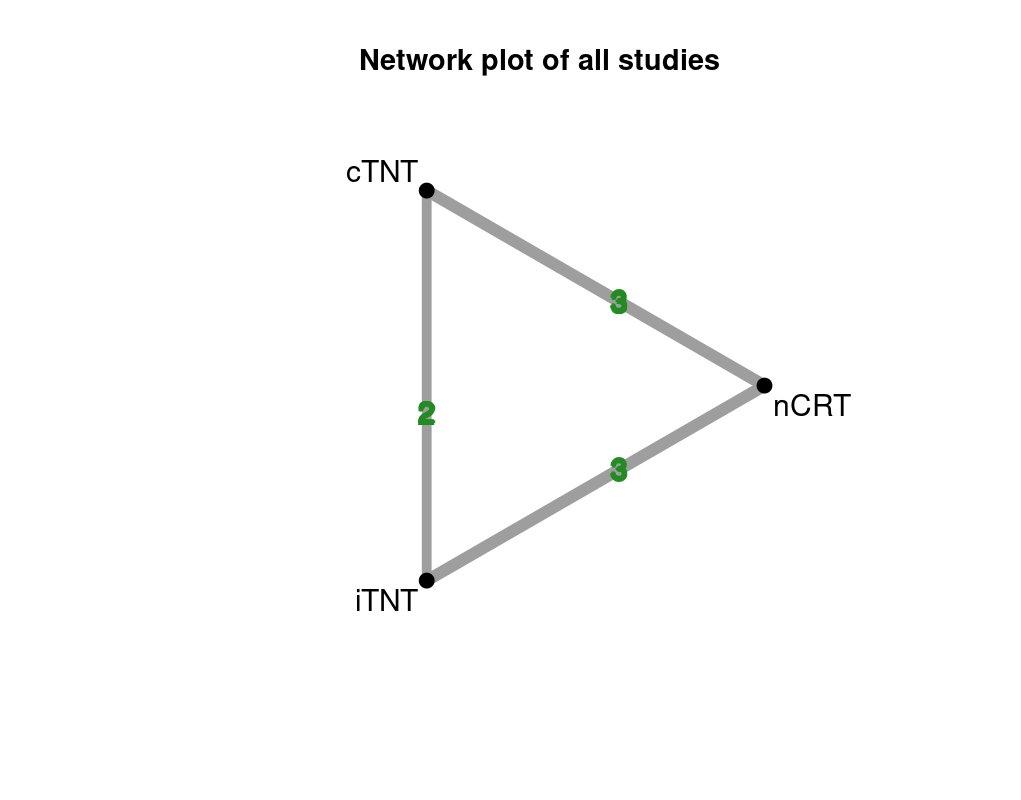 |
| Renal and electrolyte imbalance | Febrile neutropaenia |
| 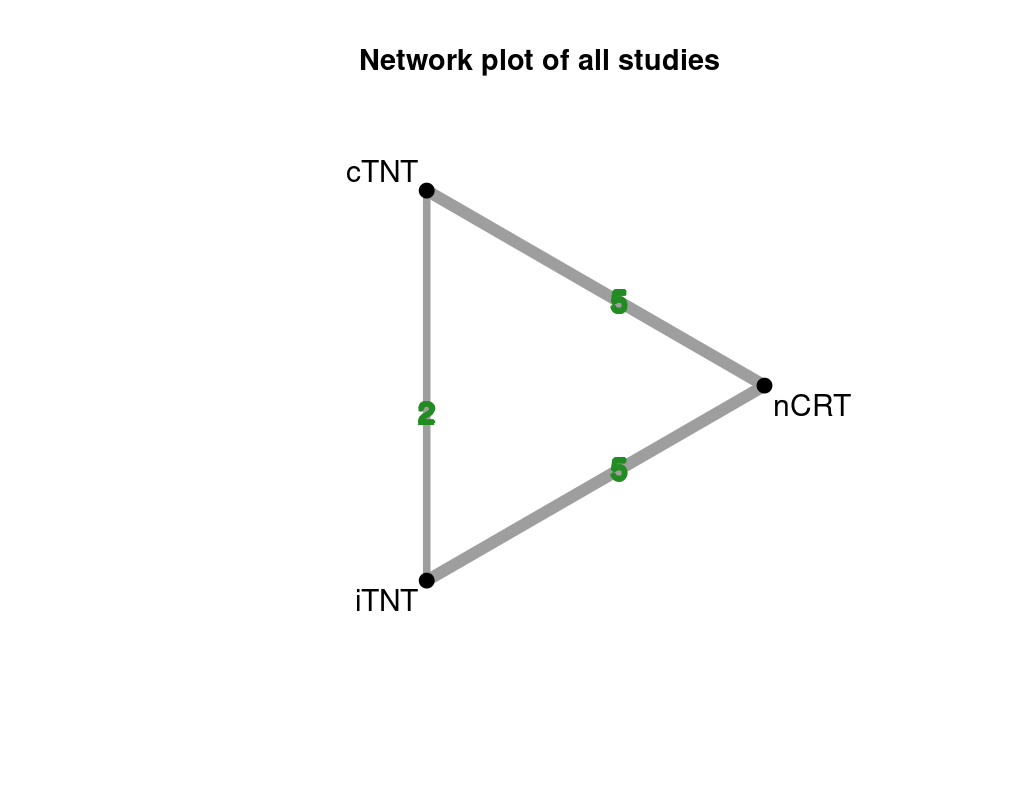 | 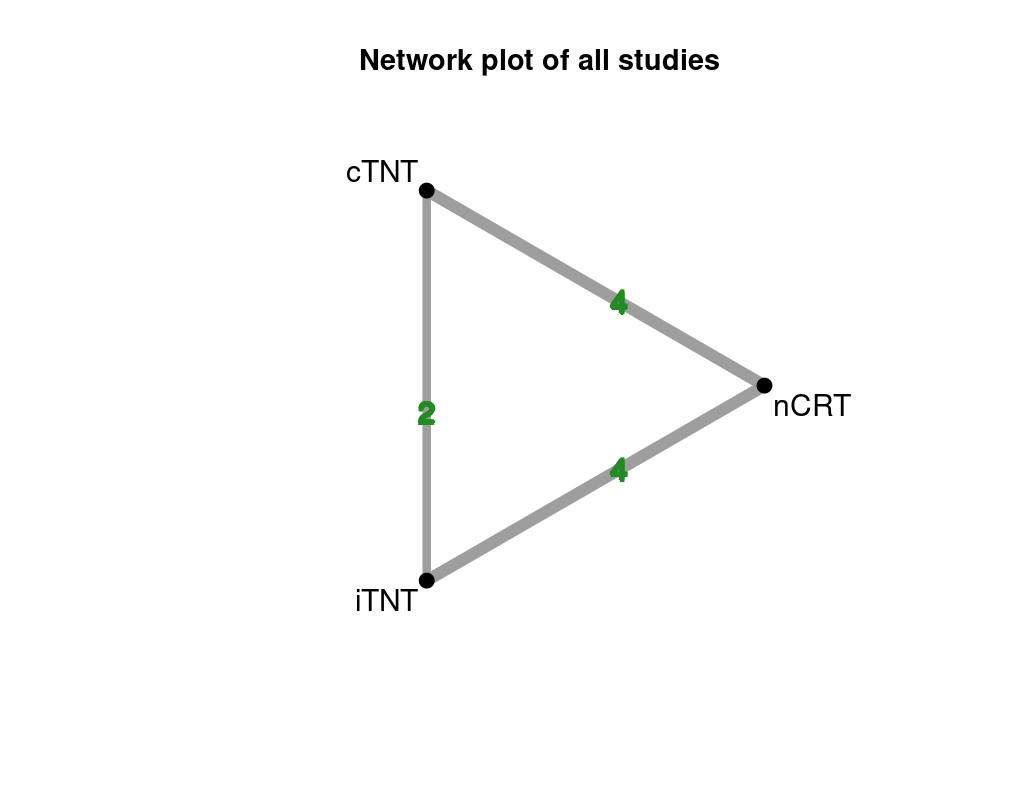 |
| Neutropaenia | Lymphopenia |
| 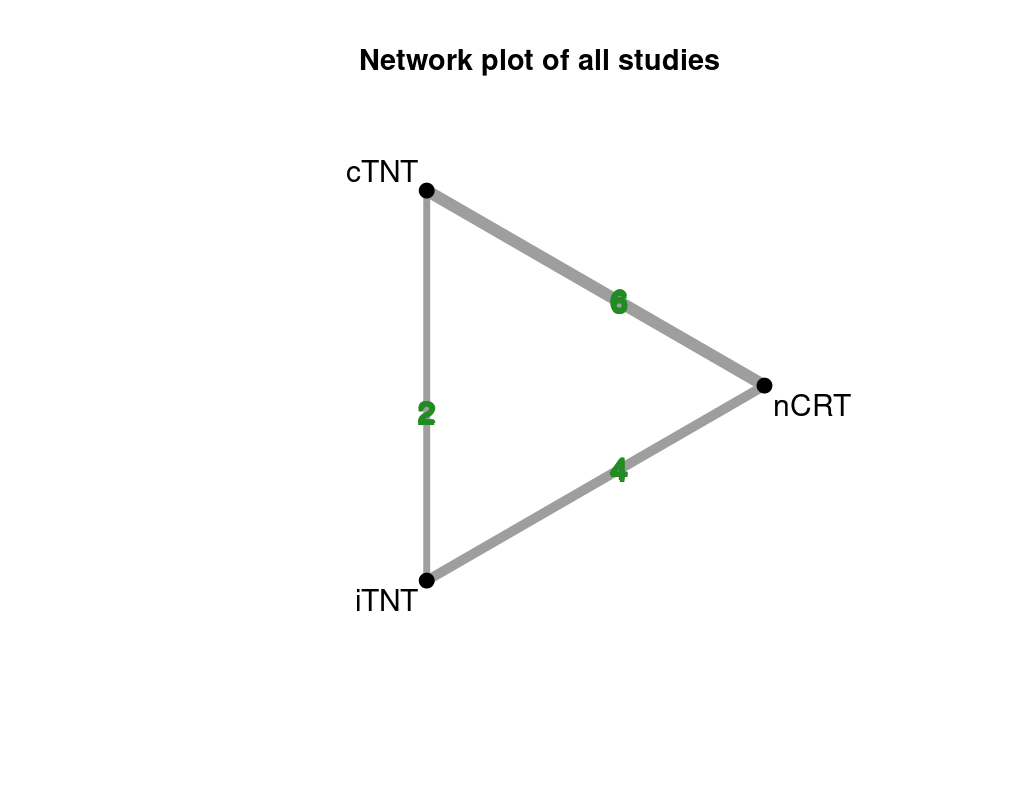 | 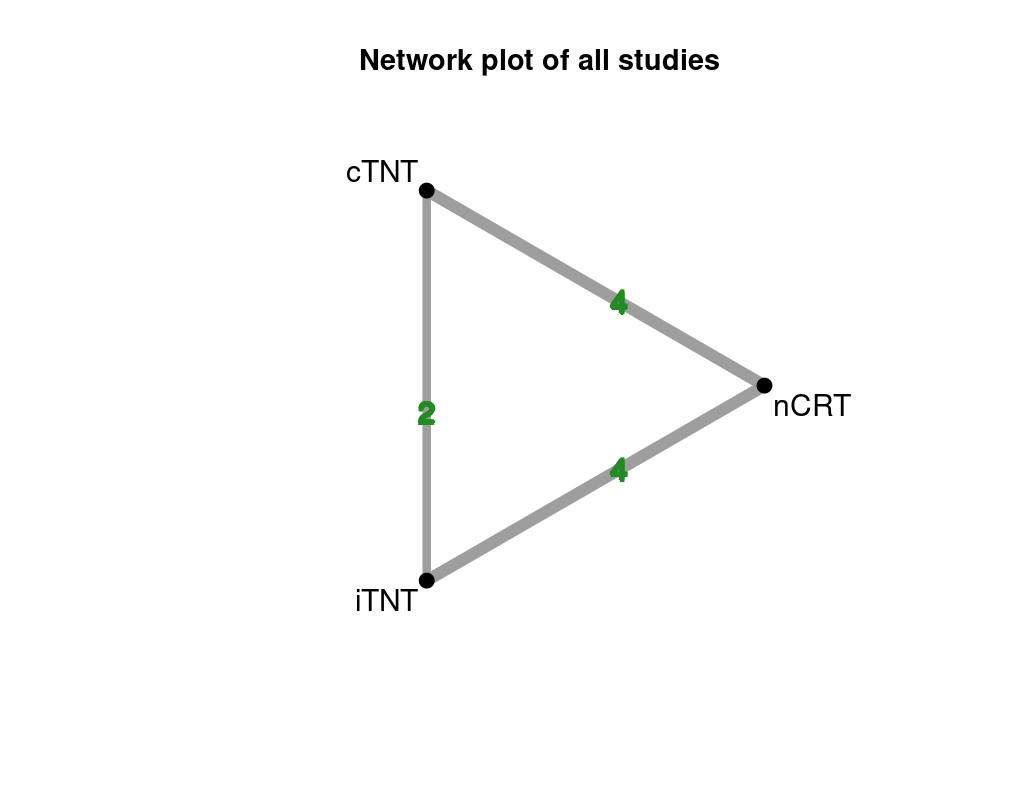 |
| Thrombocytopenia | Anaemia |
| 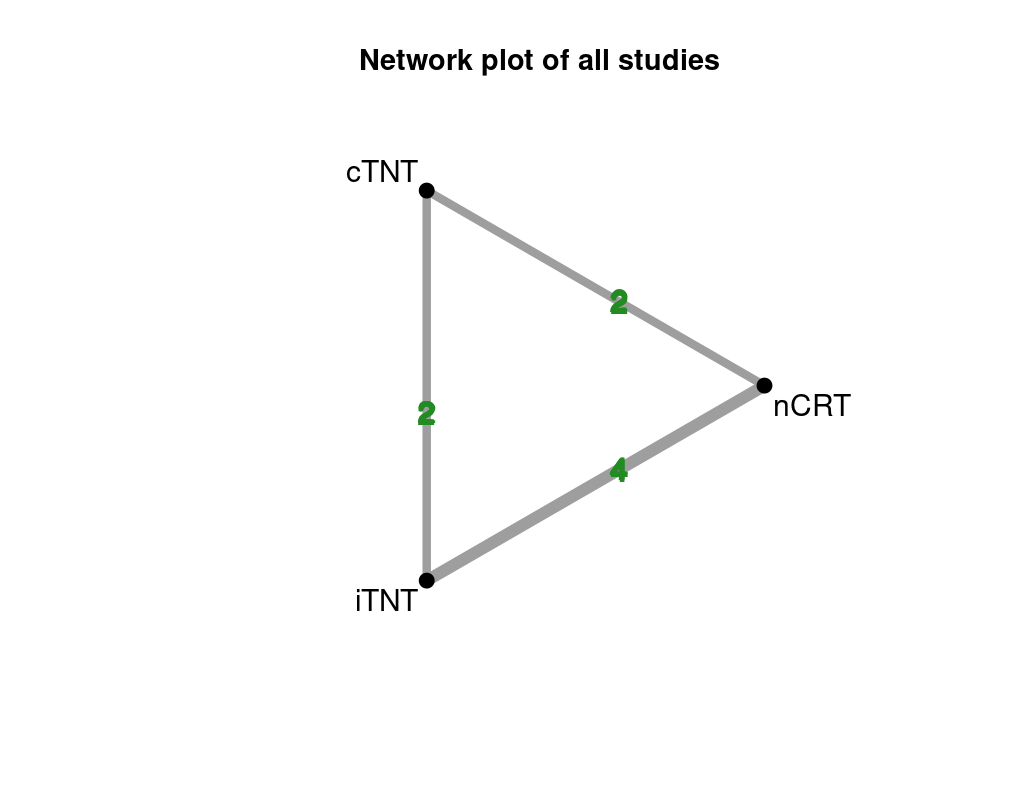 |  |
| Anaphylaxis |  |
| **Compliance outcomes** | |
| 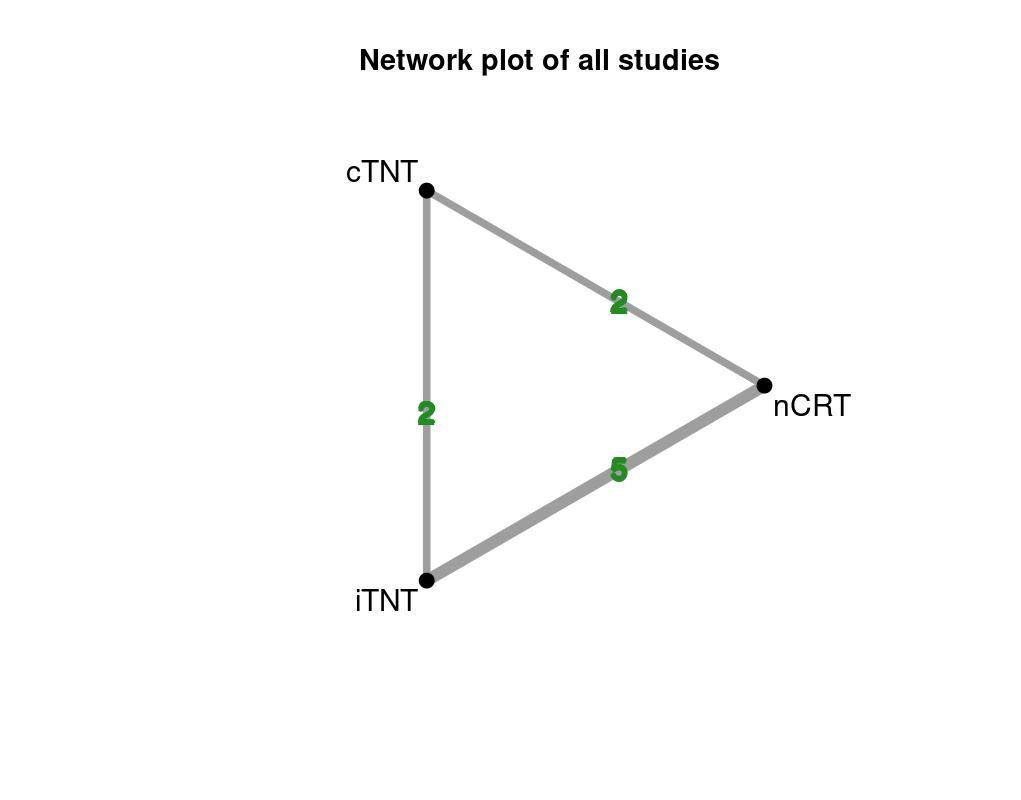 | 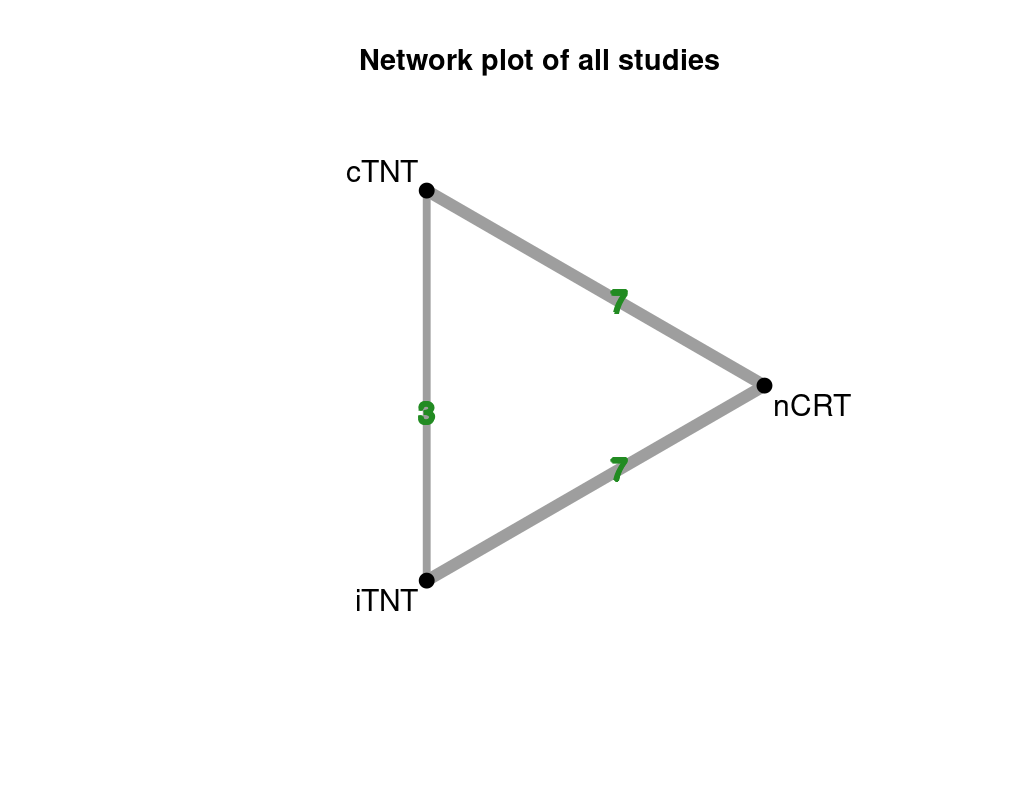 |
| Compliance with radiotherapy (≥4500Gy) | Compliance with chemotherapy (overall) >90% |
| 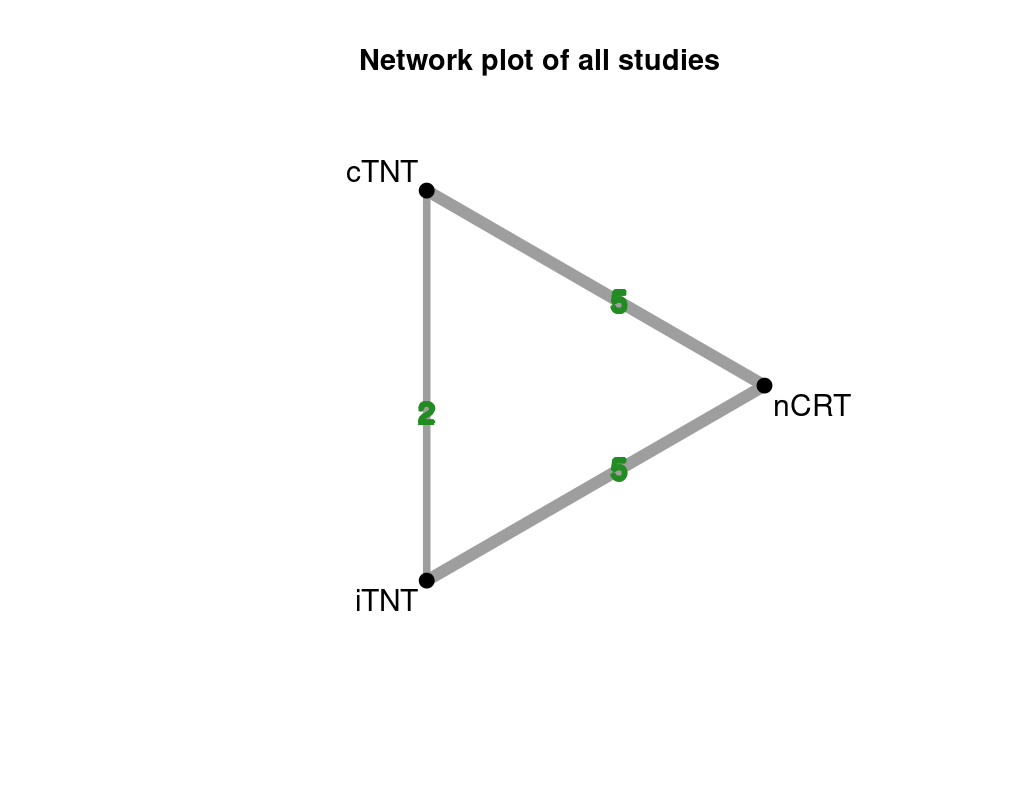 | 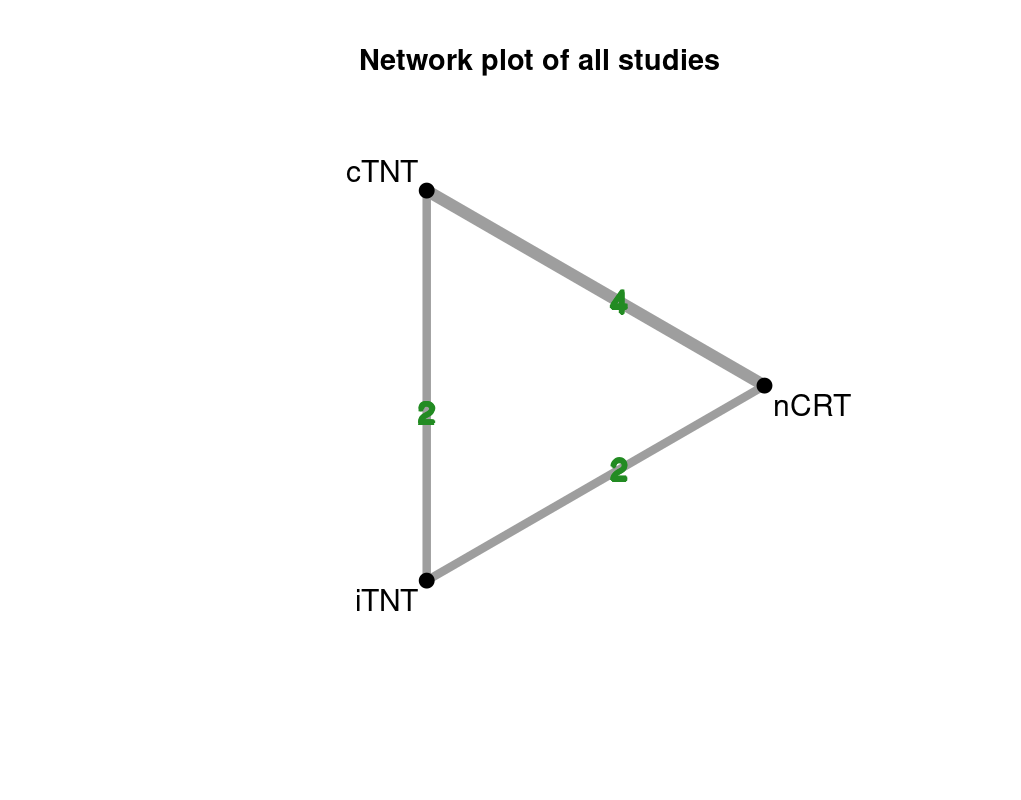 |
| Compliance with FOLFOX chemotherapy (>90%) | Compliance with CAPOX chemotherapy (>90%) |
| **Postoperative outcomes** | |
| 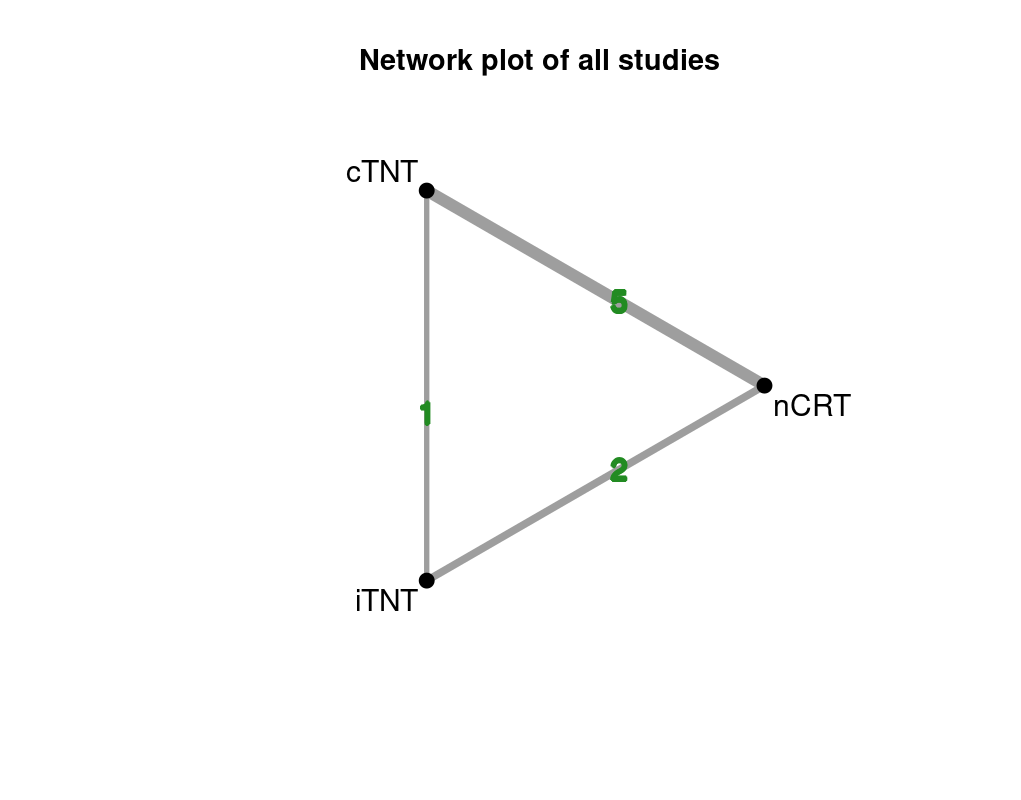 | 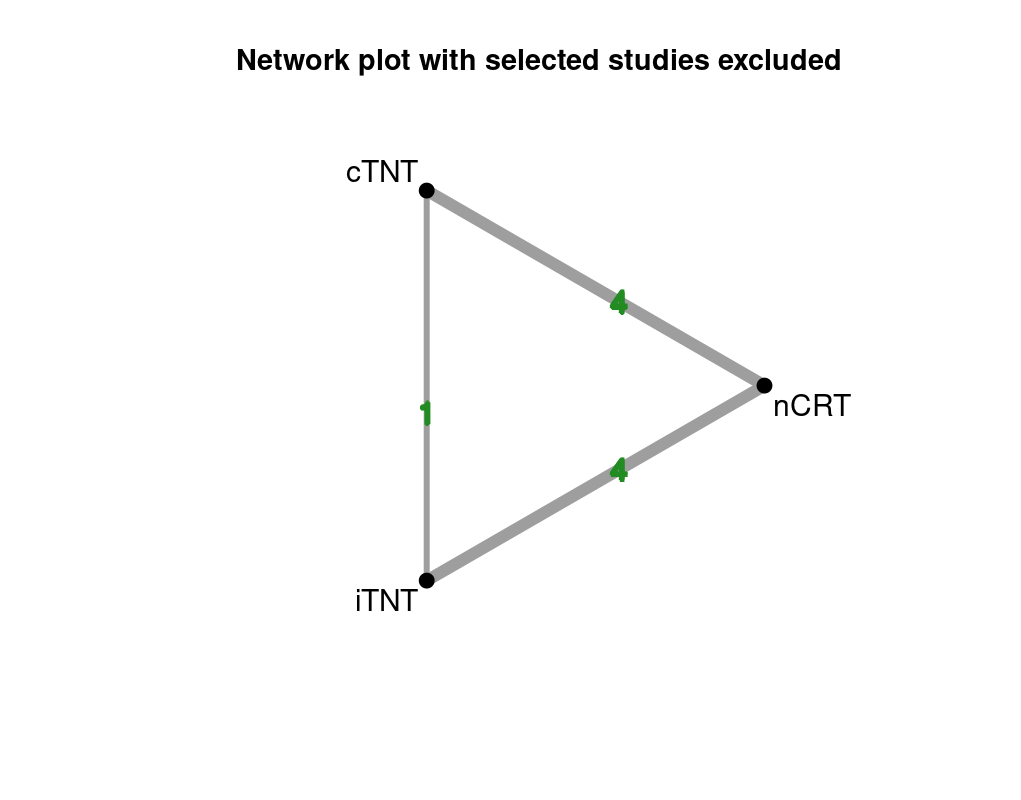 |
| Anastomotic leak | High stoma output/diarrhoea |
| 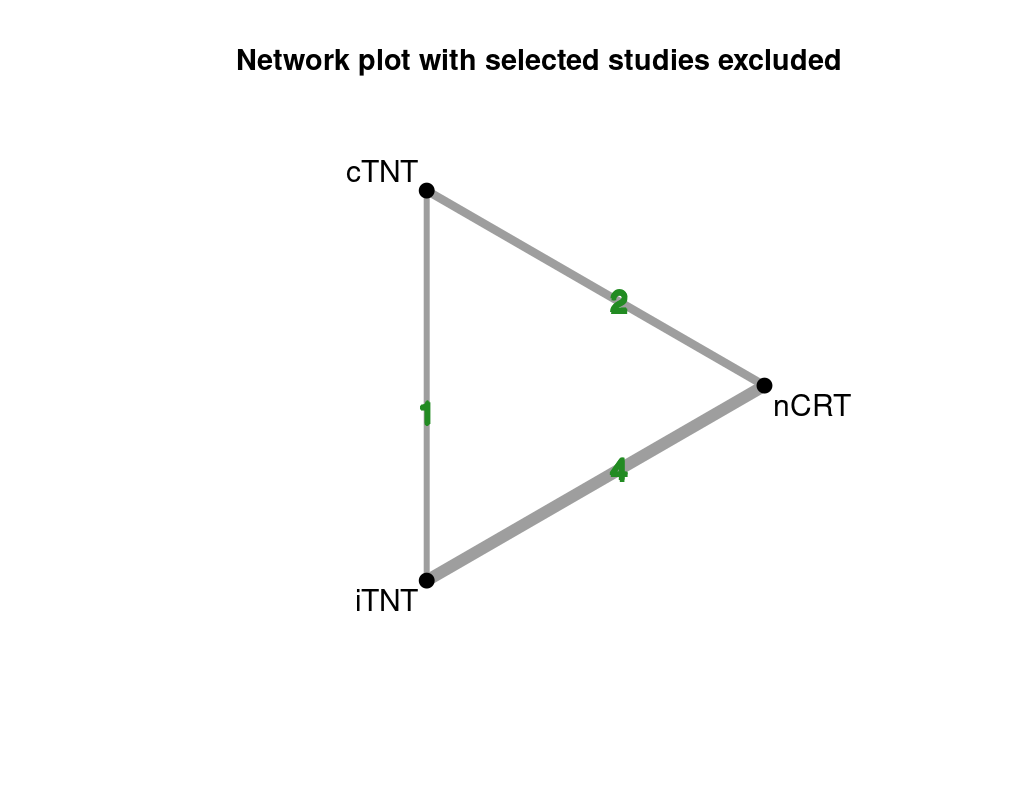 | 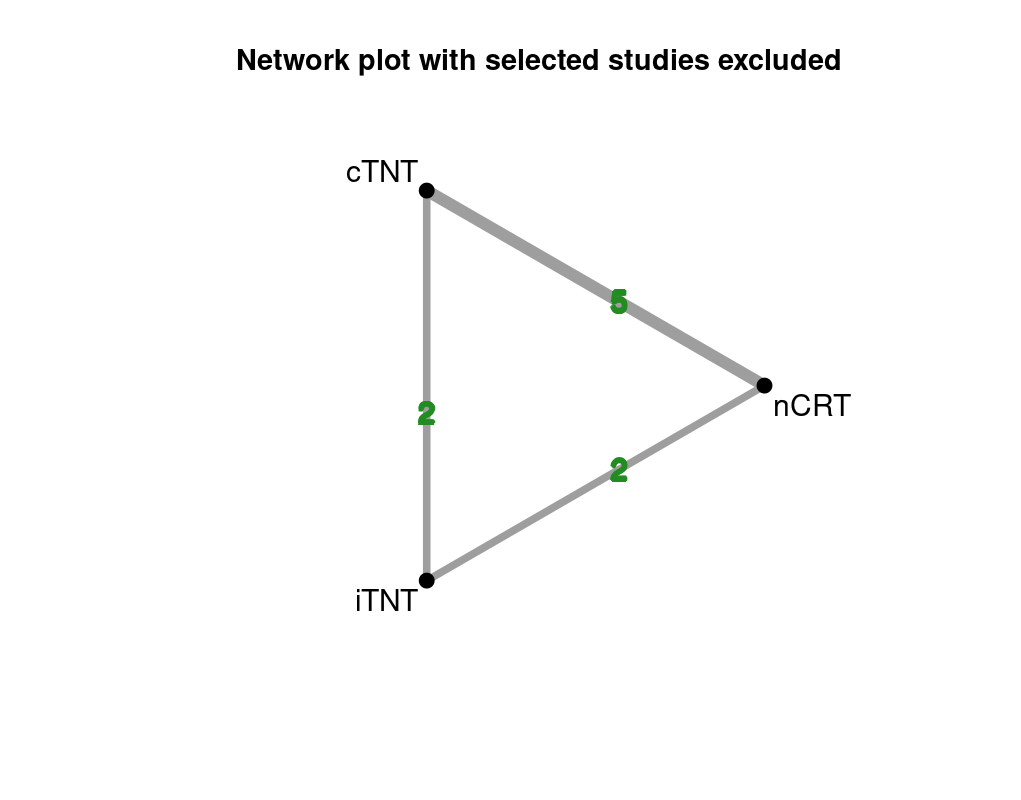 |
| Postoperative bowel obstruction | Organ/space surgical site infection |
| 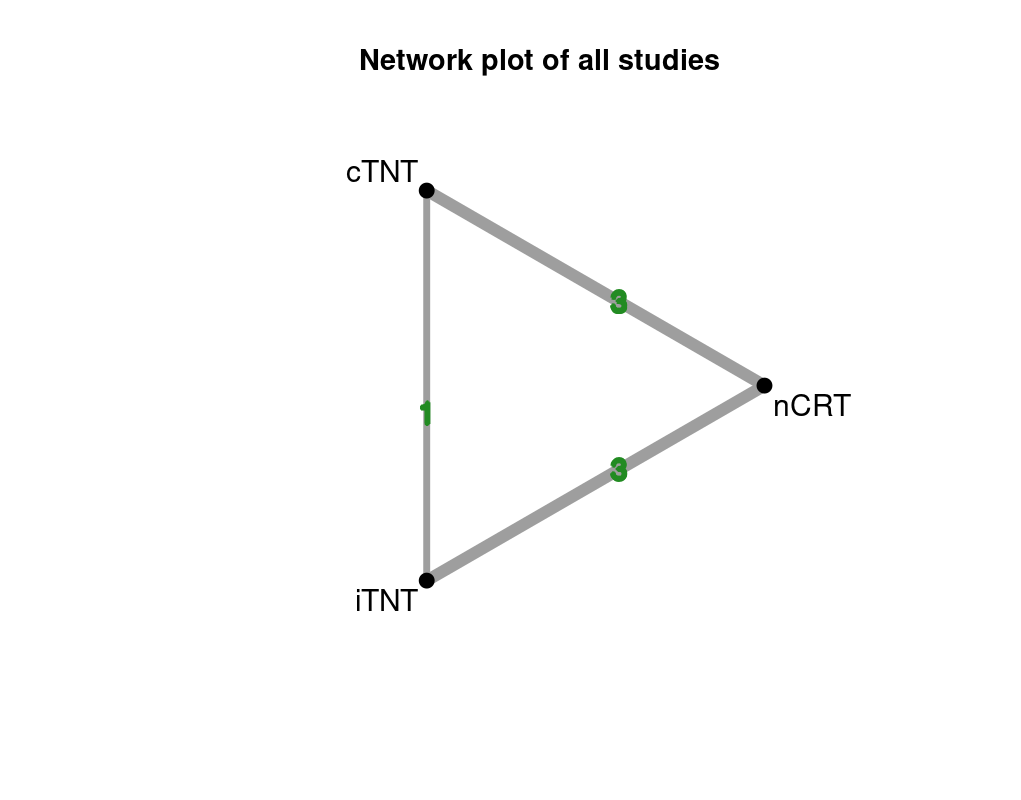 | 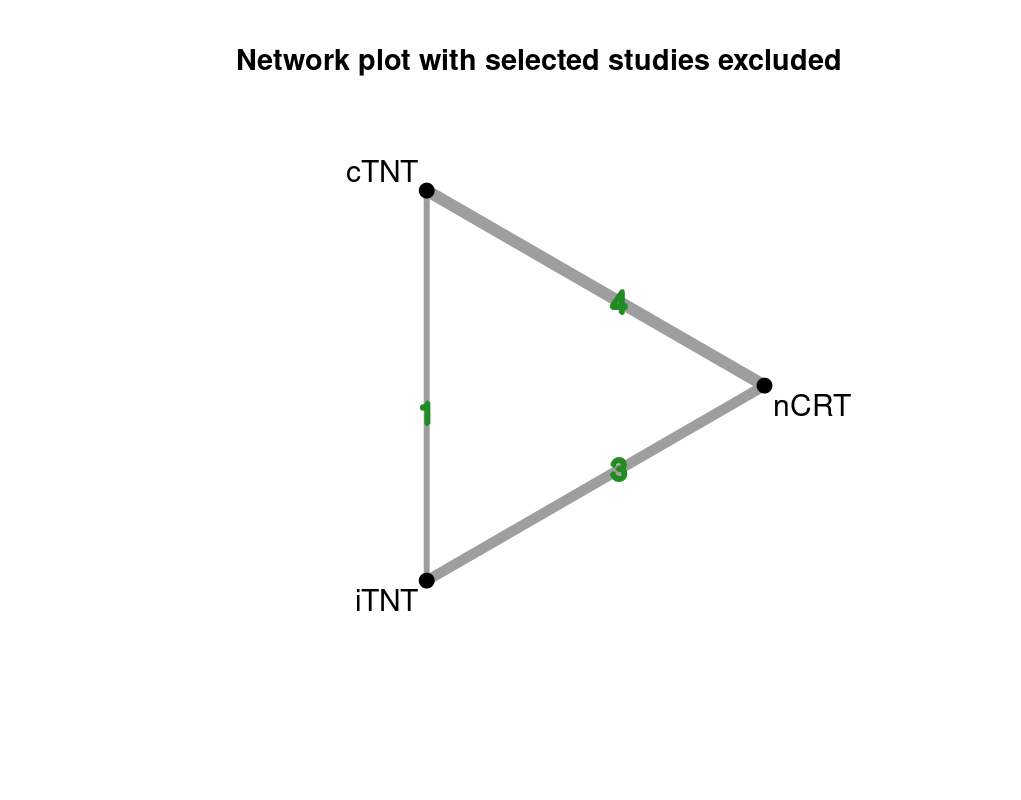 |
| Superficial incisional surgical site infection | Urinary tract infection |
| 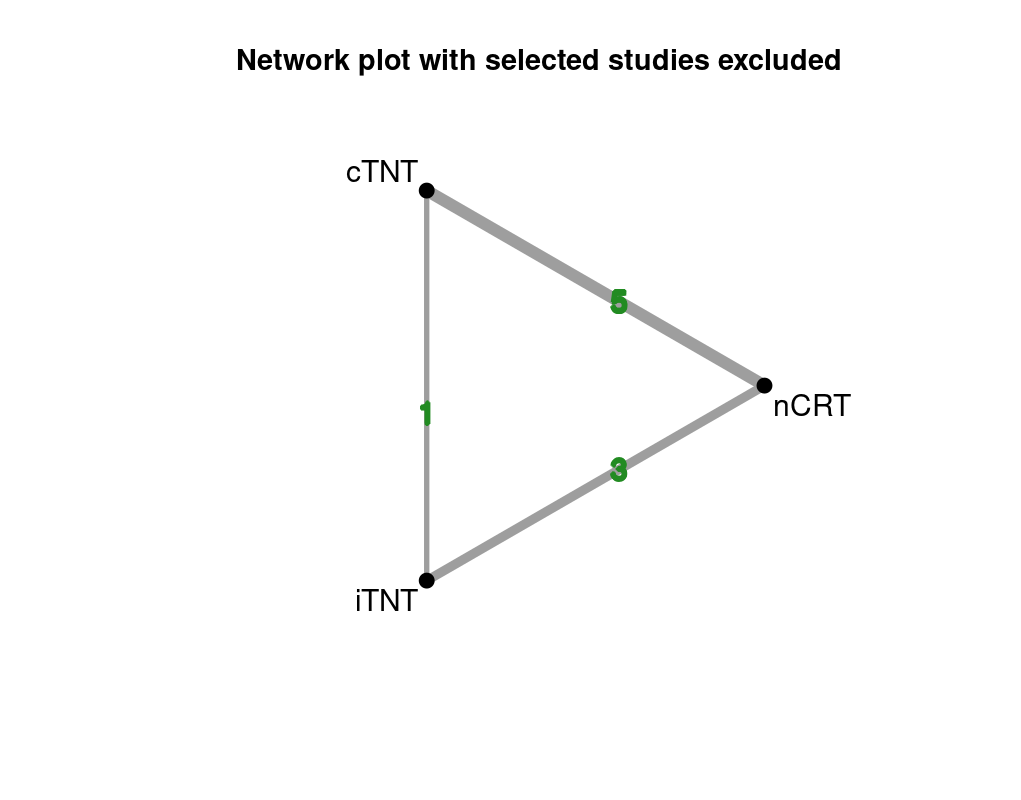 |  |
| Overall Clavien-Dindo Grade 3 and above postoperative complications |  |

## Appendix 8. League table of network meta-analysis relative estimates for all treatment comparison

**Toxicity outcomes**

| **Overall Grade III and above treatment-related adverse events** | | | |
| --- | --- | --- | --- |
|  | **cTNT** | **iTNT** | **nCRT** |
| **cTNT** | cTNT | 1.31 (0.62, 2.75) | 0.16 (0.61, 2.25) |
| **iTNT** | 0.76 (0.36, 1.61) | iTNT | 0.89 (0.51, 1.57) |
| **nCRT** | 0.86 (0.45, 1.63) | 1.13 (0.64, 1.97) | nCRT |
| **Individual Grade III and above treatment-related adverse events** | | | |
| Diarrhoea | | | |
|  | **cTNT** | **iTNT** | **nCRT** |
| **cTNT** | cTNT | 1.37 (0.26, 7.17) | 1.51 (0.38, 6.83) |
| **iTNT** | 0.73 (0.14, 3.82) | iTNT | 1.1 (0.3, 4.71) |
| **nCRT** | 0.66 (0.15, 2.61) | 0.91 (0.21, 3.37) | nCRT |
| Mucositis | | | |
|  | **cTNT** | **iTNT** | **nCRT** |
| **cTNT** | cTNT | 0.5 (0.13, 1.65) | 0.2 (0.04, 0.84) |
| **iTNT** | 2 (0.61, 7.58) | iTNT | 0.4 (0.11, 1.26) |
| **nCRT** | 5.04 (1.2, 28.18) | 2.49 (0.79, 9.46) | nCRT |
| Oesophagitis | | | |
|  | **cTNT** | **iTNT** | **nCRT** |
| **cTNT** | cTNT | 0.36 (0.08, 1.51) | 0.14 (0.01, 0.97) |
| **iTNT** | 2.76 (0.66, 13.06) | iTNT | 0.39 (0.03, 2.99) |
| **nCRT** | 7.07 (1.03, 81.25) | 2.58 (0.33, 29.33) | nCRT |
| Enterocolitis | | | |
|  | **cTNT** | **iTNT** | **nCRT** |
| **cTNT** | cTNT | 0.35 (0.08, 1.34) | 0.26 (0.06, 0.89) |
| **iTNT** | 2.82 (0.74, 13.33) | iTNT | 0.73 (0.17, 3.29) |
| **nCRT** | 3.87 (1.13, 15.86) | 1.37 (0.3, 5.94) | nCRT |
| Vomiting | | | |
|  | **cTNT** | **iTNT** | **nCRT** |
| **cTNT** | cTNT | 0.24 (0.05, 0.96) | 0.29 (0.08, 0.87) |
| **iTNT** | 4.16 (1.05, 20) | iTNT | 1.19 (0.31, 4.63) |
| **nCRT** | 3.51 (1.15, 12.71) | 0.84 (0.22, 3.25) | nCRT |
| Nausea without vomiting | | | |
|  | **cTNT** | **iTNT** | **nCRT** |
| **cTNT** | cTNT | 1.1 (0.28, 3.71) | 0.58 (0.2, 1.61) |
| **iTNT** | 0.91 (0.27, 3.61) | iTNT | 0.53 (0.19, 1.64) |
| **nCRT** | 1.74 (0.62, 5) | 1.9 (0.61, 5.31) | nCRT |
| Bowel obstruction | | | |
|  | **cTNT** | **iTNT** | **nCRT** |
| **cTNT** | cTNT | 1.46 (0.3, 7.79) | 0.37 (0.07, 1.78) |
| **iTNT** | 0.68 (0.13, 3.32) | iTNT | 0.25 (0.05, 1.06) |
| **nCRT** | 2.67 (0.56, 13.89) | 3.94 (0.94, 19.51) | nCRT |
| Pancreatitis | | | |
|  | **cTNT** | **iTNT** | **nCRT** |
| **cTNT** | cTNT | 0.41 (0.01, 5.87) | 0.36 (0.06, 1.85) |
| **iTNT** | 2.42 (0.17, 99.37) | iTNT | 0.88 (0.04, 47.16) |
| **nCRT** | 2.76 (0.54, 17.41) | 1.13 (0.02, 28.25) | nCRT |
| Proctitis | | | |
|  | **cTNT** | **iTNT** | **nCRT** |
| **cTNT** | cTNT | 5.12 (1.82, 17.6) | 1.03 (0.64, 1.75) |
| **iTNT** | 0.2 (0.06, 0.55) | iTNT | 0.2 (0.06, 0.51) |
| **nCRT** | 0.97 (0.57, 1.57) | 4.92 (1.96, 15.51) | nCRT |
| Rectal bleeding | | | |
|  | **cTNT** | **iTNT** | **nCRT** |
| **cTNT** | cTNT | 4.29 (1.07, 18.84) | 1.41 (0.49, 4.23) |
| **iTNT** | 0.23 (0.05, 0.93) | iTNT | 0.33 (0.09, 0.96) |
| **nCRT** | 0.71 (0.24, 2.03) | 3.05 (0.97, 11.36) | nCRT |
| Dizziness | | | |
|  | **cTNT** | **iTNT** | **nCRT** |
| **cTNT** | cTNT | 0.79 (0.21, 2.95) | 0.62 (0.22, 1.68) |
| **iTNT** | 1.26 (0.34, 4.8) | iTNT | 0.79 (0.18, 3.18) |
| **nCRT** | 1.62 (0.6, 4.51) | 1.26 (0.31, 5.65) | nCRT |
| Dysarthria | | | |
|  | **cTNT** | **iTNT** | **nCRT** |
| cTNT | cTNT | 1.6 (0.5, 5.29) | 1.14 (0.23, 5.43) |
| iTNT | 0.62 (0.19, 2.02) | iTNT | 0.7 (0.14, 3.56) |
| nCRT | 0.88 (0.18, 4.34) | 1.42 (0.28, 7.15) | nCRT |
| Hand-foot syndrome | | | |
|  | **cTNT** | **iTNT** | **nCRT** |
| **cTNT** | cTNT | 1.17 (0.41, 4.45) | 0.78 (0.34, 2.02) |
| **iTNT** | 0.85 (0.22, 2.42) | iTNT | 0.67 (0.19, 1.9) |
| **nCRT** | 1.28 (0.5, 2.92) | 1.5 (0.53, 5.22) | nCRT |
| Neuropathy | | | |
|  | **cTNT** | **iTNT** | **nCRT** |
| **cTNT** | cTNT | 0.49 (0.07, 2.57) | 0.1 (0.01, 0.42) |
| **iTNT** | 2.02 (0.39, 13.4) | iTNT | 0.2 (0.03, 0.77) |
| **nCRT** | 10.23 (2.4, 83.51) | 5.07 (1.3, 29.32) | nCRT |
| Syncope | | | |
|  | **cTNT** | **iTNT** | **nCRT** |
| **cTNT** | cTNT | 1.41 (0.35, 5.97) | 0.54 (0.2, 1.54) |
| **iTNT** | 0.71 (0.17, 2.84) | iTNT | 0.39 (0.08, 1.8) |
| **nCRT** | 1.84 (0.65, 4.99) | 2.58 (0.56, 12.48) | nCRT |
| Musculoskeletal weakness | | | |
|  | **cTNT** | **iTNT** | **nCRT** |
| **cTNT** | cTNT | 0.65 (0.29, 1.42) | 0.55 (0.27, 1.19) |
| **iTNT** | 1.55 (0.7, 3.43) | iTNT | 0.86 (0.54, 1.52) |
| **nCRT** | 1.8 (0.84, 3.65) | 1.17 (0.66, 1.84) | nCRT |
| Arrythmia | | | |
|  | **cTNT** | **iTNT** | **nCRT** |
| **cTNT** | cTNT | 0.93 (0.36, 2.83) | 1.05 (0.4, 2.77) |
| **iTNT** | 1.07 (0.35, 2.77) | iTNT | 1.12 (0.38, 2.8) |
| **nCRT** | 0.95 (0.36, 2.49) | 0.89 (0.36, 2.63) | nCRT |
| Venous thromboembolism | | | |
|  | **cTNT** | **iTNT** | **nCRT** |
| **cTNT** | cTNT | 1.66 (0.64, 4.56) | 0.36 (0.17, 0.69) |
| **iTNT** | 0.6 (0.22, 1.56) | iTNT | 0.21 (0.07, 0.59) |
| **nCRT** | 2.81 (1.46, 5.94) | 4.66 (1.7, 14.6) | nCRT |
| Pneumonia | | | |
|  | **cTNT** | **iTNT** | **nCRT** |
| **cTNT** | cTNT | 2.32 (0.59, 11.48) | 0.6 (0.16, 2.89) |
| **iTNT** | 0.43 (0.09, 1.71) | iTNT | 0.26 (0.04, 1.53) |
| **nCRT** | 1.66 (0.35, 6.41) | 3.8 (0.66, 23.59) | nCRT |
| Urinary tract infection | | | |
|  | **cTNT** | **iTNT** | **nCRT** |
| **cTNT** | cTNT | 0.73 (0.28, 1.88) | 0.38 (0.13, 1.09) |
| **iTNT** | 1.36 (0.53, 3.6) | iTNT | 0.52 (0.21, 1.27) |
| **nCRT** | 2.6 (0.92, 7.98) | 1.92 (0.79, 4.85) | nCRT |
| Sepsis | | | |
|  | **cTNT** | **iTNT** | **nCRT** |
| **cTNT** | cTNT | 0.37 (0.05, 1.99) | 0.67 (0.13, 3.02) |
| **iTNT** | 2.73 (0.5, 18.88) | iTNT | 1.86 (0.41, 8.64) |
| **nCRT** | 1.5 (0.33, 7.58) | 0.54 (0.12, 2.44) | nCRT |
| Radiation dermatitis | | | |
|  | **cTNT** | **iTNT** | **nCRT** |
| **cTNT** | cTNT | 0.84 (0.13, 4.82) | 0.84 (0.18, 3.23) |
| **iTNT** | 1.19 (0.21, 7.94) | iTNT | 1 (0.14, 6.89) |
| **nCRT** | 1.19 (0.31, 5.46) | 1 (0.15, 7.11) | nCRT |
| Renal and electrolyte imbalances | | | |
|  | **cTNT** | **iTNT** | **nCRT** |
| **cTNT** | cTNT | 0.7 (0.25, 1.46) | 1.37 (0.59, 3.08) |
| **iTNT** | 1.43 (0.69, 4.01) | iTNT | 1.98 (0.8, 5.83) |
| **nCRT** | 0.73 (0.32, 1.71) | 0.51 (0.17, 1.26) | nCRT |
| Febrile neutropaenia | | | |
|  | **cTNT** | **iTNT** | **nCRT** |
| **cTNT** | cTNT | 0.52 (0.11, 2.06) | 0.22 (0.05, 0.79) |
| **iTNT** | 1.94 (0.49, 9.1) | iTNT | 0.43 (0.13, 1.24) |
| **nCRT** | 4.52 (1.26, 19.59) | 2.32 (0.81, 7.59) | nCRT |
| Neutropaenia | | | |
|  | **cTNT** | **iTNT** | **nCRT** |
| **cTNT** | cTNT | 0.52 (0.09, 2.35) | 0.11 (0.02, 0.38) |
| **iTNT** | 1.94 (0.43, 11.4) | iTNT | 0.22 (0.04, 0.63) |
| **nCRT** | 8.89 (2.65, 59.87) | 4.48 (1.58, 22.36) | nCRT |
| Lymphopenia | | | |
|  | **cTNT** | **iTNT** | **nCRT** |
| **cTNT** | cTNT | 0.56 (0.34, 0.99) | 0.62 (0.37, 1.14) |
| **iTNT** | 1.79 (1, 2.94) | iTNT | 1.11 (0.74, 1.75) |
| **nCRT** | 1.62 (0.87, 2.69) | 0.9 (0.57, 1.36) | nCRT |
| Thrombocytopenia | | | |
|  | **cTNT** | **iTNT** | **nCRT** |
| **cTNT** | cTNT | 0.84 (0.14, 6.23) | 0.52 (0.14, 2.64) |
| **iTNT** | 1.19 (0.16, 7.26) | iTNT | 0.63 (0.12, 3.63) |
| **nCRT** | 1.92 (0.38, 6.97) | 1.59 (0.28, 8.65) | nCRT |
| Anaemia | | | |
|  | **cTNT** | **iTNT** | **nCRT** |
| **cTNT** | cTNT | 0.76 (0.17, 3.26) | 0.35 (0.11, 1.08) |
| **iTNT** | 1.31 (0.31, 5.77) | iTNT | 0.47 (0.13, 1.47) |
| **nCRT** | 2.82 (0.93, 9.48) | 2.14 (0.68, 7.95) | nCRT |
| Anaphylaxis | | | |
|  | **cTNT** | **iTNT** | **nCRT** |
| **cTNT** | cTNT | 1.21 (0.29, 5.07) | 0.55 (0.13, 2.08) |
| **iTNT** | 0.82 (0.2, 3.39) | iTNT | 0.46 (0.12, 1.51) |
| **nCRT** | 1.8 (0.48, 7.44) | 2.17 (0.66, 8.27) | nCRT |
| Treatment-related mortality | | | |
|  | **cTNT** | **iTNT** | **nCRT** |
| **cTNT** | cTNT | 0.55 (0.11, 2.43) | 0.81 (0.19, 3.15) |
| **iTNT** | 1.82 (0.41, 9.42) | iTNT | 1.48 (0.4, 5.24) |
| **nCRT** | 1.24 (0.32, 5.39) | 0.67 (0.19, 2.5) | nCRT |

**Compliance outcomes**

| Compliance with radiotherapy (≥4500Gy) | | | |
| --- | --- | --- | --- |
|  | **cTNT** | **iTNT** | **nCRT** |
| **cTNT** | cTNT | 0.23 (0.05, 0.72) | 0.18 (0.04, 0.58) |
| **iTNT** | 4.4 (1.39, 18.26) | iTNT | 0.77 (0.31, 1.84) |
| **nCRT** | 5.71 (1.72, 25.55) | 1.3 (0.54, 3.26) | nCRT |
| Compliance with chemotherapy (>90%) | | | |
|  | **cTNT** | **iTNT** | **nCRT** |
| **cTNT** | cTNT | 0.92 (0.28, 2.93) | 1.09 (0.41, 3.11) |
| **iTNT** | 1.09 (0.34, 3.59) | iTNT | 1.19 (0.46, 3.41) |
| **nCRT** | 0.92 (0.32, 2.45) | 0.84 (0.29, 2.17) | nCRT |
| Compliance with FOLFOX chemotherapy (>90%) | | | |
|  | **cTNT** | **iTNT** | **nCRT** |
| **cTNT** | cTNT | 1.17 (0.68, 2.04) | - |
| **iTNT** | 0.85 (0.49, 1.47) | iTNT | - |
| Compliance with CAPOX chemotherapy (>90%) | | | |
|  | **cTNT** | **iTNT** | **nCRT** |
| **cTNT** | cTNT | 0.88 (0.53, 1.46) | - |
| **iTNT** | 1.13 (0.68, 1.89) | iTNT | - |

**Postoperative outcomes**

| Overall Clavien-Dindo Grade 3 and above postoperative complications | | | |
| --- | --- | --- | --- |
|  | **cTNT** | **iTNT** | **nCRT** |
| **cTNT** | cTNT | 0.99 (0.56, 1.67) | 0.44 (0.24, 0.69) |
| **iTNT** | 1.01 (0.6, 1.79) | iTNT | 0.44 (0.26, 0.68) |
| **nCRT** | 2.28 (1.46, 4.16) | 2.26 (1.47, 3.81) | nCRT |
| Anastomotic leak | | | |
|  | **cTNT** | **iTNT** | **nCRT** |
| **cTNT** | cTNT | 1.49 (0.55, 4.43) | 1.53 (0.65, 3.8) |
| **iTNT** | 0.67 (0.23, 1.82) | iTNT | 1.03 (0.39, 2.54) |
| **nCRT** | 0.65 (0.26, 1.53) | 0.97 (0.39, 2.53) | nCRT |
| High stoma output/diarrhoea | | | |
|  | **cTNT** | **iTNT** | **nCRT** |
| **cTNT** | cTNT | 2.71 (1.37, 5.67) | 0.91 (0.46, 1.81) |
| **iTNT** | 0.37 (0.18, 0.73) | iTNT | 0.33 (0.15, 0.71) |
| **nCRT** | 1.1 (0.55, 2.16) | 3 (1.41, 6.5) | nCRT |
| Postoperative bowel obstruction | | | |
|  | **cTNT** | **iTNT** | **nCRT** |
| **cTNT** | cTNT | 1.13 (0.63, 1.97) | 1.63 (0.86, 3.01) |
| **iTNT** | 0.88 (0.51, 1.59) | iTNT | 1.44 (0.9, 2.31) |
| **nCRT** | 0.61 (0.33, 1.16) | 0.69 (0.43, 1.11) | nCRT |
| Organ/space surgical site infection | | | |
|  | **cTNT** | **iTNT** | **nCRT** |
| **cTNT** | cTNT | 1.58 (0.75, 3.31) | 1.75 (1, 2.94) |
| **iTNT** | 0.63 (0.3, 1.33) | iTNT | 1.1 (0.51, 2.31) |
| **nCRT** | 0.57 (0.34, 1) | 0.91 (0.43, 1.98) | nCRT |
| Superficial incisional surgical site infection | | | |
|  | **cTNT** | **iTNT** | **nCRT** |
| **cTNT** | cTNT | 0.78 (0.26, 1.84) | 0.94 (0.37, 1.88) |
| **iTNT** | 1.28 (0.54, 3.89) | iTNT | 1.2 (0.6, 2.46) |
| **nCRT** | 1.06 (0.53, 2.69) | 0.83 (0.41, 1.66) | nCRT |
| Postoperative urinary tract infection | | | |
|  | **cTNT** | **iTNT** | **nCRT** |
| **cTNT** | cTNT | 1.66 (0.74, 4.06) | 1.07 (0.48, 2.56) |
| **iTNT** | 0.6 (0.25, 1.34) | iTNT | 0.64 (0.31, 1.3) |
| **nCRT** | 0.94 (0.39, 2.08) | 1.57 (0.77, 3.27) | nCRT |

cTNT: consolidation TNT; iTNT: induction TNT; nCRT: neoadjuvant chemoradiation; Gy: gray, international unit (SI) for radiation dose

## Appendix 9: Further subgroup analysis

Short-course radiotherapy vs. Long-course radiotherapy

| **Long-course radiotherapy*** | |
| --- | --- |
| Compliance to chemotherapy | 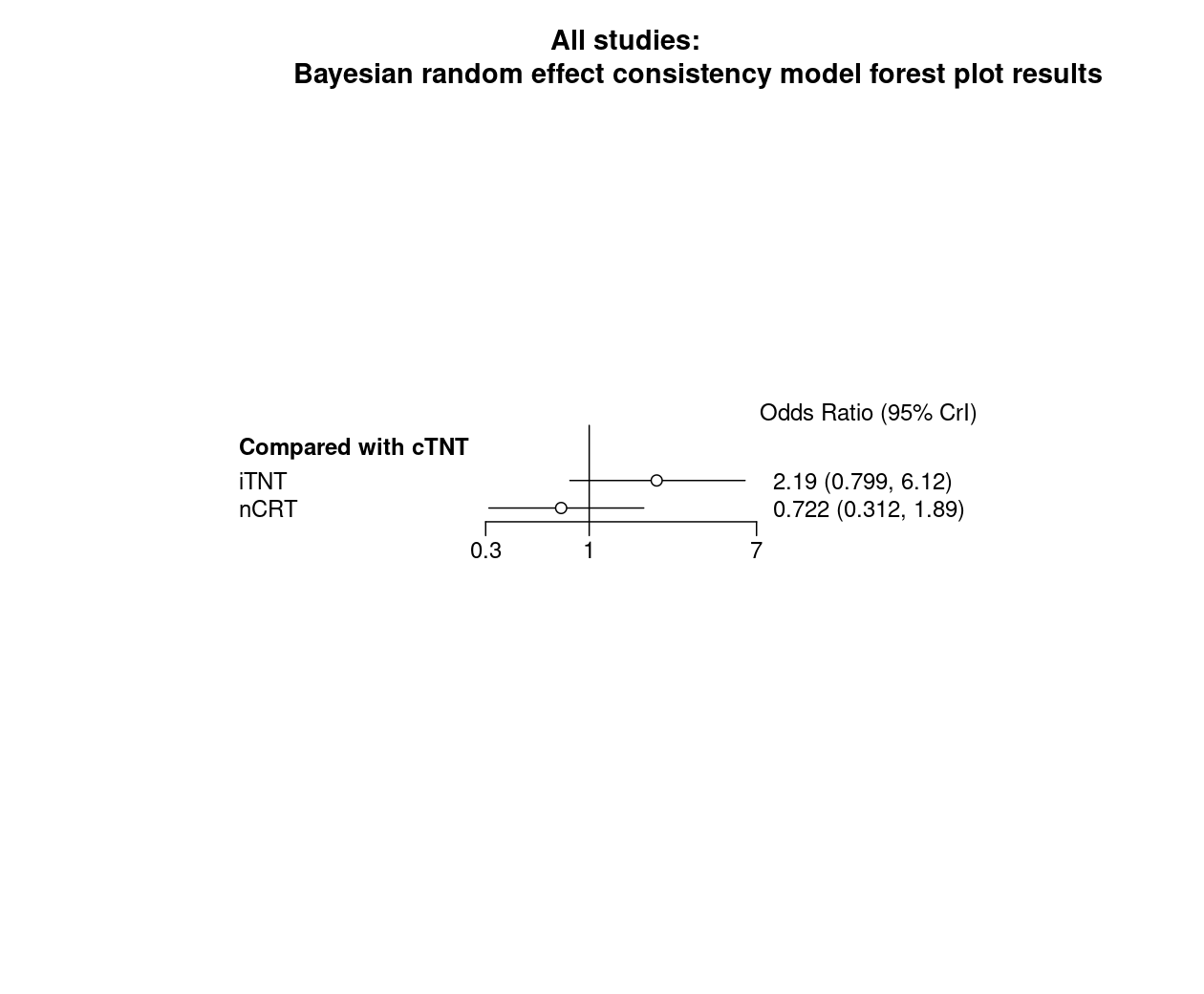 |
| Compliance to FOLFOX-specific chemotherapy | 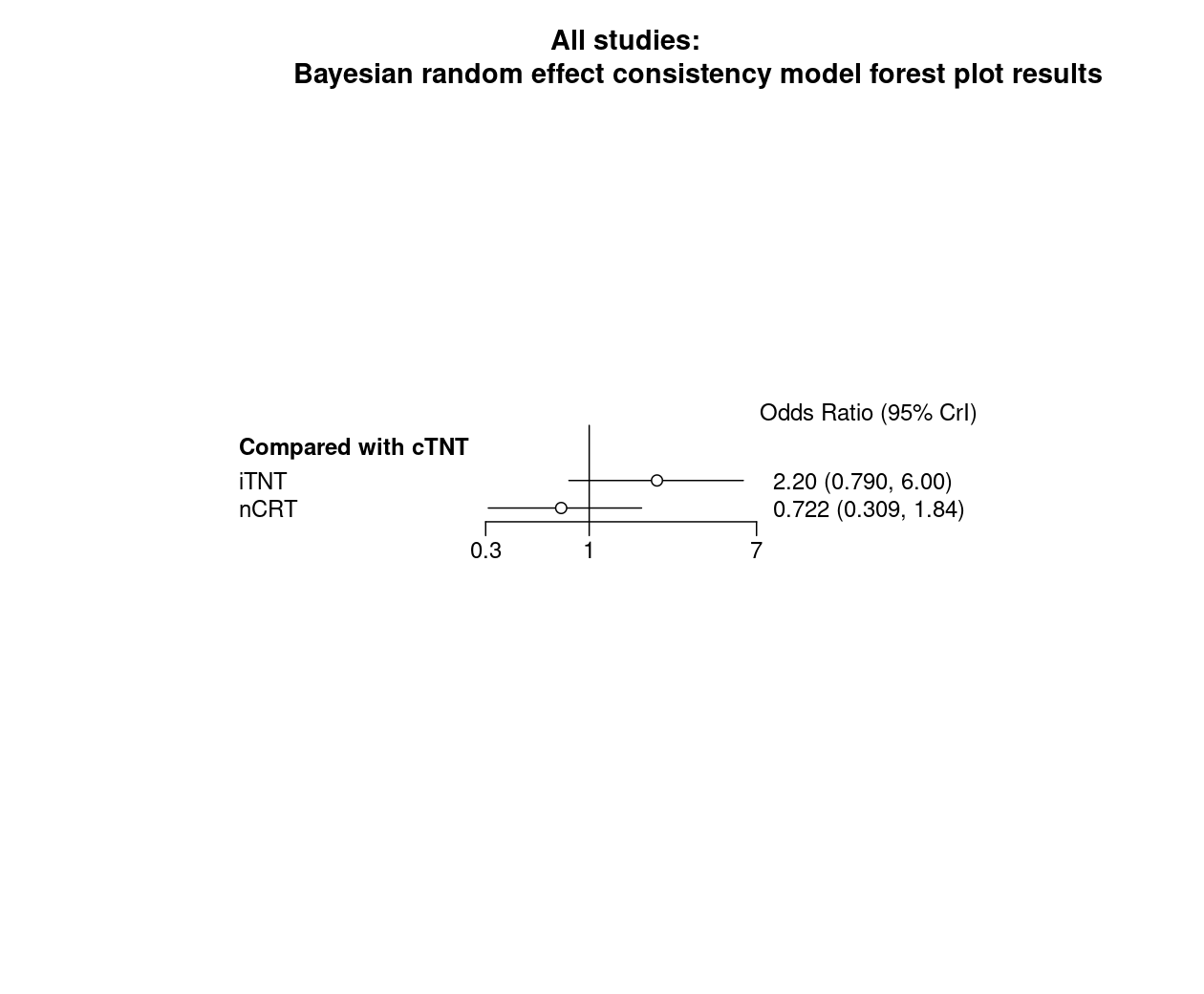 |
| Compliance to CAPOX-specific chemotherapy | 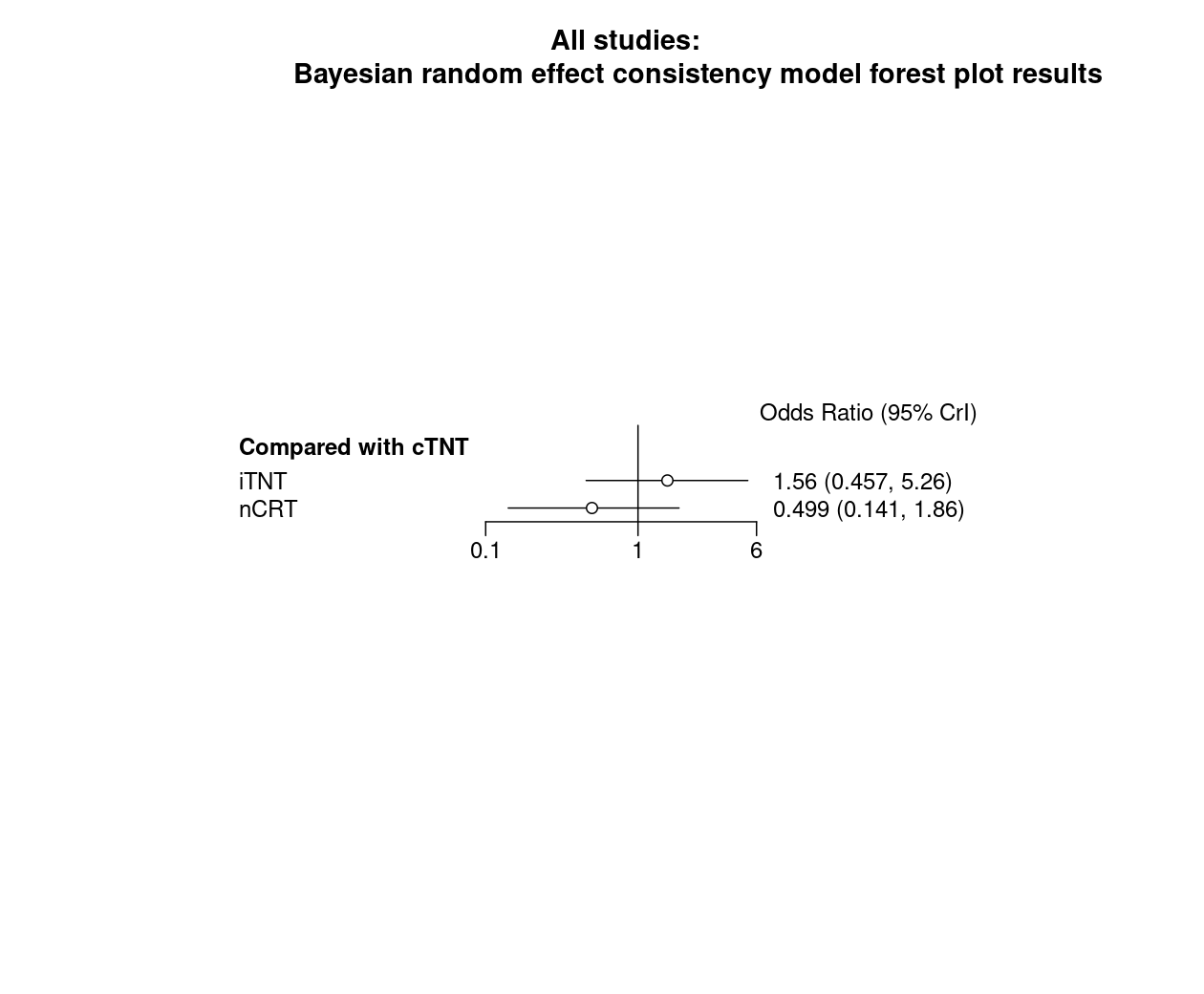 |
| Compliance to long-course radiotherapy | 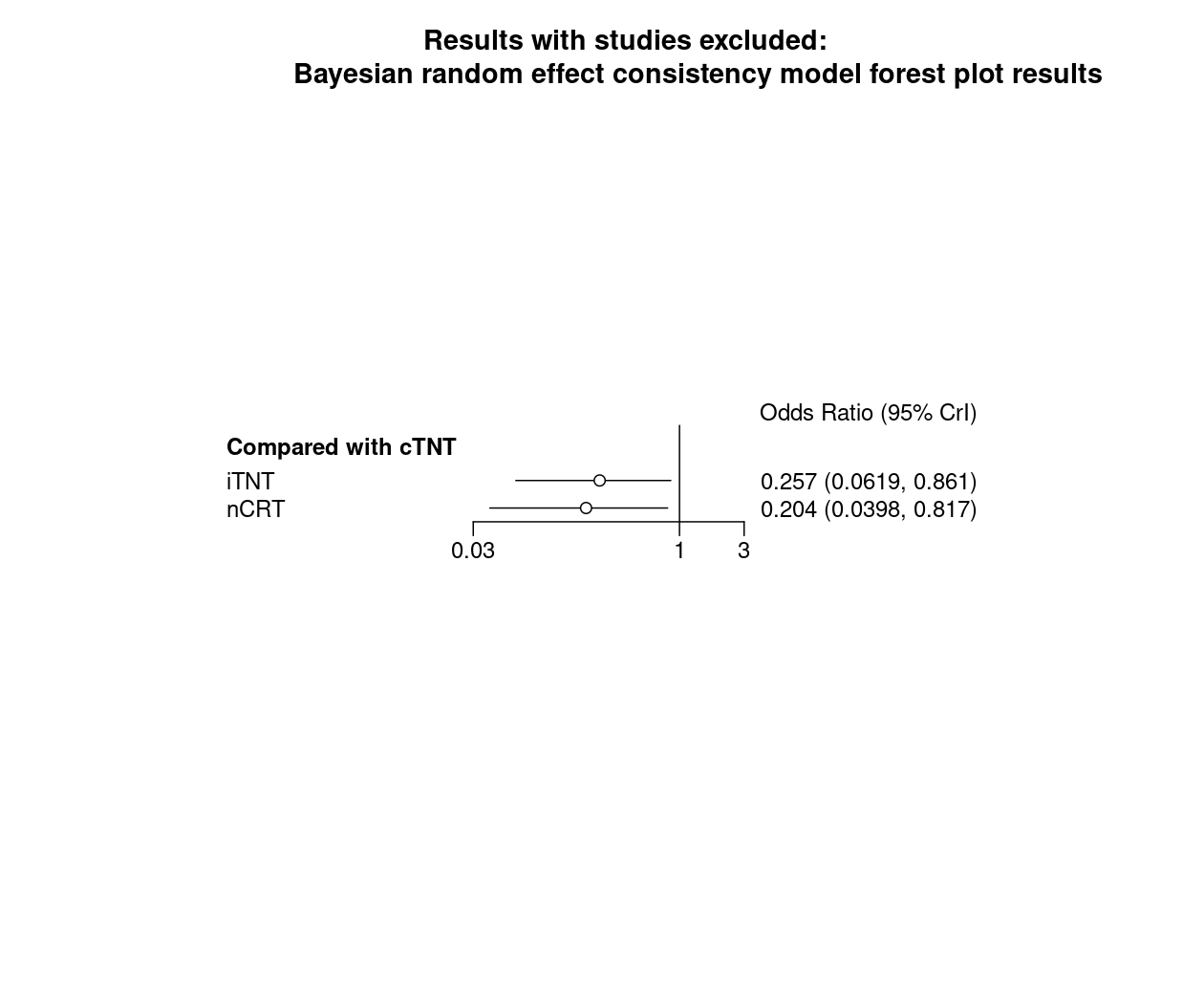 |
| Overall toxicity | 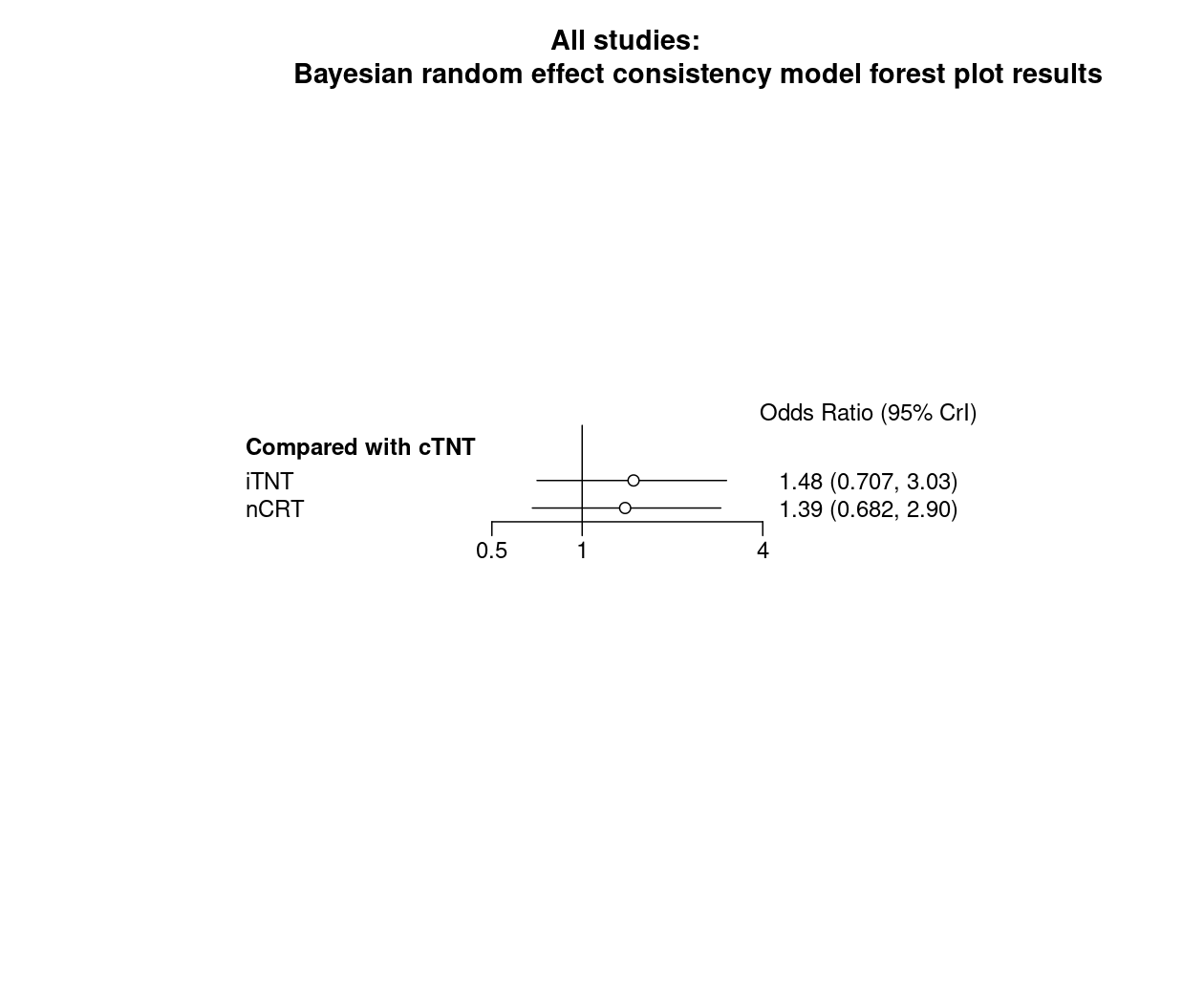 |
| Postoperative complications | 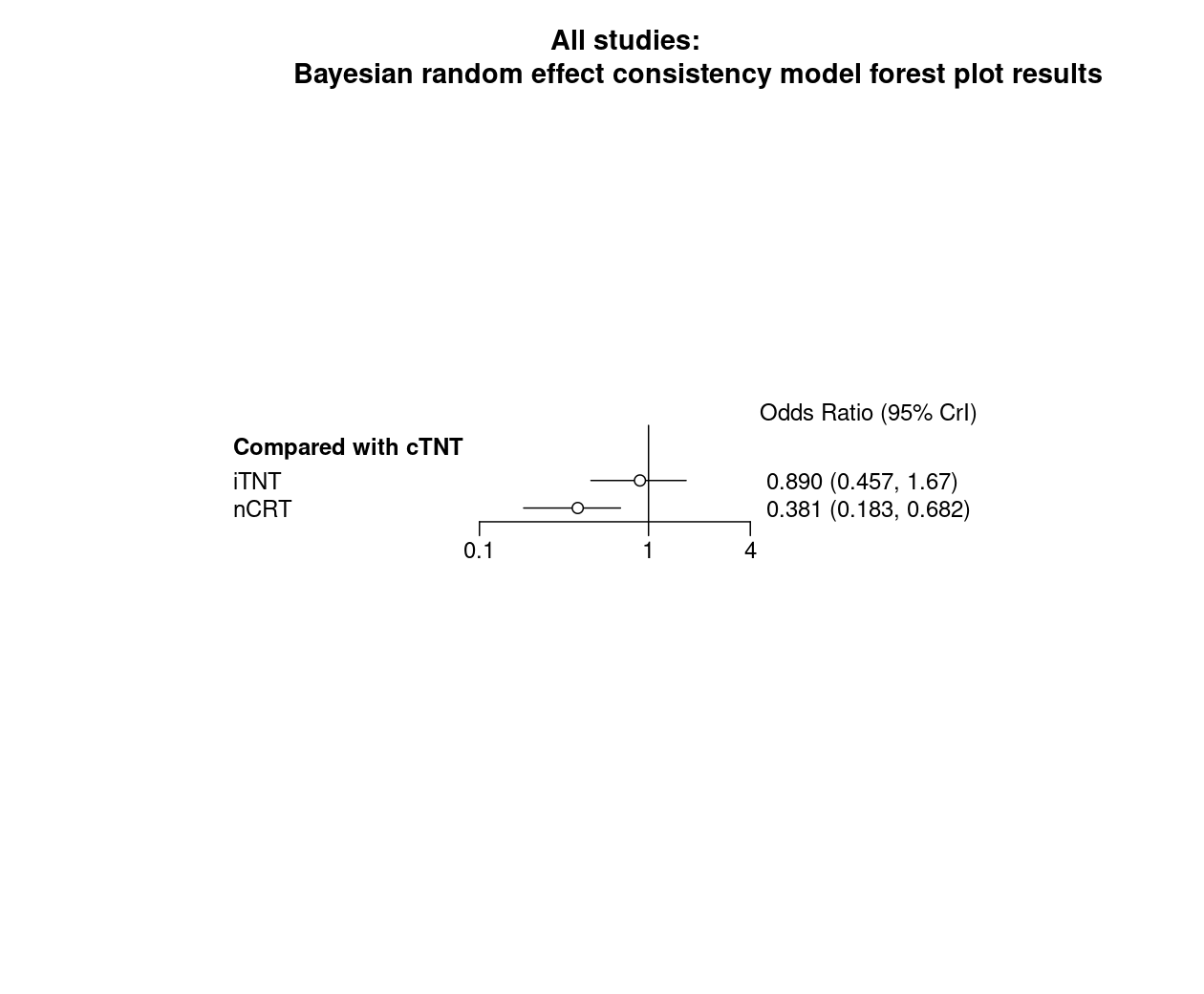 |
| **Short-course radiotherapy**** | |
| Compliance to chemotherapy | 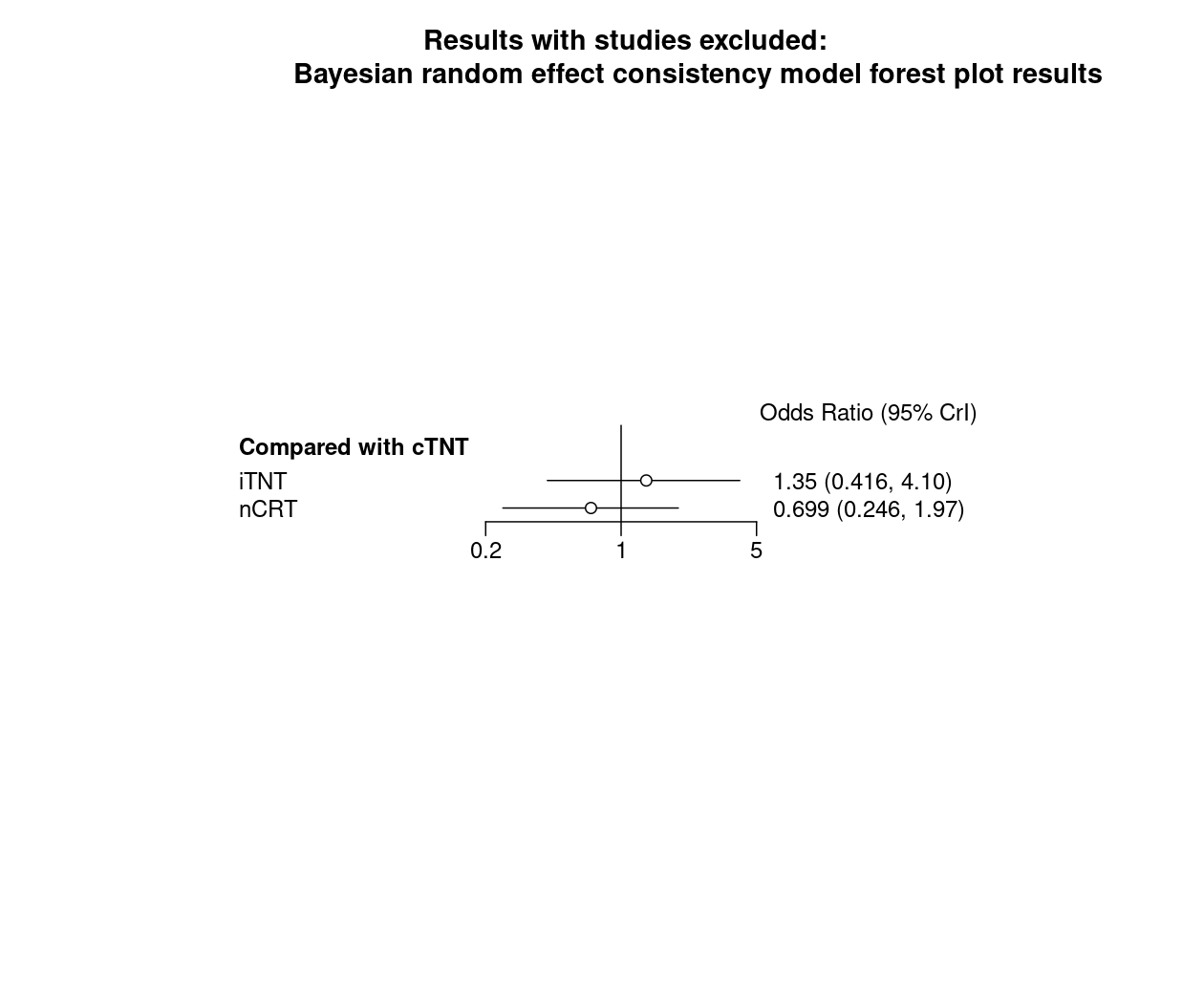 |
| Compliance to FOLFOX-specific chemotherapy | 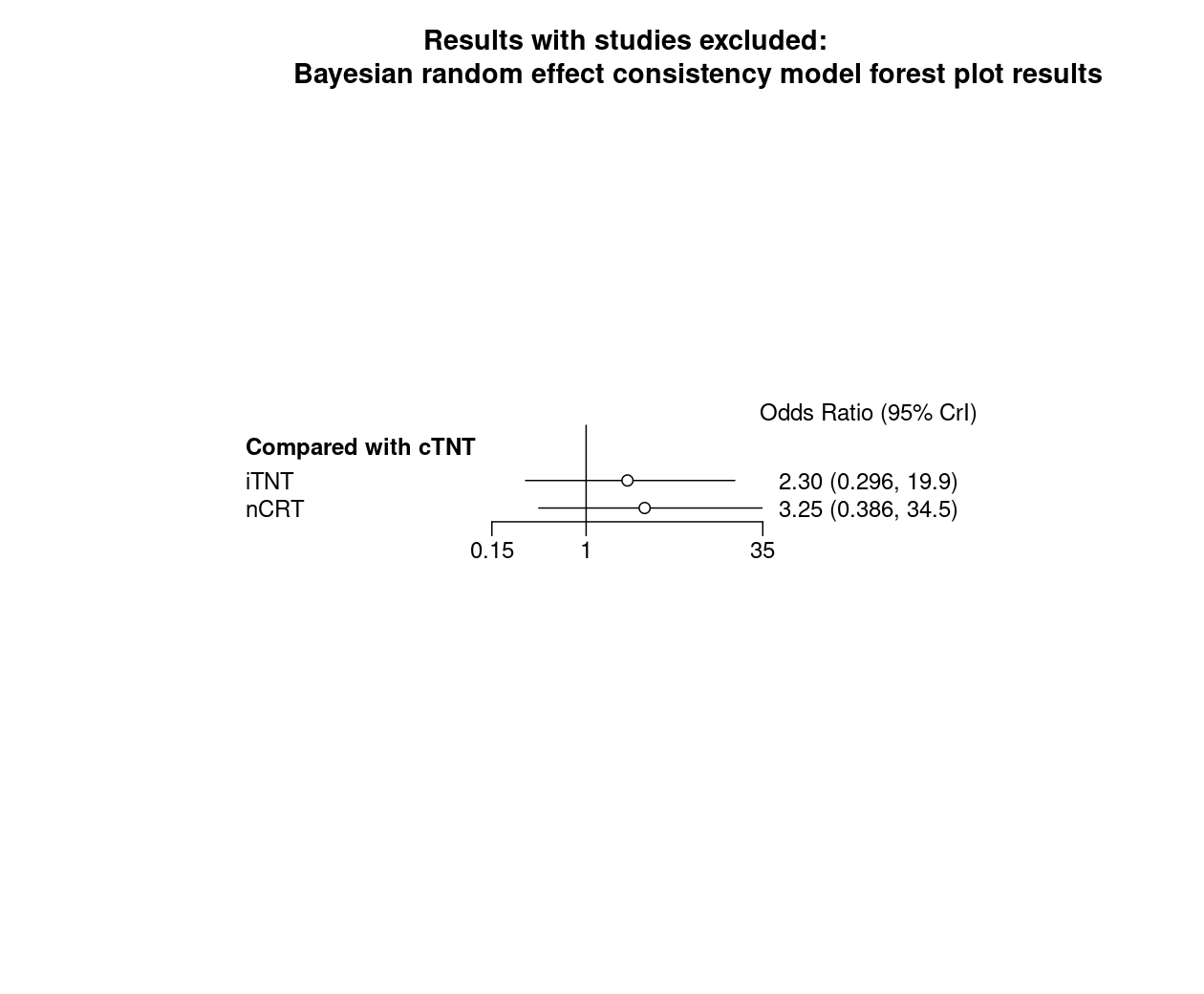 |
| Compliance to CAPOX-specific chemotherapy | 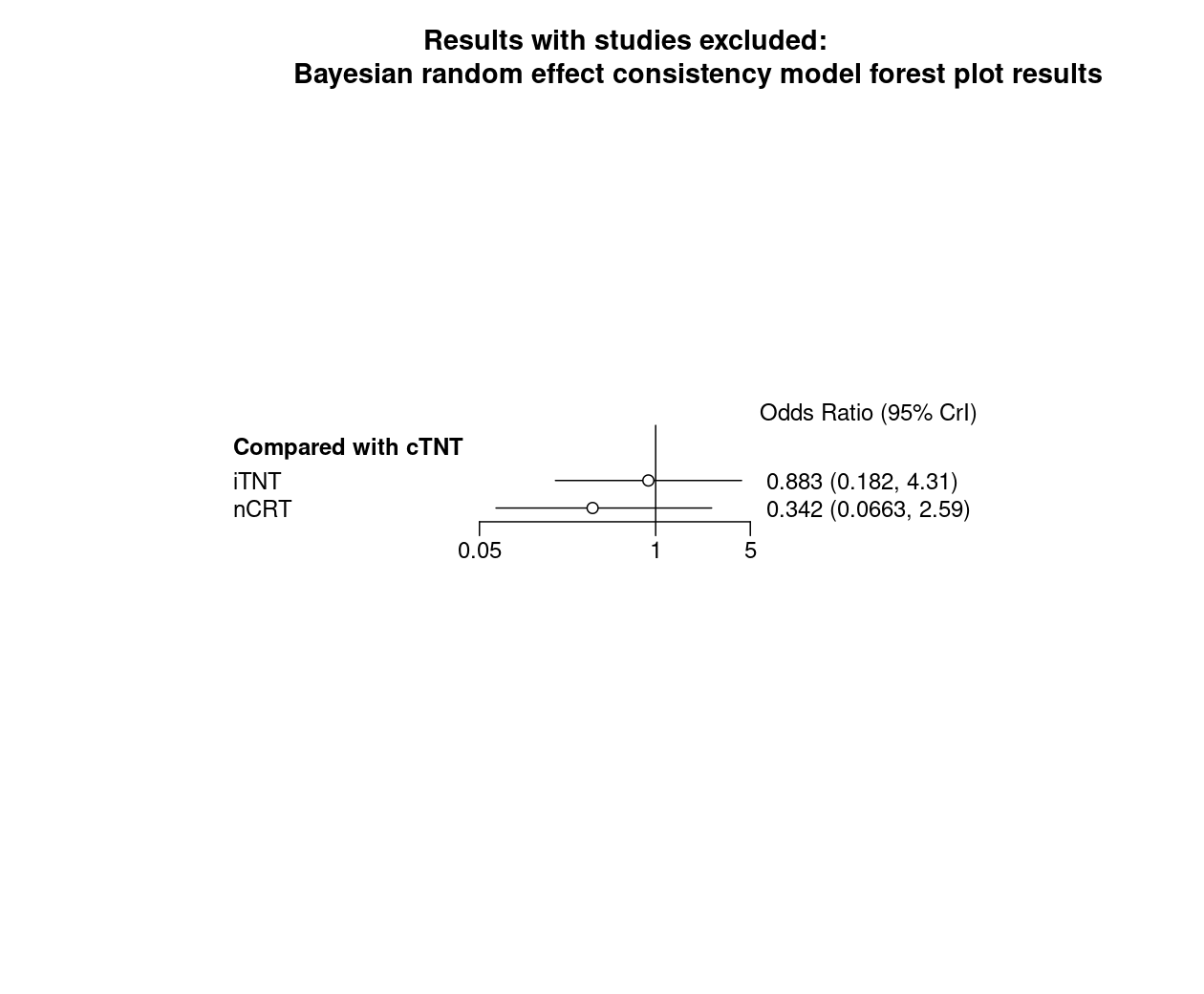 |
| Compliance to short-course radiotherapy | 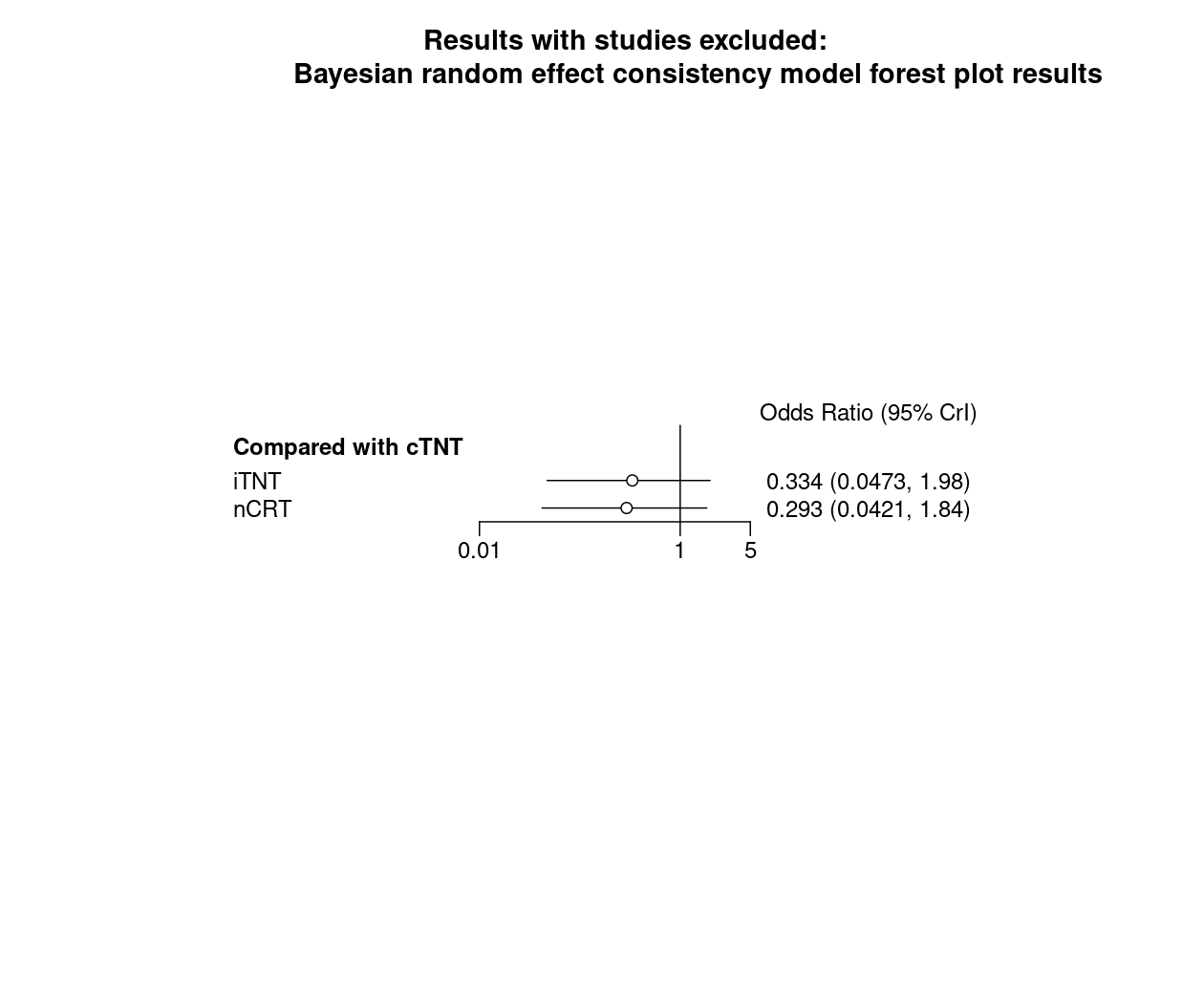 |
| Overall toxicity | 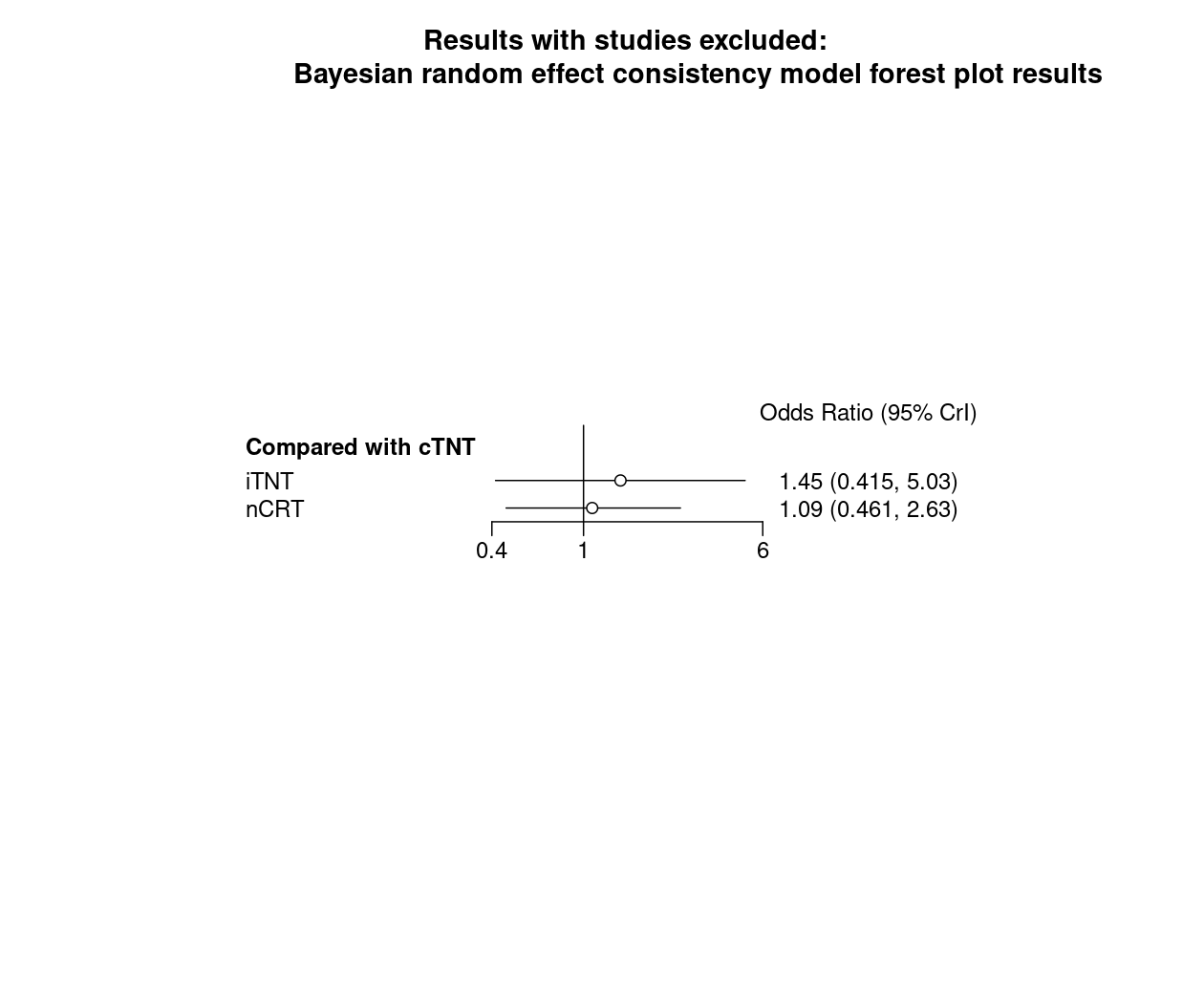 |
| Postoperative complications | 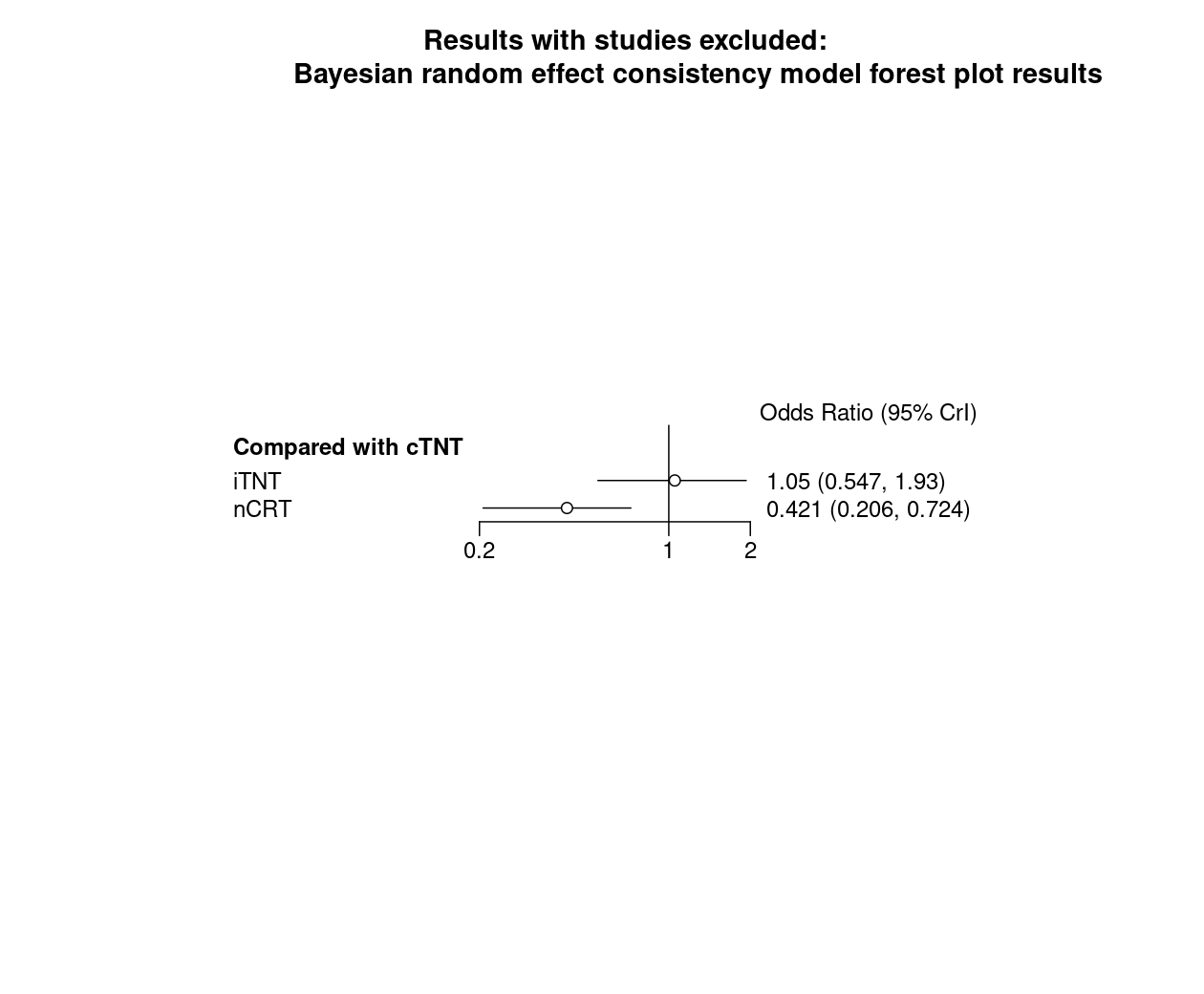 |

*Treatment regimens consisting of short-course radiotherapy (i.e. 25Gy) excluded from pooled studies

** Treatment regimens consisting of long-course radiotherapy (i.e. ≥50Gy) excluded from pooled studies

Neoadjuvant chemoradiation agents (Oral capecitabine vs. infusional 5-FU)

| **Oral capecitabine** | |
| --- | --- |
| Compliance to chemotherapy | 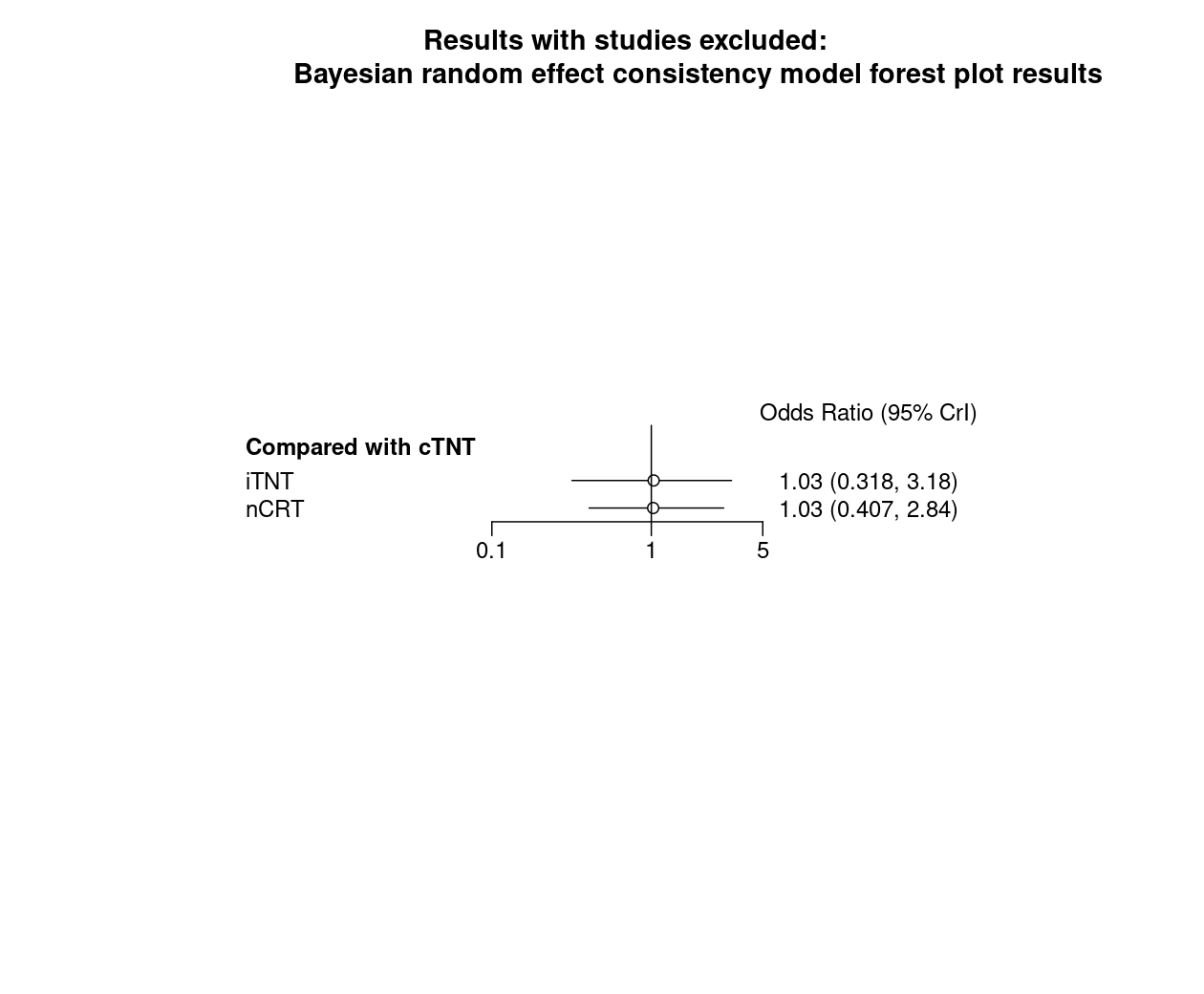 |
| Compliance to radiotherapy | 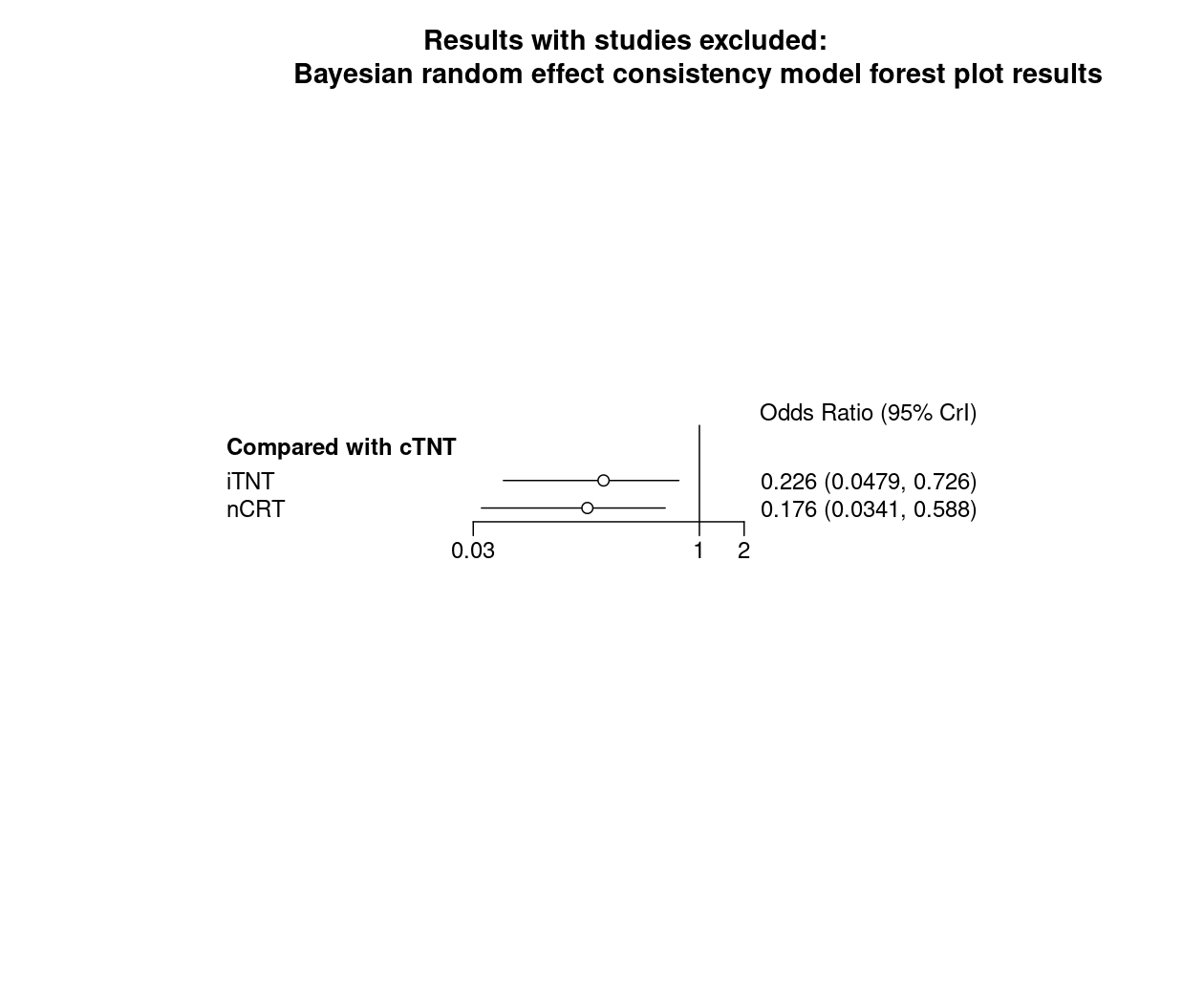 |
| Overall toxicity | 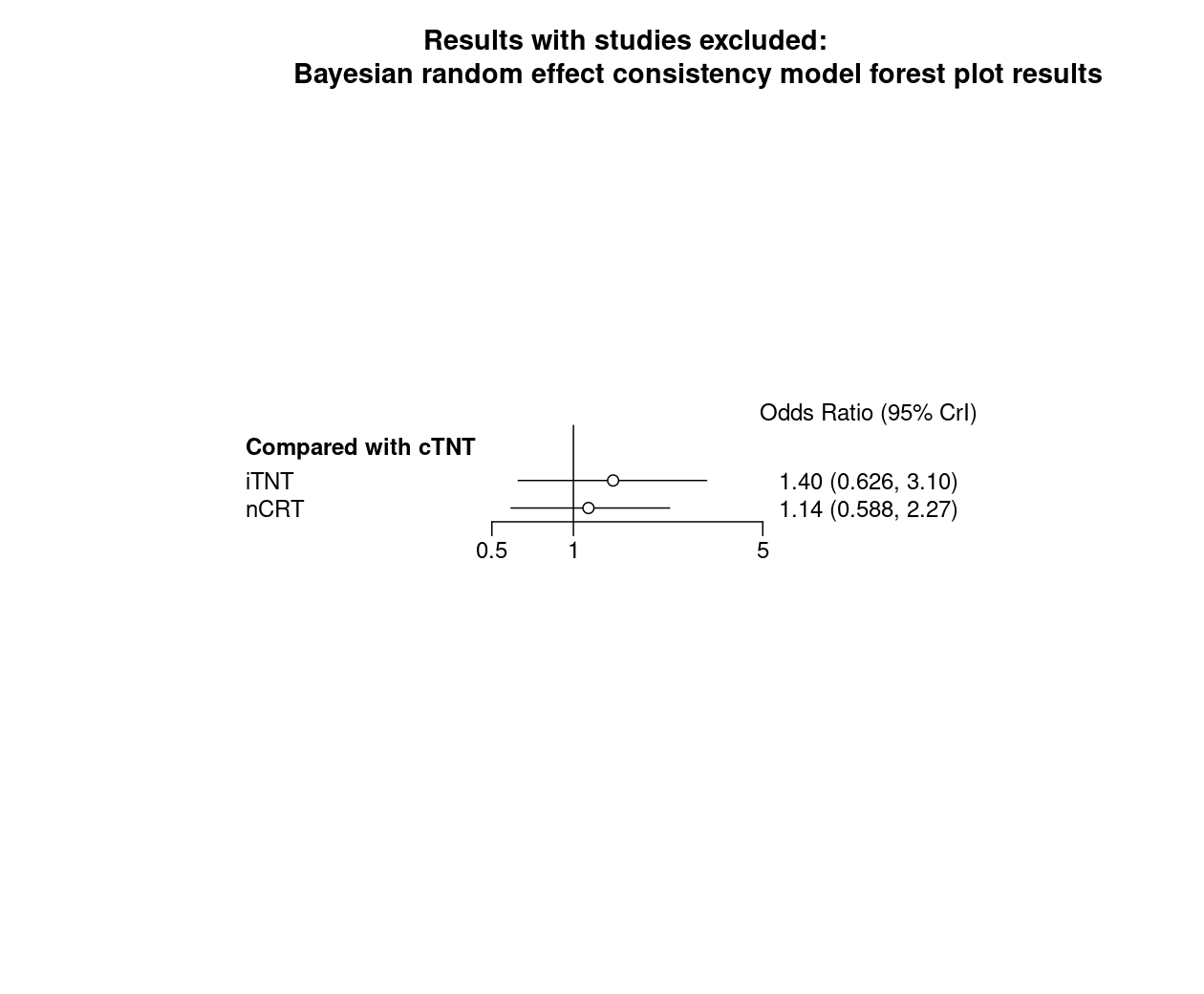 |
| Postoperative complications | 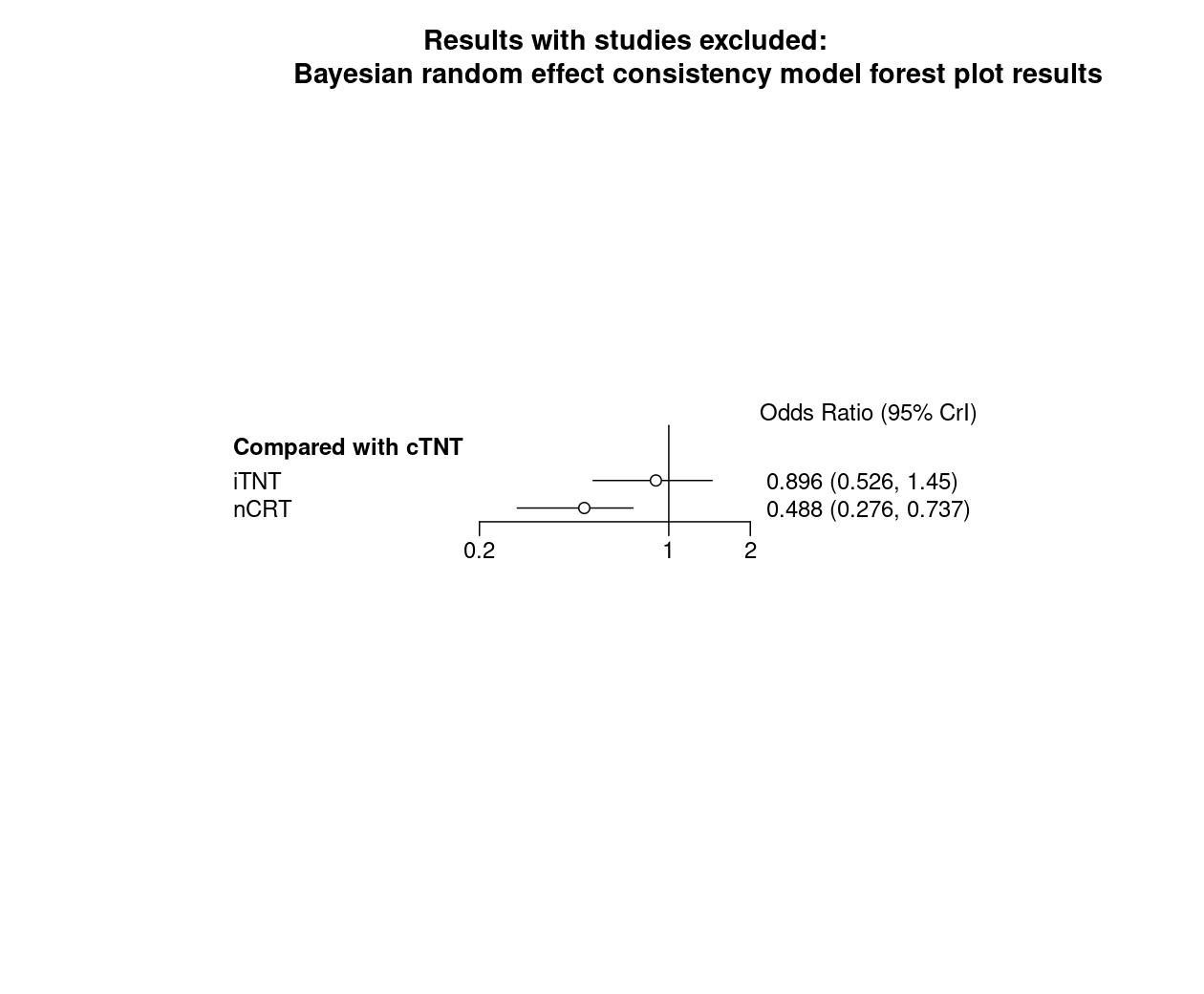 |
| **Infusional 5-FU** | |
| Compliance to chemotherapy | 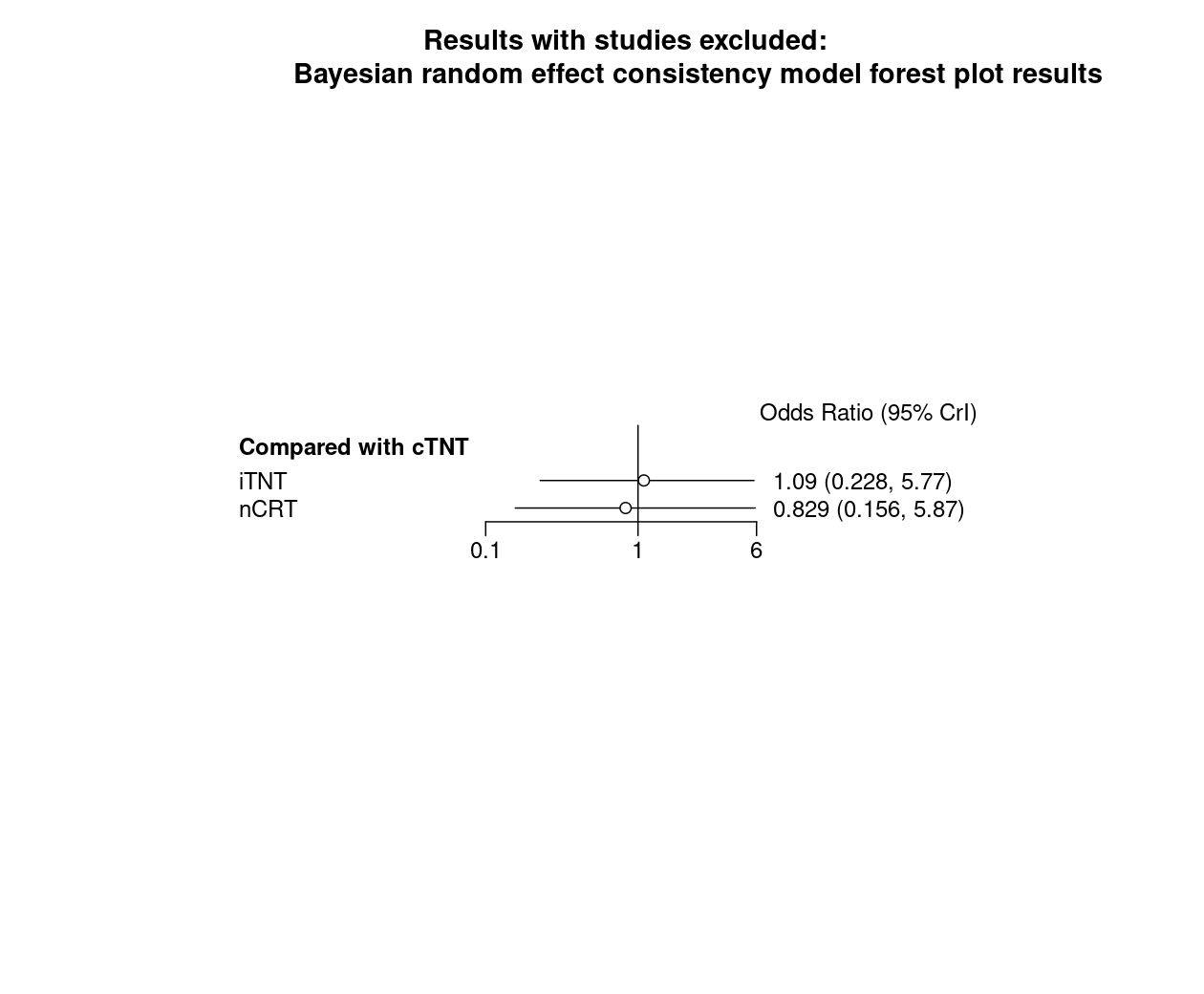 |
| Compliance to radiotherapy | 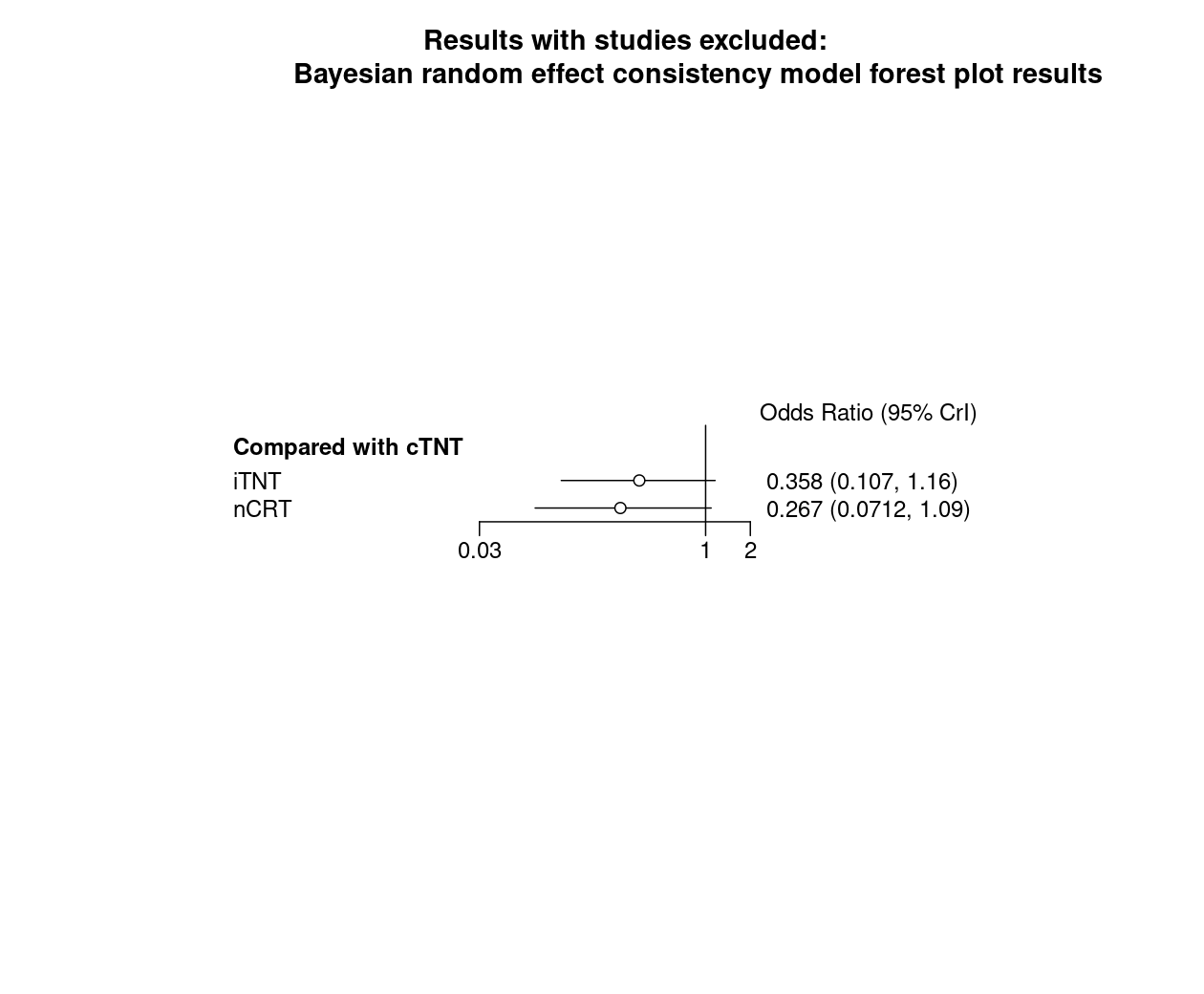 |
| Overall toxicity | 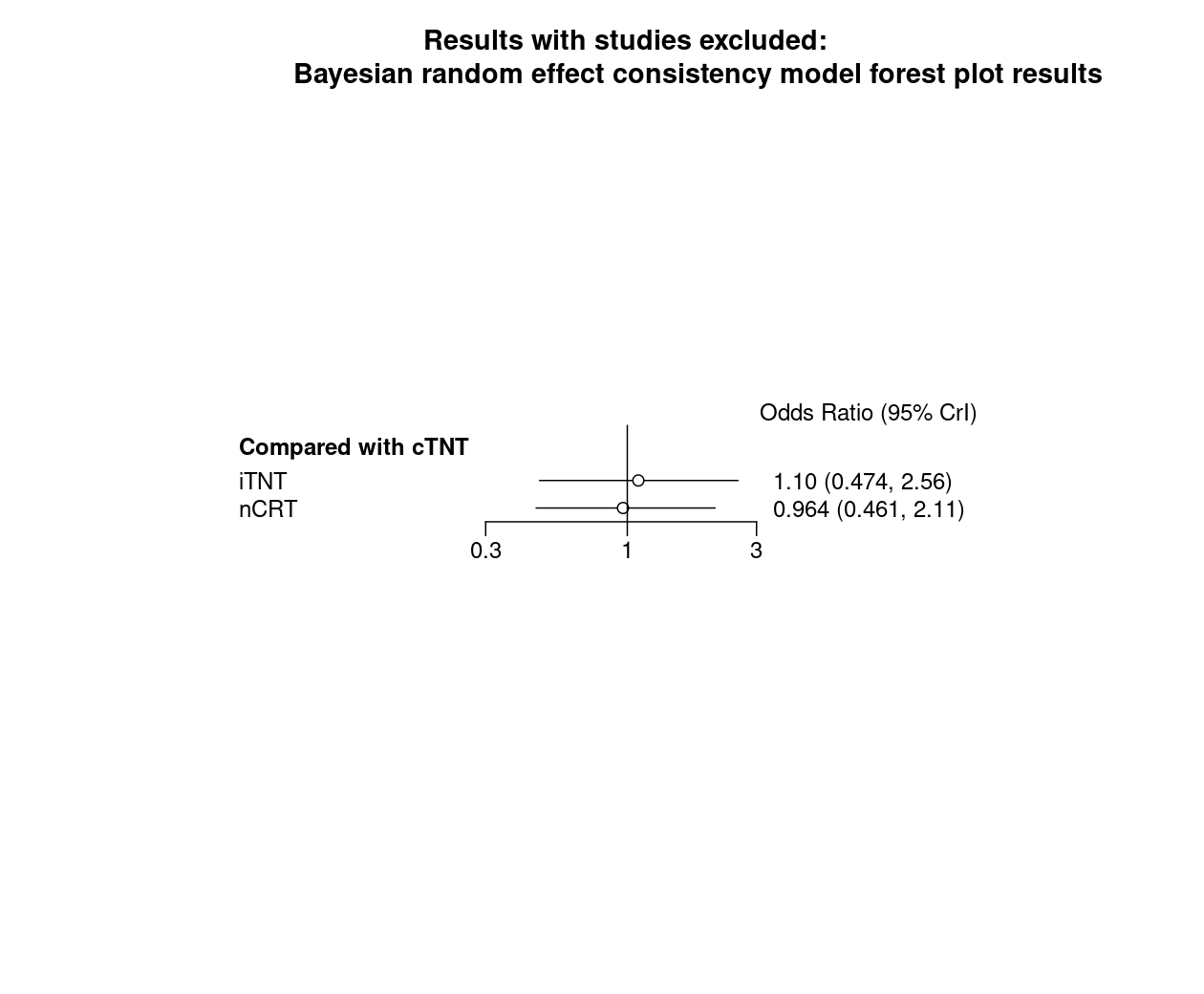 |
| Postoperative complications | 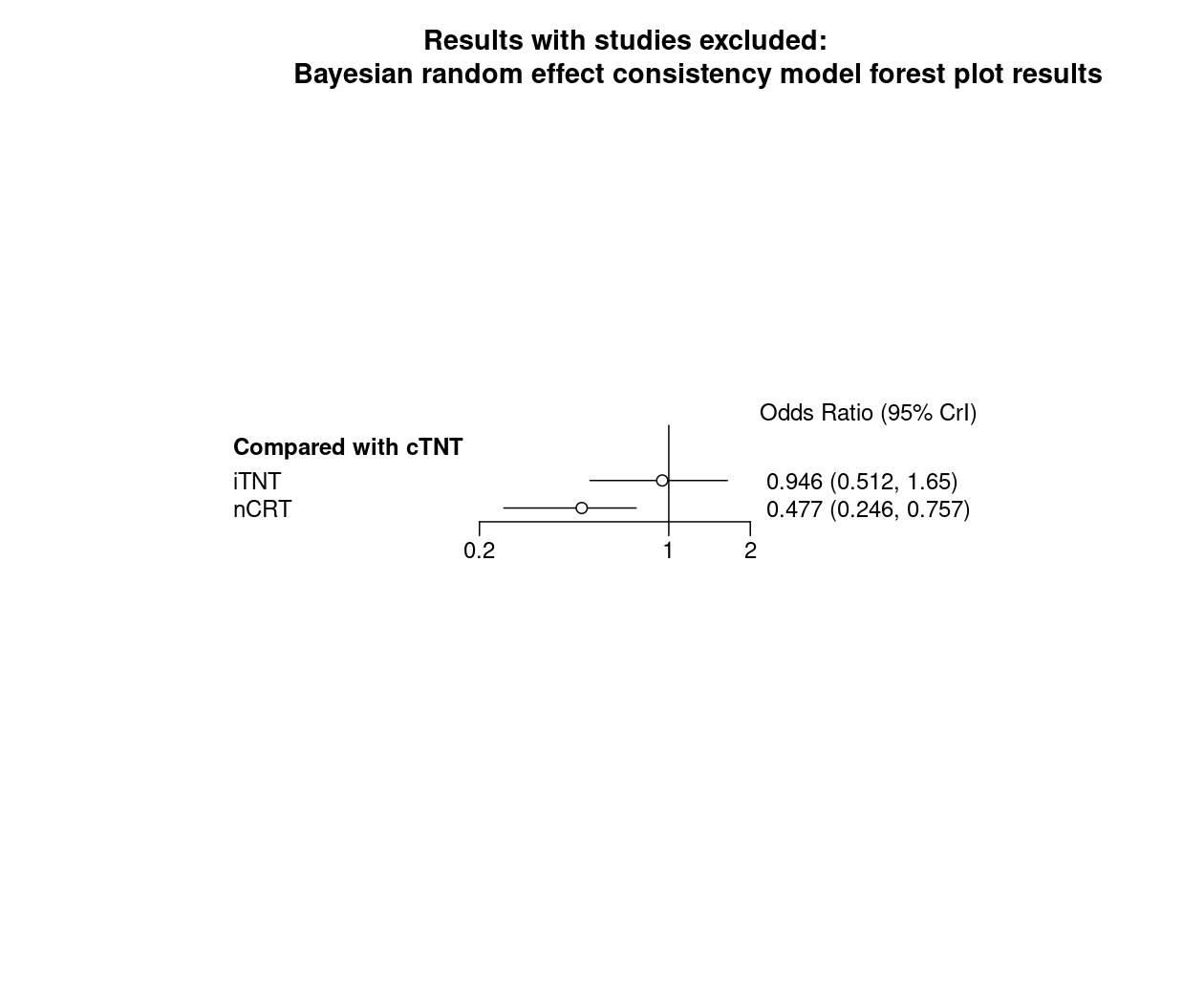 |

## Appendix 10: Node-splitting analysis for inconsistencies across treatment comparisons

**Toxicity outcomes**

Overall Grade III and above treatment-related adverse events

| **Direct comparison** | **Direct Estimate**  **(95% CrI)** | **Indirect Estimate**  **(95% CrI)** | **Inconsistency**  **(95% CrI)** | **Inconsistency P-value** |
| --- | --- | --- | --- | --- |
| cTNT - iTNT | 0.273 (-1.268, 1.848) | 0.285 (-0.742, 1.298) | -0.014 (-1.889, 1.882) | 0.988 |
| cTNT - nCRT | 0.168 (-0.605, 0.984) | 0.150 (-1.548, 1.864) | 0.023 (-1.818, 1.887) | 0.611 |
| iTNT - nCRT | -0.124 (-0.780, 0.526) | -0.094 (-1.843, 1.723) | -0.024 (-1.929, 1.808) | 0.977 |

Treatment-related mortality

| **Direct comparison** | **Direct Estimate**  **(95% CrI)** | **Indirect Estimate**  **(95% CrI)** | **Inconsistency**  **(95% CrI)** | **Inconsistency P-value** |
| --- | --- | --- | --- | --- |
| cTNT - iTNT | -0.064 (-1.563, 1.328) | -0.227 (-1.911, 1.480) | 0.180 (-2.115, 2.325) | 0.884 |
| cTNT - nCRT | 0.021 (-1.204, 1.280) | 0.049 (-1.877, 1.966) | -0.033 (-2.286, 2.173) | 0.977 |
| iTNT - nCRT | 0.195 (-1.119, 1.359) | 0.163 (-1.755, 1.937) | 0.029 (-2.148, 2.284) | 0.978 |

**Individual Grade III and above treatment-related adverse events**

Diarrhoea

| **Direct comparison** | **Direct Estimate**  **(95% CrI)** | **Indirect Estimate**  **(95% CrI)** | **Inconsistency**  **(95% CrI)** | **Inconsistency P-value** |
| --- | --- | --- | --- | --- |
| cTNT - iTNT | 0.109 (-0.925, 1.464) | -0.575 (-1.322, 0.248) | 0.679 (-0.644, 2.228) | 0.278 |
| cTNT - nCRT | -0.524 (-1.037, 0.065) | 0.125 (-1.001, 1.711) | -0.651 (-2.262, 0.585) | 0.294 |
| iTNT - nCRT | 0.035 (-0.539, 0.660) | -0.646 (-2.122, 0.563) | 0.686 (-0.633, 2.314) | 0.273 |

Mucositis

| **Direct comparison** | **Direct Estimate**  **(95% CrI)** | **Indirect Estimate**  **(95% CrI)** | **Inconsistency**  **(95% CrI)** | **Inconsistency P-value** |
| --- | --- | --- | --- | --- |
| cTNT - iTNT | 0.062 (-4.464, 4.451) | -18.806 (-73.444, -1.780) | 18.857 (0.892, 73.348) | **0.032** |
| cTNT - nCRT | -22.461 (-71.548, -1.831) | 0.589 (-4.141, 5.825) | -23.231 (-72.049, -1.386) | **0.026** |
| iTNT - nCRT | 0.547 (-1.509, 3.484) | -19.207 (-59.440, -0.476) | 19.919 (1.059, 59.981) | **0.033** |

Oesophagitis

| **Direct comparison** | **Direct Estimate**  **(95% CrI)** | **Indirect Estimate**  **(95% CrI)** | **Inconsistency**  **(95% CrI)** | **Inconsistency P-value** |
| --- | --- | --- | --- | --- |
| cTNT - iTNT | -1.004 (-2.674, 0.541) | -1.143 (-5.579, 3.329) | 0.130 (-4.633, 4.812) | 0.949 |
| cTNT - nCRT | -2.094 (-5.737, 0.442) | -2.005 (-6.095, 1.309) | -0.157 (-5.033, 4.713) | 0.948 |
| iTNT - nCRT | -0.987 (-4.529, 1.944) | -1.114 (-5.116, 1.803) | 0.150 (-4.468, 5.119) | 0.942 |

Enterocolitis

| **Direct comparison** | **Direct Estimate**  **(95% CrI)** | **Indirect Estimate**  **(95% CrI)** | **Inconsistency**  **(95% CrI)** | **Inconsistency P-value** |
| --- | --- | --- | --- | --- |
| cTNT - iTNT | -1.235 (-3.306, 0.485) | -0.785 (-3.224, 1.956) | -0.473 (-3.824, 2.551) | 0.751 |
| cTNT - nCRT | -1.261 (-2.886, 0.315) | -1.767 (-4.724, 0.740) | 0.521 (-2.557, 3.919) | 0.732 |
| iTNT - nCRT | -0.517 (-2.650, 1.479) | -0.046 (-2.451, 2.540) | -0.502 (-3.868, 2.549) | 0.729 |

Vomiting

| **Direct comparison** | **Direct Estimate**  **(95% CrI)** | **Indirect Estimate**  **(95% CrI)** | **Inconsistency**  **(95% CrI)** | **Inconsistency P-value** |
| --- | --- | --- | --- | --- |
| cTNT - iTNT | -2.347 (-4.765, -0.354) | -0.577 (-2.694, 1.464) | -1.785 (-4.906, 1.166) | 0.206 |
| cTNT - nCRT | -0.916 (-2.333, 0.272) | -2.718 (-5.646, -0.084) | 1.795 (-1.185, 4.913) | 0.206 |
| iTNT - nCRT | -0.377 (-1.979, 1.273) | 1.405 (-1.059, 4.100) | -1.797 (-4.966, 1.202) | 0.219 |

Nausea

| **Direct comparison** | **Direct Estimate**  **(95% CrI)** | **Indirect Estimate**  **(95% CrI)** | **Inconsistency**  **(95% CrI)** | **Inconsistency P-value** |
| --- | --- | --- | --- | --- |
| cTNT - iTNT | 0.503 (-1.800, 2.584) | -0.144 (-2.044, 1.564) | 0.679 (-2.210, 3.438) | 0.603 |
| cTNT - nCRT | -0.665 (-1.887, 0.469) | -0.022 (-2.618, 2.414) | -0.632 (-3.432, 2.157) | 0.621 |
| iTNT - nCRT | -0.535 (-1.739, 0.807) | -1.157 (-3.631, 1.483) | 0.629 (-2.231, 3.429) | 0.626 |

Bowel obstruction

| **Direct comparison** | **Direct Estimate**  **(95% CrI)** | **Indirect Estimate**  **(95% CrI)** | **Inconsistency**  **(95% CrI)** | **Inconsistency P-value** |
| --- | --- | --- | --- | --- |
| cTNT - iTNT | 0.620 (-1.502, 2.911) | -0.020 (-2.756, 2.768) | 0.616 (-2.799, 4.241) | 0.721 |
| cTNT - nCRT | -1.185 (-3.424, 0.809) | -0.625 (-3.481, 2.147) | -0.595 (-4.209, 2.890) | 0.734 |
| iTNT - nCRT | -1.244 (-3.141, 0.488) | -1.853 (-5.178, 1.157) | 0.594 (-2.969, 4.495) | 0.739 |

Pancreatitis

| **Direct comparison** | **Direct Estimate**  **(95% CrI)** | **Indirect Estimate**  **(95% CrI)** | **Inconsistency**  **(95% CrI)** | **Inconsistency P-value** |
| --- | --- | --- | --- | --- |
| cTNT - iTNT | 13.070 (1.178, 35.518) | 1.468 (0.366, 2.751) | 11.473 (-0.386, 34.058) | 0.064 |
| cTNT - nCRT | 0.007 (-0.495, 0.521) | 13.011 (0.423, 33.356) | -13.004 (-33.271, -0.382) | 0.071 |
| iTNT – nCRT | -1.465 (-2.677, -0.491) | -11.292 (-30.741, -1.576) | 9.827 (0.094, 29.411) | 0.069 |

Proctitis

| **Direct comparison** | **Direct Estimate**  **(95% CrI)** | **Indirect Estimate**  **(95% CrI)** | **Inconsistency**  **(95% CrI)** | **Inconsistency P-value** |
| --- | --- | --- | --- | --- |
| cTNT - iTNT | -0.747 (-2.901, 1.079) | -1.454 (-3.304, 0.329) | 0.715 (-1.985, 3.354) | 0.595 |
| cTNT - nCRT | -1.024 (-2.236, 0.100) | -0.303 (-2.819, 2.104) | -0.710 (-3.371, 2.017) | 0.595 |
| iTNT - nCRT | 0.462 (-0.988, 1.954) | -0.265 (-2.442, 1.975) | 0.739 (-2.027, 3.417) | 0.599 |

Rectal bleeding

| **Direct comparison** | **Direct Estimate**  **(95% CrI)** | **Indirect Estimate**  **(95% CrI)** | **Inconsistency**  **(95% CrI)** | **Inconsistency P-value** |
| --- | --- | --- | --- | --- |
| cTNT - iTNT | 0.524 (-1.581, 2.937) | 2.001 (0.250, 4.124) | -1.453 (-4.472, 1.470) | 0.315 |
| cTNT - nCRT | 0.570 (-0.564, 1.802) | -0.894 (-3.755, 1.819) | 1.503 (-1.428, 4.594) | 0.317 |
| iTNT - nCRT | -1.475 (-3.169, -0.152) | 0.044 (-2.455, 2.563) | -1.518 (-4.403, 1.269) | 0.295 |

Dizziness

| **Direct comparison** | **Direct Estimate**  **(95% CrI)** | **Indirect Estimate**  **(95% CrI)** | **Inconsistency**  **(95% CrI)** | **Inconsistency P-value** |
| --- | --- | --- | --- | --- |
| cTNT - iTNT | 0.183 (-1.373, 1.664) | -1.377 (-4.036, 1.142) | 1.535 (-1.414, 4.563) | 0.280 |
| cTNT - nCRT | -0.697 (-1.915, 0.406) | 0.762 (-1.887, 3.394) | -1.486 (-4.402, 1.328) | 0.318 |
| iTNT - nCRT | 0.621 (-1.540, 2.876) | -0.852 (-2.804, 1.051) | 1.517 (-1.362, 4.434) | 0.308 |

Dysarthria

| **Direct comparison** | **Direct Estimate**  **(95% CrI)** | **Indirect Estimate**  **(95% CrI)** | **Inconsistency**  **(95% CrI)** | **Inconsistency P-value** |
| --- | --- | --- | --- | --- |
| cTNT - iTNT | 0.554 (-0.762, 1.930) | -0.003 (-3.075, 3.199) | 0.558 (-2.896, 3.807) | 0.732 |
| cTNT - nCRT | -0.087 (-2.426, 2.115) | 0.394 (-2.255, 2.794) | -0.484 (-3.965, 2.926) | 0.784 |
| iTNT - nCRT | -0.035 (-2.308, 2.066) | -0.673 (-3.156, 1.866) | 0.588 (-2.772, 3.983) | 0.718 |

Hand-foot syndrome

| **Direct comparison** | **Direct Estimate**  **(95% CrI)** | **Indirect Estimate**  **(95% CrI)** | **Inconsistency**  **(95% CrI)** | **Inconsistency P-value** |
| --- | --- | --- | --- | --- |
| cTNT - iTNT | 1.760 (0.140, 3.875) | -1.265 (-2.888, 0.214) | 3.049 (0.866, 5.705) | 0.007 |
| cTNT - nCRT | -0.671 (-1.560, 0.126) | 2.291 (0.252, 4.806) | -2.978 (-5.532, -0.737) | 0.008 |
| iTNT - nCRT | 0.591 (-0.687, 1.871) | -2.377 (-4.561, -0.583) | 2.994 (0.734, 5.545) | 0.011 |

Neuropathy

| **Direct comparison** | **Direct Estimate**  **(95% CrI)** | **Indirect Estimate**  **(95% CrI)** | **Inconsistency**  **(95% CrI)** | **Inconsistency P-value** |
| --- | --- | --- | --- | --- |
| cTNT - iTNT | 0.167 (-2.399, 2.886) | -3.013 (-7.177, 0.038) | 3.231 (-0.791, 8.161) | 0.115 |
| cTNT - nCRT | -3.362 (-7.154, -0.766) | -0.272 (-3.270, 2.904) | -3.171 (-8.032, 0.807) | 0.118 |
| iTNT - nCRT | -0.379 (-2.056, 1.146) | -3.662 (-8.448, -0.007) | 3.297 (-0.756, 8.307) | 0.109 |

Syncope

| **Direct comparison** | **Direct Estimate**  **(95% CrI)** | **Indirect Estimate**  **(95% CrI)** | **Inconsistency**  **(95% CrI)** | **Inconsistency P-value** |
| --- | --- | --- | --- | --- |
| cTNT - iTNT | 1.230 (-0.484, 3.180) | -1.656 (-4.231, 0.965) | 2.902 (-0.246, 6.040) | 0.070 |
| cTNT - nCRT | -0.934 (-2.027, 0.089) | 1.851 (-1.040, 4.778) | -2.781 (-5.970, 0.338) | 0.080 |
| iTNT - nCRT | 0.681 (-1.648, 2.916) | -2.122 (-4.429, -0.135) | 2.826 (-0.281, 5.899) | 0.076 |

Musculoskeletal weakness

| **Direct comparison** | **Direct Estimate**  **(95% CrI)** | **Indirect Estimate**  **(95% CrI)** | **Inconsistency**  **(95% CrI)** | **Inconsistency P-value** |
| --- | --- | --- | --- | --- |
| cTNT - iTNT | 0.128 (-1.164, 1.399) | -0.785 (-1.955, 0.190) | 0.923 (-0.685, 2.652) | 0.247 |
| cTNT - nCRT | -0.847 (-1.771, 0.039) | 0.063 (-1.230, 1.514) | -0.905 (-2.600, 0.640) | 0.250 |
| iTNT - nCRT | -0.071 (-0.541, 0.653) | -0.999 (-2.564, 0.533) | 0.947 (-0.638, 2.671) | 0.226 |

Arrythmia

| **Direct comparison** | **Direct Estimate**  **(95% CrI)** | **Indirect Estimate**  **(95% CrI)** | **Inconsistency**  **(95% CrI)** | **Inconsistency P-value** |
| --- | --- | --- | --- | --- |
| cTNT - iTNT | -0.407 (-1.552, 0.938) | 0.581 (-1.124, 2.213) | -0.989 (-2.895, 1.303) | 0.338 |
| cTNT - nCRT | 0.272 (-0.883, 1.369) | -0.650 (-2.325, 1.123) | 0.931 (-1.268, 2.896) | 0.348 |
| iTNT - nCRT | -0.264 (-1.574, 0.928) | 0.717 (-1.153, 2.258) | -0.984 (-2.977, 1.286) | 0.344 |

Venous thromboembolism

| **Direct comparison** | **Direct Estimate**  **(95% CrI)** | **Indirect Estimate**  **(95% CrI)** | **Inconsistency**  **(95% CrI)** | **Inconsistency P-value** |
| --- | --- | --- | --- | --- |
| cTNT - iTNT | 0.550 (-0.572, 1.761) | 0.490 (-1.377, 2.697) | 0.043 (-2.404, 2.267) | 0.971 |
| cTNT - nCRT | -1.046 (-1.878, -0.348) | -1.020 (-3.376, 1.142) | -0.033 (-2.275, 2.418) | 0.981 |
| iTNT - nCRT | -1.546 (-3.715, 0.073) | -1.619 (-3.117, -0.251) | 0.044 (-2.449, 2.251) | 0.964 |

Pneumonia

| **Direct comparison** | **Direct Estimate**  **(95% CrI)** | **Indirect Estimate**  **(95% CrI)** | **Inconsistency**  **(95% CrI)** | **Inconsistency P-value** |
| --- | --- | --- | --- | --- |
| cTNT - iTNT | 1.696 (0.073, 3.981) | -1.858 (-4.758, 0.966) | 3.643 (0.270, 7.229) | **0.035** |
| cTNT - nCRT | -1.159 (-2.762, 0.286) | 2.517 (-0.394, 5.800) | -3.697 (-7.313, -0.400) | **0.028** |
| iTNT - nCRT | 0.697 (-1.747, 3.035) | -2.890 (-5.692, -0.694) | 3.642 (0.417, 7.176) | **0.029** |

Urinary tract infection

| **Direct comparison** | **Direct Estimate**  **(95% CrI)** | **Indirect Estimate**  **(95% CrI)** | **Inconsistency**  **(95% CrI)** | **Inconsistency P-value** |
| --- | --- | --- | --- | --- |
| cTNT - iTNT | -0.460 (-1.666, 0.670) | -0.044 (-1.955, 1.773) | -0.403 (-2.642, 1.798) | 0.694 |
| cTNT - nCRT | -0.778 (-2.424, 0.662) | -1.169 (-2.802, 0.318) | 0.390 (-1.808, 2.610) | 0.727 |
| iTNT - nCRT | -0.729 (-1.828, 0.296) | -0.348 (-2.420, 1.438) | -0.385 (-2.509, 1.915) | 0.740 |

Sepsis

| **Direct comparison** | **Direct Estimate**  **(95% CrI)** | **Indirect Estimate**  **(95% CrI)** | **Inconsistency**  **(95% CrI)** | **Inconsistency P-value** |
| --- | --- | --- | --- | --- |
| cTNT - iTNT | -0.745 (-4.169, 1.789) | -1.321 (-3.868, 1.159) | 0.531 (-3.547, 4.107) | 0.778 |
| cTNT - nCRT | -0.539 (-2.395, 1.146) | -0.029 (-3.832, 3.107) | -0.499 (-4.063, 3.711) | 0.793 |
| iTNT - nCRT | 0.713 (-1.048, 2.492) | 0.214 (-3.064, 4.329) | 0.514 (-4.007, 4.244) | 0.803 |

Radiation dermatitis

| **Direct comparison** | **Direct Estimate**  **(95% CrI)** | **Indirect Estimate**  **(95% CrI)** | **Inconsistency**  **(95% CrI)** | **Inconsistency P-value** |
| --- | --- | --- | --- | --- |
| cTNT - iTNT | -0.439 (-2.526, 1.644) | 1.113 (-1.655, 3.620) | -1.545 (-4.784, 1.999) | 0.305 |
| cTNT - nCRT | 0.782 (-0.675, 2.095) | -0.748 (-3.883, 2.363) | 1.554 (-2.028, 4.780) | 0.317 |
| iTNT - nCRT | -0.296 (-2.550, 2.018) | 1.280 (-1.419, 3.685) | -1.616 (-4.808, 1.986) | 0.307 |

Renal and electrolyte imbalances

| **Direct comparison** | **Direct Estimate**  **(95% CrI)** | **Indirect Estimate**  **(95% CrI)** | **Inconsistency**  **(95% CrI)** | **Inconsistency P-value** |
| --- | --- | --- | --- | --- |
| cTNT - iTNT | -0.090 (-0.952, 0.827) | -1.875 (-4.317, -0.009) | 1.816 (-0.279, 4.442) | 0.088 |
| cTNT - nCRT | 0.004 (-0.925, 0.834) | 1.713 (-0.063, 3.935) | -1.729 (-4.129, 0.249) | 0.086 |
| iTNT - nCRT | 1.865 (0.291, 4.072) | 0.101 (-1.201, 1.310) | 1.781 (-0.245, 4.327) | 0.088 |

Febrile neutropaenia

| **Direct comparison** | **Direct Estimate**  **(95% CrI)** | **Indirect Estimate**  **(95% CrI)** | **Inconsistency**  **(95% CrI)** | **Inconsistency P-value** |
| --- | --- | --- | --- | --- |
| cTNT - iTNT | -1.924 (-5.566, 0.255) | 0.291 (-1.929, 2.381) | -2.271 (-6.217, 0.962) | 0.187 |
| cTNT - nCRT | -0.946 (-2.789, 0.604) | -3.118 (-6.585, -0.507) | 2.163 (-0.901, 5.974) | 0.175 |
| iTNT - nCRT | -1.197 (-2.683, -0.003) | 0.952 (-1.836, 4.679) | -2.182 (-6.143, 0.937) | 0.160 |

Neutropaenia

| **Direct comparison** | **Direct Estimate**  **(95% CrI)** | **Indirect Estimate**  **(95% CrI)** | **Inconsistency**  **(95% CrI)** | **Inconsistency P-value** |
| --- | --- | --- | --- | --- |
| cTNT - iTNT | -0.741 (-4.177, 1.636) | -0.708 (-3.245, 1.827) | -0.044 (-4.494, 3.258) | 0.978 |
| cTNT - nCRT | -2.320 (-4.558, -0.618) | -2.314 (-6.493, 0.269) | 0.012 (-3.290, 4.423) | 0.994 |
| iTNT - nCRT | -1.558 (-3.580, -0.297) | -1.559 (-4.716, 1.969) | 0.011 (-4.293, 3.229) | 0.995 |

Lymphopenia

| **Direct comparison** | **Direct Estimate**  **(95% CrI)** | **Indirect Estimate**  **(95% CrI)** | **Inconsistency**  **(95% CrI)** | **Inconsistency P-value** |
| --- | --- | --- | --- | --- |
| cTNT - iTNT | -0.912 (-1.584, -0.279) | 0.019 (-0.803, 0.875) | -0.933 (-2.046, 0.129) | 0.079 |
| cTNT - nCRT | -0.029 (-0.750, 0.729) | -0.953 (-1.767, -0.163) | 0.912 (-0.114, 2.018) | 0.083 |
| iTNT - nCRT | -0.035 (-0.504, 0.386) | 0.882 (-0.102, 1.837) | -0.922 (-1.969, 0.137) | 0.079 |

Thrombocytopaenia

| **Direct comparison** | **Direct Estimate**  **(95% CrI)** | **Indirect Estimate**  **(95% CrI)** | **Inconsistency**  **(95% CrI)** | **Inconsistency P-value** |
| --- | --- | --- | --- | --- |
| cTNT - iTNT | 0.312 (-2.574, 3.324) | -0.603 (-3.055, 2.240) | 0.947 (-3.243, 4.664) | 0.618 |
| cTNT - nCRT | -0.793 (-2.242, 1.004) | 0.187 (-3.416, 3.638) | -0.953 (-4.597, 3.118) | 0.610 |
| iTNT - nCRT | -0.163 (-2.219, 1.817) | -1.152 (-4.229, 2.311) | 0.981 (-3.080, 4.601) | 0.596 |

Anaemia

| **Direct comparison** | **Direct Estimate**  **(95% CrI)** | **Indirect Estimate**  **(95% CrI)** | **Inconsistency**  **(95% CrI)** | **Inconsistency P-value** |
| --- | --- | --- | --- | --- |
| cTNT - iTNT | 0.339 (-2.193, 2.664) | -0.726 (-2.655, 1.211) | 1.023 (-2.055, 3.957) | 0.479 |
| cTNT - nCRT | -1.247 (-2.751, -0.003) | -0.187 (-2.989, 2.434) | -1.085 (-4.096, 2.068) | 0.474 |
| iTNT - nCRT | -0.518 (-1.938, 0.779) | -1.661 (-4.499, 1.112) | 1.154 (-2.060, 4.236) | 0.455 |

Anaphylaxis

| **Direct comparison** | **Direct Estimate**  **(95% CrI)** | **Indirect Estimate**  **(95% CrI)** | **Inconsistency**  **(95% CrI)** | **Inconsistency P-value** |
| --- | --- | --- | --- | --- |
| cTNT - iTNT | 0.826 (-1.042, 2.918) | -0.770 (-3.158, 1.409) | 1.651 (-1.221, 4.790) | 0.274 |
| cTNT - nCRT | -1.174 (-3.277, 0.482) | 0.383 (-1.934, 2.949) | -1.606 (-4.842, 1.330) | 0.285 |
| iTNT - nCRT | -0.434 (-1.905, 0.864) | -2.079 (-5.069, 0.400) | 1.626 (-1.248, 4.905) | 0.268 |

**Compliance outcomes**

Compliance with radiotherapy

| **Direct comparison** | **Direct Estimate**  **(95% CrI)** | **Indirect Estimate**  **(95% CrI)** | **Inconsistency**  **(95% CrI)** | **Inconsistency P-value** |
| --- | --- | --- | --- | --- |
| cTNT - iTNT | -1.404 (-3.573, 0.484) | -1.840 (-4.792, 0.638) | 0.428 (-2.971, 3.837) | 0.744 |
| cTNT - nCRT | -2.057 (-4.743, 0.056) | -1.649 (-4.094, 0.394) | -0.426 (-3.804, 2.932) | 0.745 |
| iTNT - nCRT | -0.225 (-1.396, 0.942) | -0.683 (-3.763, 2.286) | 0.460 (-2.782, 3.640) | 0.735 |

Compliance with chemotherapy (overall)

| **Direct comparison** | **Direct Estimate**  **(95% CrI)** | **Indirect Estimate**  **(95% CrI)** | **Inconsistency**  **(95% CrI)** | **Inconsistency P-value** |
| --- | --- | --- | --- | --- |
| cTNT - iTNT | -0.116 (-1.919, 1.660) | -0.065 (-1.810, 1.547) | -0.074 (-2.459, 2.502) | 0.943 |
| cTNT - nCRT | 0.119 (-1.085, 1.356) | 0.039 (-2.076, 2.232) | 0.088 (-2.430, 2.552) | 0.936 |
| iTNT - nCRT | 0.166 (-0.962, 1.394) | 0.264 (-1.899, 2.428) | -0.100 (-2.520, 2.348) | 0.930 |

Compliance with FOLFOX-specific chemotherapy

| **Direct comparison** | **Direct Estimate**  **(95% CrI)** | **Indirect Estimate**  **(95% CrI)** | **Inconsistency**  **(95% CrI)** | **Inconsistency P-value** |
| --- | --- | --- | --- | --- |
| cTNT - iTNT | 0.191 (-2.294, 2.810) | 0.456 (-1.955, 2.771) | -0.261 (-3.582, 3.299) | 0.881 |
| cTNT - nCRT | 0.495 (-1.113, 2.262) | 0.242 (-2.797, 3.294) | 0.253 (-3.184, 3.735) | 0.880 |
| iTNT - nCRT | 0.031 (-1.490, 1.739) | 0.299 (-2.708, 3.399) | -0.235 (-3.616, 3.158) | 0.879 |

Compliance with CAPOX-specific chemotherapy

| **Direct comparison** | **Direct Estimate**  **(95% CrI)** | **Indirect Estimate**  **(95% CrI)** | **Inconsistency**  **(95% CrI)** | **Inconsistency P-value** |
| --- | --- | --- | --- | --- |
| cTNT - iTNT | -0.112 (-1.338, 1.110) | 1.308 (-0.496, 2.602) | -1.428 (-3.141, 0.788) | 0.177 |
| cTNT - nCRT | 0.319 (-0.649, 1.105) | -1.088 (-2.662, 0.899) | 1.402 (-0.903, 3.111) | 0.185 |
| iTNT - nCRT | -0.985 (-2.105, 0.496) | 0.421 (-1.197, 1.830) | -1.393 (-3.102, 0.824) | 0.186 |

**Postoperative outcomes**

Overall Clavien-Dindo Grade 3 and above postoperative complications

| **Direct comparison** | **Direct Estimate**  **(95% CrI)** | **Indirect Estimate**  **(95% CrI)** | **Inconsistency**  **(95% CrI)** | **Inconsistency P-value** |
| --- | --- | --- | --- | --- |
| cTNT - iTNT | -0.282 (-1.028, 0.379) | 0.344 (-0.469, 1.099) | -0.621 (-1.676, 0.445) | 0.202 |
| cTNT - nCRT | -0.620 (-1.310, -0.096) | -1.257 (-2.211, -0.456) | 0.634 (-0.415, 1.704) | 0.211 |
| iTNT - nCRT | -0.968 (-1.547, -0.461) | -0.342 (-1.325, 0.565) | -0.622 (-1.735, 0.456) | 0.220 |

Anastomotic leak

| **Direct comparison** | **Direct Estimate**  **(95% CrI)** | **Indirect Estimate**  **(95% CrI)** | **Inconsistency**  **(95% CrI)** | **Inconsistency P-value** |
| --- | --- | --- | --- | --- |
| cTNT - iTNT | 0.366 (-1.166, 1.910) | 0.455 (-0.981, 2.133) | -0.099 (-2.370, 1.939) | 0.921 |
| cTNT - nCRT | 0.481 (-0.549, 1.526) | 0.313 (-1.640, 2.246) | 0.186 (-1.995, 2.396) | 0.857 |
| iTNT - nCRT | 0.005 (-1.289, 1.098) | 0.182 (-1.696, 1.970) | -0.187 (-2.458, 1.980) | 0.863 |

High stoma output/diarrhoea

| **Direct comparison** | **Direct Estimate**  **(95% CrI)** | **Indirect Estimate**  **(95% CrI)** | **Inconsistency**  **(95% CrI)** | **Inconsistency P-value** |
| --- | --- | --- | --- | --- |
| cTNT - iTNT | 0.979 (0.066, 1.893) | 1.082 (-0.193, 2.517) | -0.104 (-1.823, 1.438) | 0.885 |
| cTNT - nCRT | -0.072 (-0.906, 0.789) | -0.224 (-1.605, 1.152) | 0.115 (-1.449, 1.748) | 0.879 |
| iTNT - nCRT | -1.145 (-2.284, -0.177) | -1.050 (-2.197, 0.186) | -0.102 (-1.815, 1.404) | 0.896 |

Postoperative bowel obstruction

| **Direct comparison** | **Direct Estimate**  **(95% CrI)** | **Indirect Estimate**  **(95% CrI)** | **Inconsistency**  **(95% CrI)** | **Inconsistency P-value** |
| --- | --- | --- | --- | --- |
| cTNT - iTNT | 0.322 (-0.372, 1.000) | -0.334 (-1.455, 0.754) | 0.660 (-0.624, 1.975) | 0.316 |
| cTNT - nCRT | 0.134 (-0.810, 1.111) | 0.776 (-0.106, 1.667) | -0.639 (-1.907, 0.652) | 0.319 |
| iTNT - nCRT | 0.467 (-0.054, 1.034) | -0.118 (-1.320, 1.026) | 0.576 (-0.661, 1.927) | 0.338 |

Organ/space surgical site infection

| **Direct comparison** | **Direct Estimate**  **(95% CrI)** | **Indirect Estimate**  **(95% CrI)** | **Inconsistency**  **(95% CrI)** | **Inconsistency P-value** |
| --- | --- | --- | --- | --- |
| cTNT - iTNT | 0.799 (-0.254, 1.870) | 0.042 (-1.281, 1.168) | 0.771 (-0.747, 2.455) | 0.323 |
| cTNT - nCRT | 0.461 (-0.169, 0.995) | 1.227 (-0.256, 2.805) | -0.774 (-2.472, 0.817) | 0.330 |
| iTNT - nCRT | 0.482 (-0.581, 1.604) | -0.323 (-1.548, 0.771) | 0.822 (-0.738, 2.420) | 0.282 |

Superficial incisional surgical site infection

| **Direct comparison** | **Direct Estimate**  **(95% CrI)** | **Indirect Estimate**  **(95% CrI)** | **Inconsistency**  **(95% CrI)** | **Inconsistency P-value** |
| --- | --- | --- | --- | --- |
| cTNT - iTNT | -1.260 (-3.587, 0.557) | 0.068 (-1.164, 1.067) | -1.339 (-3.781, 0.861) | 0.225 |
| cTNT - nCRT | 0.128 (-0.842, 0.831) | -1.206 (-3.480, 0.706) | 1.320 (-0.820, 3.745) | 0.221 |
| iTNT - nCRT | 0.061 (-0.680, 0.777) | 1.388 (-0.713, 3.651) | -1.336 (-3.740, 0.879) | 0.222 |

Urinary tract infection

| **Direct comparison** | **Direct Estimate**  **(95% CrI)** | **Indirect Estimate**  **(95% CrI)** | **Inconsistency**  **(95% CrI)** | **Inconsistency P-value** |
| --- | --- | --- | --- | --- |
| cTNT - iTNT | 0.335 (-0.746, 1.431) | 0.825 (-0.447, 2.284) | -0.504 (-2.301, 1.223) | 0.563 |
| cTNT - nCRT | 0.277 (-0.736, 1.340) | -0.190 (-1.629, 1.074) | 0.475 (-1.188, 2.273) | 0.574 |
| iTNT - nCRT | -0.546 (-1.439, 0.287) | -0.067 (-1.529, 1.459) | -0.482 (-2.233, 1.218) | 0.565 |

Direct estimate is an estimate of the relative effect of one treatment compared with another, based solely on studies that include both treatments. Indirect estimate is an estimate of the relative effect of one treatment compared with another based solely on studies that do not include both treatments. The difference between the direct and the indirect estimate is termed the inconsistency factor. An inconsistency p-value <0.05 indicates inconsistencies within the network for the outcome measured.

## Appendix 11: Transitivity analysis

| **Treatment approach** | **Median RT dose, Gy (IQR)** | **CT subtypes (n, %)** | **CT cycles (SD)** | **Time interval, weeks^a^ (SD)** | **Median Age (IQR)** | **Percentage female (SD)** | **ECOG status (%)** | **Distance from anal verge, cm (SD)** | **Proceed to surgery (%)** | **Follow-up period, in months (SD)** |
| --- | --- | --- | --- | --- | --- | --- | --- | --- | --- | --- |
| cTNT | 50  (25.0-54) | FOLFOX (9, 64%)  CAPOX (5, 36%) | 5.8 (1.8) | 3.1 (1.0) | 55 (38–70) | 52.3 (20.3) | 0: 85  1: 18 | 6.0 (2.9) | 1167 (59) | 40 (8.1) |
| iTNT | 50.4  (50.2-54.0) | FOLFOX (11, 58%)  CAPOX  (7, 37%)  FOLFIRINOX (1, 5%) | 5.8 (1.5) | 2.8 (1.2) | 55 (39–72) | 53.6 (21.2) | 0: 82  1: 18 | 6.1 (2.2) | 712 (58) | 43 (6.8) |
| nCRT | 50.4 | - | - | - | 56 (36–72) | 52.2 (20.7) | 0: 85  1: 15 | 6.0 (3.1) | 1528 (60) | 44 (7.9) |

**RT**: Radiotherapy

**Gy**: gray, SI unit

**CT**: Chemotherapy

**ECOG**: Eastern Cooperative Oncology Group (ECOG)

**a**: Time interval between first and second treatment sequence

## Appendix 12: GRADE assessment Summary of Findings of all outcomes

*Toxicity outcomes*

|  | **Certainty assessment** | | | | | | | | | | | | | **№ of patients** | | **Certainty** |  |
| --- | --- | --- | --- | --- | --- | --- | --- | --- | --- | --- | --- | --- | --- | --- | --- | --- | --- |
| **Direct comparisons** | **№ of studies** | **Study design** | | **Risk of bias** | | **Inconsistency** | | **Indirectness** | | **Imprecision** | | **Publication bias** | |  |  |  |  |
| **Overall toxicity** | | | | | | | | | | | | | | | | | |
| cTNT vs iTNT | 2 | | randomised trials, observational studies | | not serious | | not serious | | not serious | | serious^f^ | | none | cTNT:  212 | iTNT:  191 | ⨁⨁⨁◯ Moderate | |
| cTNT vs nCRT | 4 | | randomised trials | | not serious | | not serious | | not serious | | serious^f^ | | none | cTNT:  894 | nCRT: 858 | ⨁⨁⨁◯ Moderate | |
| iTNT vs nCRT | 5 | | randomised trials, observational studies | | not serious | | not serious | | not serious | | serious^f^ | | none | iTNT: 1236 | nCRT: 1095 | ⨁⨁⨁◯ Moderate | |
| **Rectal bleeding** | | | | | | | | | | | | | | | | | |
| cTNT vs iTNT | 2 | | randomised trials, observational studies | | not serious | | not serious | | not serious | | not serious | | none | cTNT:  212 | iTNT:  191 | ⨁⨁⨁⨁ High | |
| cTNT vs nCRT | 5 | | randomised trials | | not serious | | not serious | | not serious | | serious^f^ | | none | cTNT:  1038 | nCRT: 1069 | ⨁⨁⨁◯ Moderate | |
| iTNT vs nCRT | 5 | | randomised trials | | not serious | | not serious | | not serious | | serious^f^ | | none | iTNT: 1148 | nCRT: 1185 | ⨁⨁⨁◯ Moderate | |
| **Proctitis** | | | | | | | | | | | | | | | | | |
| cTNT vs iTNT | 1 | | randomised trials | | not serious | | not serious | | not serious | | not serious | | none | cTNT:  166 | iTNT:  158 | ⨁⨁⨁⨁ High | |
| cTNT vs nCRT | 5 | | randomised trials | | serious^c^ | | not serious | | not serious | | serious^f^ | | none | cTNT:  946 | nCRT: 892 | ⨁⨁⨁◯ Moderate | |
| iTNT vs nCRT | 5 | | randomised trials | | not serious | | not serious | | not serious | | serious^f^ | | none | iTNT: 1008 | nCRT: 988 | ⨁⨁⨁◯ Moderate | |
| **Mucositis** | | | | | | | | | | | | | | | | | |
| cTNT vs iTNT | 1 | | randomised trials | | not serious | | serious^a^ | | not serious | | serious^f^ | | none | cTNT:  166 | iTNT:  158 | ⨁⨁◯◯ Low | |
| cTNT vs nCRT | 1 | | randomised trials | | not serious | | serious^a^ | | not serious | | not serious | | none | cTNT:  460 | nCRT: 441 | ⨁⨁⨁◯ Moderate | |
| iTNT vs nCRT | 3 | | randomised trials | | not serious | | serious^a^ | | not serious | | not serious | | none | iTNT: 782 | nCRT: 759 | ⨁⨁⨁◯ Moderate | |
| **Oesophagitis** | | | | | | | | | | | | | | | | | |
| cTNT vs iTNT | 1 | | randomised trials | | not serious | | not serious | | not serious | | serious^f^ | | none | cTNT:  166 | iTNT:  158 | ⨁⨁⨁◯ Moderate | |
| cTNT vs nCRT | 1 | | randomised trials | | not serious | | not serious | | not serious | | not serious | | none | cTNT:  460 | nCRT: 441 | ⨁⨁⨁⨁ High | |
| iTNT vs nCRT | 1 | | randomised trials | | not serious | | not serious | | not serious | | not serious | | none | iTNT: 225 | nCRT: 158 | ⨁⨁⨁⨁ High | |
| **Enterocolitis** | | | | | | | | | | | | | | | | | |
| cTNT vs iTNT | 1 | | randomised trials, observational studies | | not serious | | not serious | | not serious | | serious^f^ | | none | cTNT:  166 | iTNT:  158 | ⨁⨁⨁◯ Moderate | |
| cTNT vs nCRT | 1 | | randomised trials | | not serious | | not serious | | not serious | | not serious | | none | cTNT:  460 | nCRT: 441 | ⨁⨁⨁⨁ High | |
| iTNT vs nCRT | 1 | | randomised trials | | not serious | | not serious | | not serious | | not serious | | none | iTNT: 225 | nCRT: 158 | ⨁⨁⨁⨁ High | |
| **Diarrhoea** | | | | | | | | | | | | | | | | | |
| cTNT vs iTNT | 2 | | randomised trials, observational studies | | not serious | | not serious | | not serious | | serious^f^ | | none | cTNT:  212 | iTNT:  191 | ⨁⨁⨁◯ Moderate | |
| cTNT vs nCRT | 6 | | randomised trials, observational studies | | not serious | | not serious | | not serious | | serious^f^ | | none | cTNT:  923 | nCRT: 1039 | ⨁⨁⨁◯ Moderate | |
| iTNT vs nCRT | 5 | | randomised trials | | not serious | | not serious | | not serious | | serious^f^ | | none | iTNT: 948 | nCRT: 1058 | ⨁⨁⨁◯ Moderate | |
| **Vomiting** | | | | | | | | | | | | | | | | | |
| cTNT vs iTNT | 2 | | randomised trials, observational studies | | not serious | | not serious | | not serious | | not serious | | none | cTNT:  212 | iTNT:  191 | ⨁⨁⨁⨁ High | |
| cTNT vs nCRT | 4 | | randomised trials, observational studies | | not serious | | not serious | | not serious | | not serious | | none | cTNT:  792 | nCRT: 743 | ⨁⨁⨁⨁ High | |
| iTNT vs nCRT | 3 | | randomised trials | | not serious | | not serious | | not serious | | not serious | | none | iTNT: 435 | nCRT: 431 | ⨁⨁⨁⨁ High | |
| **Bowel obstruction** | | | | | | | | | | | | | | | | | |
| cTNT vs iTNT | 2 | | randomised trials, observational studies | | not serious | | not serious | | not serious | | serious^f^ | | none | cTNT:  212 | iTNT:  191 | ⨁⨁⨁◯ Moderate | |
| cTNT vs nCRT | 1 | | randomised trials | | not serious | | not serious | | not serious | | serious^f^ | | none | cTNT:  460 | nCRT: 441 | ⨁⨁⨁◯ Moderate | |
| iTNT vs nCRT | 1 | | randomised trials | | not serious | | not serious | | not serious | | serious^f^ | | none | iTNT: 585 | nCRT: 543 | ⨁⨁⨁◯ Moderate | |
| **Pancreatitis** | | | | | | | | | | | | | | | | | |
| cTNT vs iTNT | 2 | | randomised trials, observational studies | | not serious | | not serious | | not serious | | serious^f^ | | none | cTNT:  212 | iTNT:  191 | ⨁⨁⨁◯ Moderate | |
| cTNT vs nCRT | 5 | | randomised trials | | not serious | | not serious | | not serious | | serious^f^ | | none | cTNT:  946 | nCRT: 892 | ⨁⨁⨁◯ Moderate | |
| **Neuropathy** | | | | | | | | | | | | | | | | | |
| cTNT vs iTNT | 2 | | randomised trials, observational studies | | not serious | | not serious | | not serious | | serious^f^ | | none | cTNT:  212 | iTNT:  191 | ⨁⨁⨁◯ Moderate | |
| cTNT vs nCRT | 5 | | randomised trials | | serious^c^ | | not serious | | not serious | | not serious | | none | cTNT:  1104 | nCRT: 1095 | ⨁⨁⨁◯ Moderate | |
| iTNT vs nCRT | 4 | | randomised trials, observational studies | | not serious | | not serious | | not serious | | not serious | | none | iTNT: 848 | nCRT: 875 | ⨁⨁⨁⨁ High | |
| **Dizziness** | | | | | | | | | | | | | | | | | |
| cTNT vs iTNT | 2 | | randomised trials, observational studies | | not serious | | not serious | | not serious | | serious^f^ | | none | cTNT:  212 | iTNT:  191 | ⨁⨁⨁◯ Moderate | |
| cTNT vs nCRT | 1 | | randomised trials | | not serious | | not serious | | not serious | | serious^f^ | | none | cTNT:  460 | nCRT: 441 | ⨁⨁⨁◯ Moderate | |
| iTNT vs nCRT | 1 | | randomised trials | | not serious | | not serious | | not serious | | serious^f^ | | none | iTNT: 585 | nCRT: 543 | ⨁⨁⨁◯ Moderate | |
| **Dysarthria** | | | | | | | | | | | | | | | | | |
| cTNT vs iTNT | 2 | | randomised trials, observational studies | | not serious | | not serious | | not serious | | serious^f^ | | none | cTNT:  212 | iTNT:  191 | ⨁⨁⨁◯ Moderate | |
| cTNT vs nCRT | 2 | | randomised trials | | not serious | | not serious | | not serious | | serious^f^ | | none | cTNT:  329 | nCRT: 322 | ⨁⨁⨁◯ Moderate | |
| iTNT vs nCRT | 4 | | randomised trials | | not serious | | not serious | | not serious | | serious^f^ | | none | iTNT: 938 | nCRT: 949 | ⨁⨁⨁◯ Moderate | |
| **Hand foot syndrome** | | | | | | | | | | | | | | | | | |
| cTNT vs iTNT | 2 | | randomised trials, observational studies | | not serious | | not serious | | not serious | | serious^f^ | | none | cTNT:  212 | iTNT:  191 | ⨁⨁⨁◯ Moderate | |
| cTNT vs nCRT | 2 | | randomised trials | | not serious | | not serious | | not serious | | serious^f^ | | none | cTNT:  329 | nCRT: 322 | ⨁⨁⨁◯ Moderate | |
| iTNT vs nCRT | 4 | | randomised trials | | not serious | | not serious | | not serious | | serious^f^ | | none | iTNT: 938 | nCRT: 949 | ⨁⨁⨁◯ Moderate | |
| **Syncope** | | | | | | | | | | | | | | | | | |
| cTNT vs iTNT | 2 | | randomised trials, observational studies | | not serious | | not serious | | not serious | | serious^f^ | | none | cTNT:  212 | iTNT:  191 | ⨁⨁⨁◯ Moderate | |
| cTNT vs nCRT | 2 | | randomised trials | | not serious | | not serious | | not serious | | serious^f^ | | none | cTNT:  163 | nCRT: 175 | ⨁⨁⨁◯ Moderate | |
| iTNT vs nCRT | 2 | | randomised trials, observational studies | | not serious | | not serious | | not serious | | serious^f^ | | none | iTNT: 524 | nCRT: 543 | ⨁⨁⨁◯ Moderate | |
| **Musculoskeletal weakness** | | | | | | | | | | | | | | | | | |
| cTNT vs iTNT | 1 | | randomised trials | | not serious | | not serious | | not serious | | serious^f^ | | none | cTNT:  166 | iTNT:  158 | ⨁⨁⨁◯ Moderate | |
| cTNT vs nCRT | 2 | | randomised trials | | not serious | | not serious | | not serious | | serious^f^ | | none | cTNT:  163 | nCRT: 175 | ⨁⨁⨁◯ Moderate | |
| iTNT vs nCRT | 3 | | randomised trials | | not serious | | not serious | | not serious | | serious^f^ | | none | iTNT: 596 | nCRT: 620 | ⨁⨁⨁◯ Moderate | |
| **Venous thromboembolism** | | | | | | | | | | | | | | | | | |
| cTNT vs iTNT | 2 | | randomised trials, observational studies | | not serious | | not serious | | not serious | | serious^f^ | | none | cTNT:  212 | iTNT:  191 | ⨁⨁⨁◯ Moderate | |
| cTNT vs nCRT | 4 | | randomised trials | | not serious | | not serious | | not serious | | not serious | | none | cTNT:  792 | nCRT: 743 | ⨁⨁⨁⨁ High | |
| iTNT vs nCRT | 2 | | randomised trials | | serious^c^ | | not serious | | not serious | | not serious | | none | iTNT: 402 | nCRT: 392 | ⨁⨁⨁◯ Moderate | |
| **Arrythmias** | | | | | | | | | | | | | | | | | |
| cTNT vs iTNT | 2 | | randomised trials, observational studies | | not serious | | not serious | | not serious | | serious^f^ | | none | cTNT:  212 | iTNT:  191 | ⨁⨁⨁◯ Moderate | |
| cTNT vs nCRT | 4 | | randomised trials | | not serious | | not serious | | not serious | | serious^f^ | | none | cTNT:  792 | nCRT: 743 | ⨁⨁⨁◯ Moderate | |
| iTNT vs nCRT | 4 | | randomised trials, observational studies | | serious^c^ | | not serious | | not serious | | serious^f^ | | none | iTNT: 639 | nCRT: 620 | ⨁⨁⨁◯ Moderate | |
| **Pneumonia** | | | | | | | | | | | | | | | | | |
| cTNT vs iTNT | 2 | | randomised trials, observational studies | | not serious | | serious^a^ | | not serious | | serious^f^ | | none | cTNT:  212 | iTNT:  191 | ⨁⨁◯◯ Low | |
| cTNT vs nCRT | 2 | | randomised trials | | not serious | | serious^a^ | | not serious | | serious^f^ | | none | cTNT:  163 | nCRT: 175 | ⨁⨁◯◯ Low | |
| iTNT vs nCRT | 4 | | randomised trials, observational studies | | serious^c^ | | serious^a^ | | not serious | | serious^f^ | | none | iTNT: 639 | nCRT: 620 | ⨁◯◯◯ Very low | |
| **Urinary tract infection** | | | | | | | | | | | | | | | | | |
| cTNT vs iTNT | 2 | | randomised trials, observational studies | | not serious | | not serious | | not serious | | serious^f^ | | none | cTNT:  212 | iTNT:  191 | ⨁⨁⨁◯ Moderate | |
| cTNT vs nCRT | 3 | | randomised trials | | not serious | | not serious | | not serious | | serious^f^ | | none | cTNT:  632 | nCRT: 743 | ⨁⨁⨁◯ Moderate | |
| iTNT vs nCRT | 3 | | randomised trials | | not serious | | not serious | | not serious | | serious^f^ | | none | iTNT: 233 | nCRT: 198 | ⨁⨁⨁◯ Moderate | |
| **Sepsis** | | | | | | | | | | | | | | | | | |
| cTNT vs iTNT | 2 | | randomised trials, observational studies | | not serious | | not serious | | not serious | | serious^f^ | | none | cTNT:  212 | iTNT:  191 | ⨁⨁⨁◯ Moderate | |
| cTNT vs nCRT | 1 | | randomised trials | | not serious | | not serious | | not serious | | serious^f^ | | none | cTNT:  120 | nCRT: 143 | ⨁⨁⨁◯ Moderate | |
| iTNT vs nCRT | 2 | | randomised trials, observational studies | | not serious | | not serious | | not serious | | serious^f^ | | none | iTNT: 142 | nCRT: 184 | ⨁⨁⨁◯ Moderate | |
| **Radiation dermatitis** | | | | | | | | | | | | | | | | | |
| cTNT vs iTNT | 2 | | randomised trials, observational studies | | not serious | | not serious | | not serious | | serious^f^ | | none | cTNT:  212 | iTNT:  191 | ⨁⨁⨁◯ Moderate | |
| cTNT vs nCRT | 4 | | randomised trials | | not serious | | not serious | | not serious | | serious^f^ | | none | cTNT:  792 | nCRT: 743 | ⨁⨁⨁◯ Moderate | |
| iTNT vs nCRT | 1 | | randomised trials | | not serious | | not serious | | not serious | | serious^f^ | | none | iTNT: 158 | nCRT: 155 | ⨁⨁⨁◯ Moderate | |
| **Renal and electrolyte disturbances** | | | | | | | | | | | | | | | | | |
| cTNT vs iTNT | 2 | | randomised trials, observational studies | | not serious | | not serious | | not serious | | serious^f^ | | none | cTNT:  212 | iTNT:  191 | ⨁⨁⨁◯ Moderate | |
| cTNT vs nCRT | 6 | | randomised trials, observational studies | | not serious | | not serious | | not serious | | serious^f^ | | none | cTNT:  1420 | nCRT: 1345 | ⨁⨁⨁◯ Moderate | |
| iTNT vs nCRT | 2 | | randomised trials | | not serious | | not serious | | not serious | | serious^f^ | | none | iTNT: 212 | nCRT: 252 | ⨁⨁⨁◯ Moderate | |
| **Febrile neutropaenia** | | | | | | | | | | | | | | | | | |
| cTNT vs iTNT | 2 | | randomised trials, observational studies | | not serious | | not serious | | not serious | | serious^f^ | | none | cTNT:  212 | iTNT:  191 | ⨁⨁⨁◯ Moderate | |
| cTNT vs nCRT | 3 | | randomised trials | | not serious | | not serious | | not serious | | not serious | | none | cTNT:  420 | nCRT: 445 | ⨁⨁⨁⨁ High | |
| iTNT vs nCRT | 3 | | randomised trials | | not serious | | not serious | | not serious | | not serious | | none | iTNT: 523 | nCRT: 612 | ⨁⨁⨁⨁ High | |
| **Neutropenia** | | | | | | | | | | | | | | | | | |
| cTNT vs iTNT | 2 | | randomised trials, observational studies | | not serious | | not serious | | not serious | | serious^f^ | | none | cTNT:  212 | iTNT:  191 | ⨁⨁⨁◯ Moderate | |
| cTNT vs nCRT | 5 | | randomised trials | | not serious | | not serious | | not serious | | not serious | | none | cTNT:  1010 | nCRT: 1153 | ⨁⨁⨁⨁ High | |
| iTNT vs nCRT | 5 | | randomised trials, observational studies | | not serious | | not serious | | not serious | | not serious | | none | iTNT: 1283 | nCRT: 1325 | ⨁⨁⨁⨁ High | |
| **Lymphopenia** | | | | | | | | | | | | | | | | | |
| cTNT vs iTNT | 2 | | randomised trials, observational studies | | not serious | | not serious | | not serious | | not serious | | none | cTNT:  212 | iTNT:  191 | ⨁⨁⨁⨁ High | |
| cTNT vs nCRT | 4 | | randomised trials | | not serious | | not serious | | not serious | | serious^f^ | | none | cTNT:  927 | nCRT: 969 | ⨁⨁⨁◯ Moderate | |
| iTNT vs nCRT | 4 | | randomised trials | | not serious | | not serious | | not serious | | serious^f^ | | none | iTNT: 1029 | nCRT: 1392 | ⨁⨁⨁◯ Moderate | |
| **Thrombocytopenia** | | | | | | | | | | | | | | | | | |
| cTNT vs iTNT | 2 | | randomised trials, observational studies | | not serious | | not serious | | not serious | | serious^f^ | | none | cTNT:  212 | iTNT:  191 | ⨁⨁⨁◯ Moderate | |
| cTNT vs nCRT | 6 | | randomised trials, observational studies | | not serious | | not serious | | not serious | | serious^f^ | | none | cTNT:  1341 | nCRT: 1376 | ⨁⨁⨁◯ Moderate | |
| iTNT vs nCRT | 4 | | randomised trials | | serious^c^ | | not serious | | not serious | | serious^f^ | | none | iTNT: 1283 | nCRT: 1325 | ⨁⨁◯◯ Low | |
| **Anaemia** | | | | | | | | | | | | | | | | | |
| cTNT vs iTNT | 2 | | randomised trials, observational studies | | not serious | | not serious | | not serious | | serious^f^ | | none | cTNT:  212 | iTNT:  191 | ⨁⨁⨁◯ Moderate | |
| cTNT vs nCRT | 4 | | randomised trials | | not serious | | not serious | | not serious | | serious^f^ | | none | cTNT:  1121 | nCRT: 1231 | ⨁⨁⨁◯ Moderate | |
| iTNT vs nCRT | 4 | | randomised trials | | not serious | | not serious | | not serious | | serious^f^ | | none | iTNT: 1283 | nCRT: 1325 | ⨁⨁⨁◯ Moderate | |
| **Anaphylaxis** | | | | | | | | | | | | | | | | | |
| cTNT vs iTNT | 2 | | randomised trials, observational studies | | not serious | | not serious | | not serious | | serious^f^ | | none | cTNT:  212 | iTNT:  191 | ⨁⨁⨁◯ Moderate | |
| cTNT vs nCRT | 2 | | randomised trials | | not serious | | not serious | | not serious | | serious^f^ | | none | cTNT:  163 | nCRT: 175 | ⨁⨁⨁◯ Moderate | |
| iTNT vs nCRT | 4 | | randomised trials | | not serious | | not serious | | not serious | | serious^f^ | | none | iTNT: 984 | nCRT: 1158 | ⨁⨁⨁◯ Moderate | |

#### Explanations

a. Inconsistency P-value <0.05

b. Underpowered sample size

c. ≥50% at high risk of bias

d. Cross one threshold of clinically significant effect size

f. Cross one threshold of statistically significant effect size

e. ≥50% at unclear risk of bias

*Compliance outcomes*

|  | **Certainty assessment** | | | | | | | | | | | | | **№ of patients** | | **Certainty** |  |
| --- | --- | --- | --- | --- | --- | --- | --- | --- | --- | --- | --- | --- | --- | --- | --- | --- | --- |
| **Direct comparisons** | **№ of studies** | **Study design** | | **Risk of bias** | | **Inconsistency** | | **Indirectness** | | **Imprecision** | | **Publication bias** | |  |  |  |  |
| **Compliance with radiotherapy** | | | | | | | | | | | | | | | | | |
| cTNT vs iTNT | 2 | | randomised trials, observational studies | | not serious | | not serious | | not serious | | not serious | | none | cTNT:  212 | iTNT:  191 | ⨁⨁⨁⨁ High | |
| cTNT vs nCRT | 2 | | randomised trials | | not serious | | not serious | | not serious | | not serious | | none | cTNT:  447 | nCRT: 446 | ⨁⨁⨁⨁ High | |
| iTNT vs nCRT | 5 | | randomised trials, observational studies | | not serious | | not serious | | not serious | | not serious | | none | iTNT: 1131 | nCRT: 976 | ⨁⨁⨁⨁ High | |
| **Compliance with chemotherapy** | | | | | | | | | | | | | | | | | |
| cTNT vs iTNT | 3 | | randomised trials, observational studies | | not serious | | not serious | | not serious | | serious^f^ | | none | cTNT:  296 | iTNT:  274 | ⨁⨁⨁◯ Moderate | |
| cTNT vs nCRT | 7 | | randomised trials, observational studies | | not serious | | not serious | | not serious | | serious^f^ | | none | cTNT:  1524 | nCRT: 1643 | ⨁⨁⨁◯ Moderate | |
| iTNT vs nCRT | 7 | | randomised trials, observational studies | | not serious | | not serious | | not serious | | serious^f^ | | none | iTNT: 1743 | nCRT: 1872 | ⨁⨁⨁◯ Moderate | |
| **Compliance with FOLFOX-specific chemotherapy** | | | | | | | | | | | | | | | | | |
| cTNT vs iTNT | 2 | | randomised trials, observational studies | | not serious | | not serious | | not serious | | serious^f^ | | none | cTNT:  212 | iTNT:  191 | ⨁⨁⨁◯ Moderate | |
| cTNT vs nCRT | 5 | | randomised trials | | serious^c^ | | not serious | | not serious | | serious^f^ | | none | cTNT:  859 | nCRT: 912 | ⨁⨁⨁◯ Moderate | |
| iTNT vs nCRT | 5 | | randomised trials, observational studies | | not serious | | not serious | | not serious | | serious^f^ | | none | iTNT: 754 | nCRT: 812 | ⨁⨁⨁◯ Moderate | |
| **Compliance with CAPOX-specific chemotherapy** | | | | | | | | | | | | | | | | | |
| cTNT vs iTNT | 2 | | randomised trials, observational studies | | not serious | | not serious | | not serious | | serious^f^ | | none | cTNT:  212 | iTNT:  191 | ⨁⨁⨁◯ Moderate | |
| cTNT vs nCRT | 4 | | randomised trials | | not serious | | not serious | | not serious | | serious^f^ | | none | cTNT:  812 | nCRT: 856 | ⨁⨁⨁◯ Moderate | |
| iTNT vs nCRT | 2 | | randomised trials | | not serious | | not serious | | not serious | | serious^f^ | | none | iTNT: 212 | nCRT: 252 | ⨁⨁⨁◯ Moderate | |

#### Explanations

a. Inconsistency P-value <0.05

b. Underpowered sample size

c. ≥50% at high risk of bias

d. Cross one threshold of clinically significant effect size

f. Cross one threshold of statistically significant effect size

e. ≥50% at unclear risk of bias

*Postoperative outcomes*

|  | **Certainty assessment** | | | | | | | | | | | | | **№ of patients** | | **Certainty** |  |
| --- | --- | --- | --- | --- | --- | --- | --- | --- | --- | --- | --- | --- | --- | --- | --- | --- | --- |
| **Direct comparisons** | **№ of studies** | **Study design** | | **Risk of bias** | | **Inconsistency** | | **Indirectness** | | **Imprecision** | | **Publication bias** | |  |  |  |  |
| **Overall Clavien-Dindo (CD) grade 3 and above postoperative complications** | | | | | | | | | | | | | | | | | |
| cTNT vs iTNT | 1 | | randomised trials | | not serious | | not serious | | not serious | | serious^f^ | | none | cTNT:  166 | iTNT:  158 | ⨁⨁⨁◯ Moderate | |
| cTNT vs nCRT | 5 | | randomised trials, observational studies | | not serious | | not serious | | not serious | | serious^f^ | | none | cTNT:  859 | nCRT: 912 | ⨁⨁⨁◯ Moderate | |
| iTNT vs nCRT | 3 | | randomised trials | | not serious | | not serious | | not serious | | serious^f^ | | none | iTNT: 523 | nCRT: 612 | ⨁⨁⨁◯ Moderate | |
| **Diarrhoea/high stoma output** | | | | | | | | | | | | | | | | | |
| cTNT vs iTNT | 1 | | randomised trials | | not serious | | not serious | | not serious | | not serious | | none | cTNT:  166 | iTNT:  158 | ⨁⨁⨁⨁ High | |
| cTNT vs nCRT | 4 | | randomised trials | | not serious | | not serious | | not serious | | serious^f^ | | none | cTNT:  1121 | nCRT: 1231 | ⨁⨁⨁◯ Moderate | |
| iTNT vs nCRT | 4 | | randomised trials | | not serious | | not serious | | not serious | | not serious | | none | iTNT: 1283 | nCRT: 1325 | ⨁⨁⨁⨁ High | |
| **Anastomotic leak** | | | | | | | | | | | | | | | | | |
| cTNT vs iTNT | 1 | | randomised trials | | not serious | | not serious | | not serious | | serious^f^ | | none | cTNT:  166 | iTNT:  158 | ⨁⨁⨁◯ Moderate | |
| cTNT vs nCRT | 5 | | randomised trials, observational studies | | serious^c^ | | not serious | | not serious | | serious^f^ | | none | cTNT:  859 | nCRT: 912 | ⨁⨁◯◯ Low | |
| iTNT vs nCRT | 2 | | randomised trials | | not serious | | not serious | | not serious | | serious^f^ | | none | iTNT: 212 | nCRT: 252 | ⨁⨁⨁◯ Moderate | |
| **Postoperative bowel obstruction** | | | | | | | | | | | | | | | | | |
| cTNT vs iTNT | 1 | | randomised trials | | not serious | | not serious | | not serious | | serious^f^ | | none | cTNT:  166 | iTNT:  158 | ⨁⨁⨁◯ Moderate | |
| cTNT vs nCRT | 2 | | randomised trials | | not serious | | not serious | | not serious | | serious^f^ | | none | cTNT:  241 | nCRT: 312 | ⨁⨁⨁◯ Moderate | |
| iTNT vs nCRT | 4 | | randomised trials | | not serious | | not serious | | not serious | | serious^f^ | | none | iTNT: 639 | nCRT: 620 | ⨁⨁⨁◯ Moderate | |
| **Organ/space surgical site infection (SSI)** | | | | | | | | | | | | | | | | | |
| cTNT vs iTNT | 2 | | randomised trials, observational studies | | not serious | | not serious | | not serious | | serious^f^ | | none | cTNT:  212 | iTNT:  191 | ⨁⨁⨁◯ Moderate | |
| cTNT vs nCRT | 5 | | randomised trials | | not serious | | not serious | | not serious | | serious^f^ | | none | cTNT:  974 | nCRT: 891 | ⨁⨁⨁◯ Moderate | |
| iTNT vs nCRT | 2 | | randomised trials | | not serious | | not serious | | not serious | | serious^f^ | | none | iTNT: 225 | nCRT: 158 | ⨁⨁⨁◯ Moderate | |
| **Superficial surgical site infection (SSI)** | | | | | | | | | | | | | | | | | |
| cTNT vs iTNT | 1 | | randomised trials | | not serious | | not serious | | not serious | | serious^f^ | | none | cTNT:  166 | iTNT:  158 | ⨁⨁⨁◯ Moderate | |
| cTNT vs nCRT | 3 | | randomised trials | | not serious | | not serious | | not serious | | serious^f^ | | none | cTNT:  632 | nCRT: 641 | ⨁⨁⨁◯ Moderate | |
| iTNT vs nCRT | 3 | | randomised trials | | not serious | | not serious | | not serious | | serious^f^ | | none | iTNT: 745 | nCRT: 756 | ⨁⨁⨁◯ Moderate | |
| **Postoperative UTI** | | | | | | | | | | | | | | | | | |
| cTNT vs iTNT | 1 | | randomised trials | | not serious | | not serious | | not serious | | serious^f^ | | none | cTNT:  166 | iTNT:  158 | ⨁⨁⨁◯ Moderate | |
| cTNT vs nCRT | 4 | | randomised trials, observational studies | | not serious | | not serious | | not serious | | serious^f^ | | none | cTNT:  823 | nCRT: 865 | ⨁⨁⨁◯ Moderate | |
| iTNT vs nCRT | 3 | | randomised trials | | not serious | | not serious | | not serious | | serious^f^ | | none | iTNT: 745 | nCRT: 756 | ⨁⨁⨁◯ Moderate | |

#### Explanations

a. Inconsistency P-value <0.05

b. Underpowered sample size

c. ≥50% at high risk of bias

d. Cross one threshold of clinically significant effect size

f. Cross one threshold of statistically significant effect size

e. ≥50% at unclear risk of bias

## Appendix 13: Additional details of GRADE assessment to assess certainty of findings

The GRADE approach provides a rating for the quality of the estimates of effect for a specific comparison and outcome. In brief, the ratings are as follows:

| ⨁⨁⨁◯ High | We are very confident that the true effect lies close to that of the estimate of the effect. |
| --- | --- |
| ⨁⨁⨁◯ Moderate | We are moderately confident in the effect estimate: the true effect is likely to be close to the estimate of the effect,  but there is a possibility that it is substantially different. |
| ⨁⨁◯◯ Low | Our confidence in the effect estimate is limited: the true effect may be substantially different from the estimate of the effect. |
| ⨁◯◯◯ Very low | We have very little confidence in the effect estimate: the true effect is likely to be substantially different from the estimate of effect. |

The starting point of each rating starts high for randomised controlled trials. Given the ROBINS-I tool was used to assess risk of bias for NRSI, the starting point of each rating starts high for these non-randomised studies(1). Down rating may be rated down by -1 (serious concern) or -2 (very serious concern) for the following reasons:

**Risk of bias**

Risk of bias was assessed by 2 reviewers (W.S, Z.B) using the Risk of Bias Tool 2.0. When there were disagreements, a third author was consulted (S.B). For GRADE analysis, if >50% of the studies included in a direct comparison between interventions were at an unclear risk of, the comparison was downgraded one stage. If >50% of the studies within a direct comparison were at high risk of bias, the comparison evidence quality was downgraded two stages.

**Indirectness**

Indirectness is the assumption at the population groups are similar in demographics so that the assumption of transitivity can be maintained. Evidence was downgraded if the study question of the included RCT was not relevant the question of the review.

**Inconsistency**

To assess for inconsistency, we used the node splitting approach. The node-splitting approach separates the direct evidence and the indirect estimates of a treatment loop. If the direct and indirect comparisons were not different, as measured by the inconsistency P-value, evidence was not downgraded. If the inconsistency P-values were >0.05, the evidence was downgraded.

**Imprecision**

Assessment of imprecision was guided by the GRADE handbook. The 95% CrI of an estimate was inspected for imprecision. In the case the intervals crossed the no effect line only, or the clinically important effect sizes only, the estimate was not downgraded. When intervals crossed the no effect line and the threshold for a clinically important effect size, the estimate was downgraded 1 additional stage. When the intervals crossed both thresholds of clinically important effect size, the estimate was downgraded 2 additional stages.

**Publication bias**

Assessment of publication bias was guided by the GRADE handbook. Comparison-adjusted funnel plots were generated for all outcomes in our study. In brief, the criterion for publication bias is the pattern of study results. Evidence of asymmetry in funnel plots for each outcomes were visually inspected for suspicion of publication bias. The evidence was downgraded if publication bias was identified.

## Appendix 14: Funnel plot of all outcomes

| **Toxicity outcomes** | |
| --- | --- |
| 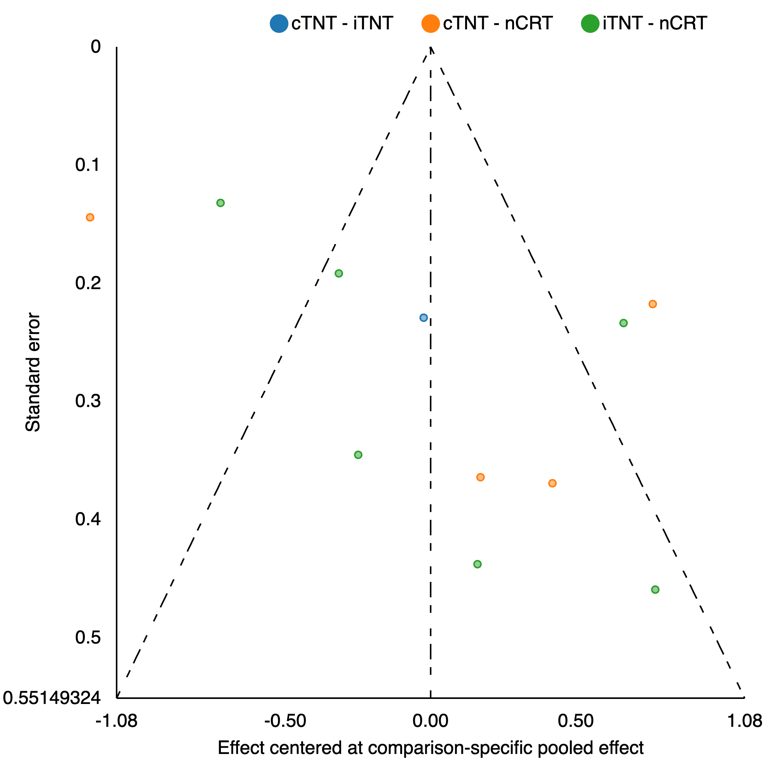 | 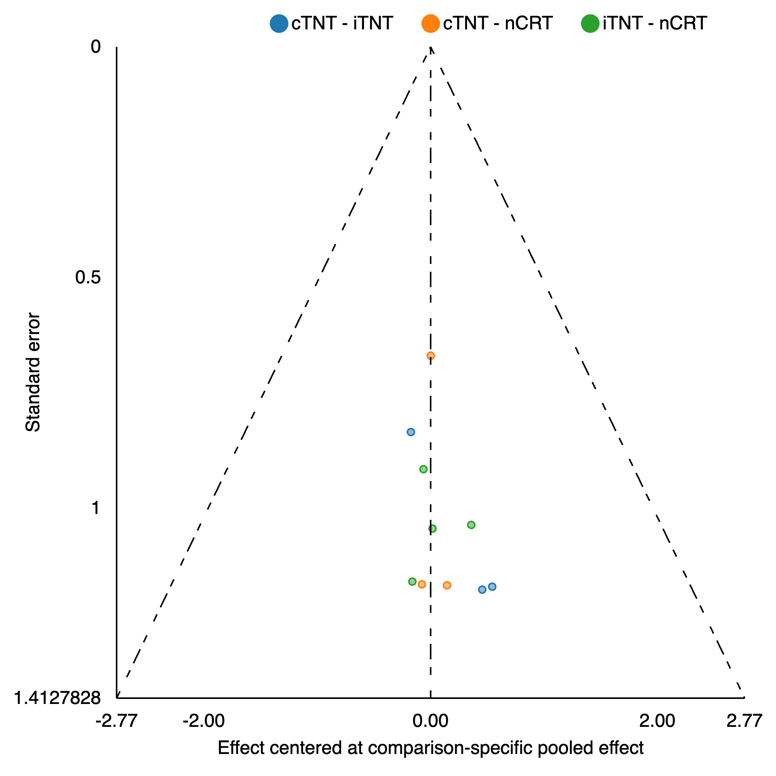 |
| Overall Grade III and above treatment-related adverse events | Rate of treatment-related mortality |
| *Individual rate of treatment-related adverse events* | |
| 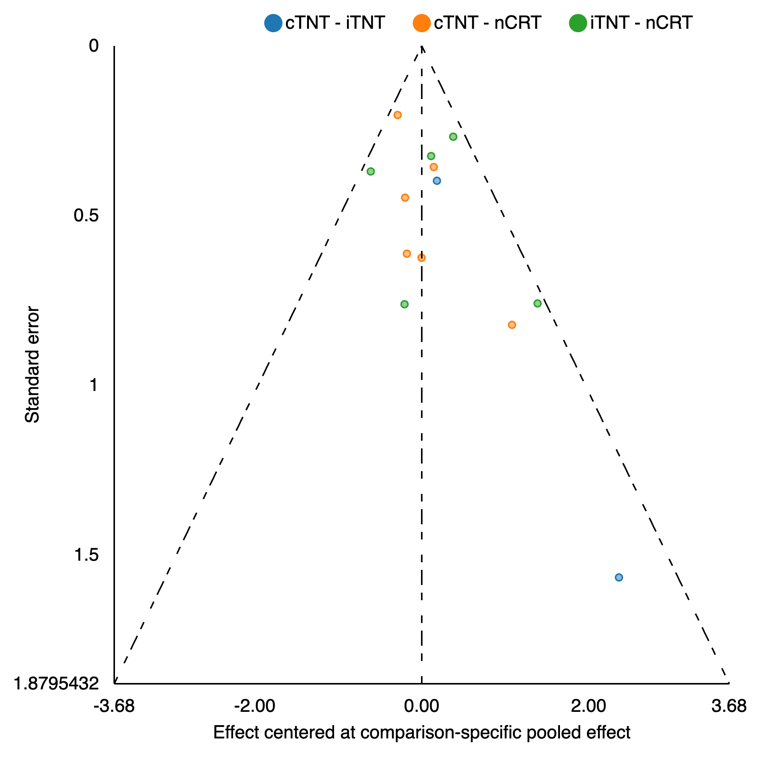 | 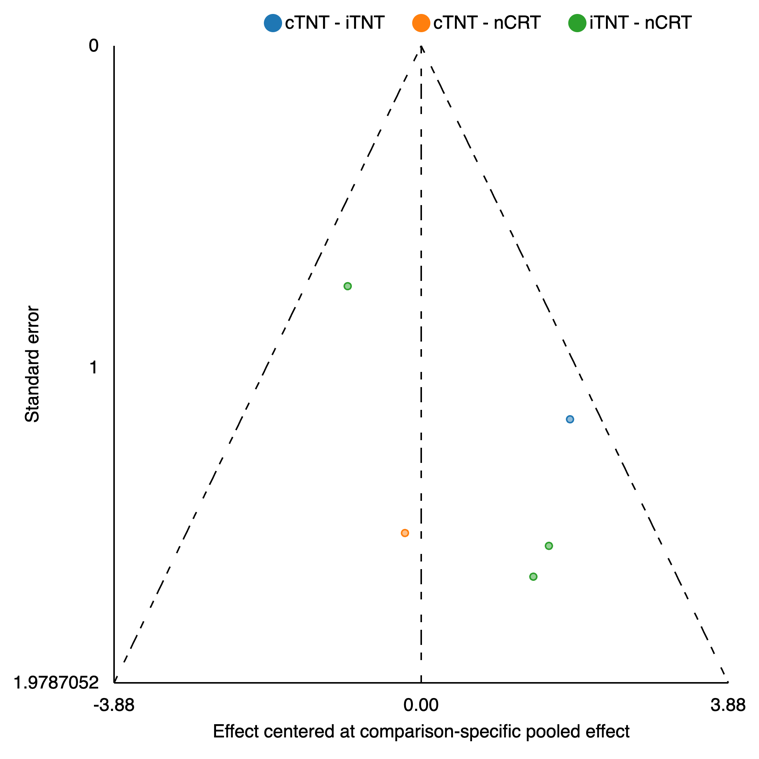 |
| Diarrhoea | Mucositis |
| 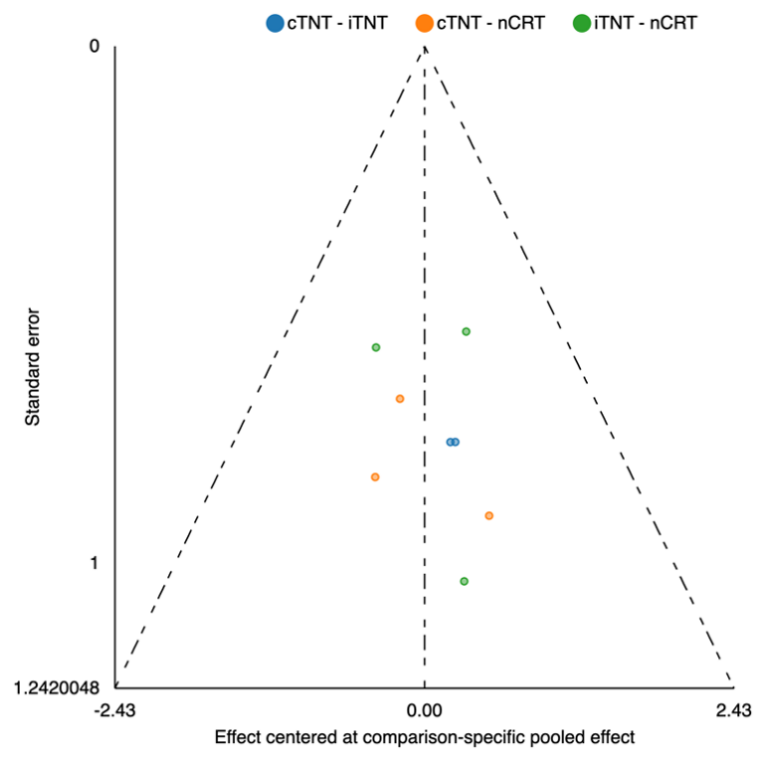 | 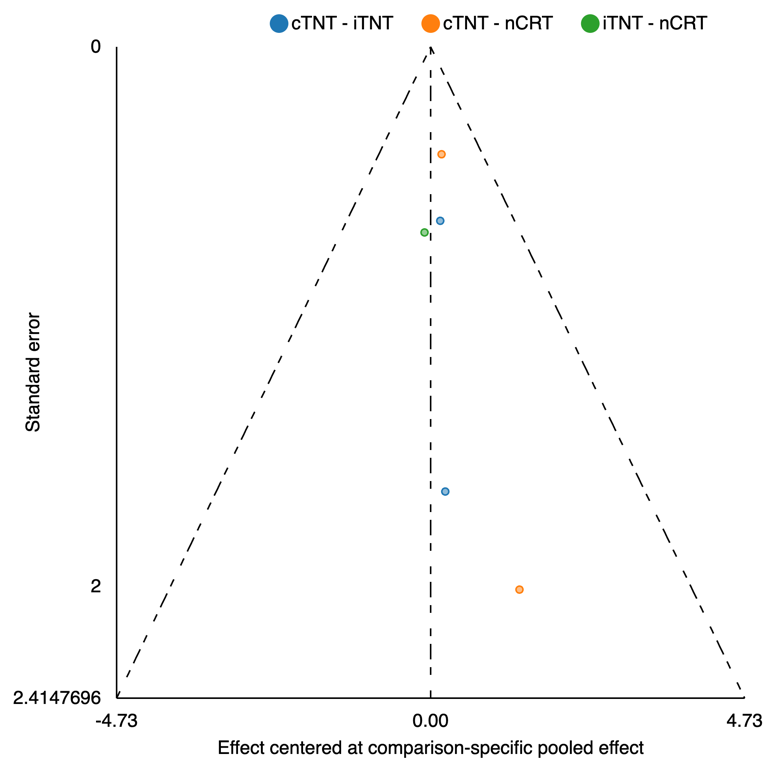 |
| Oesophagitis | Enterocolitis |
| 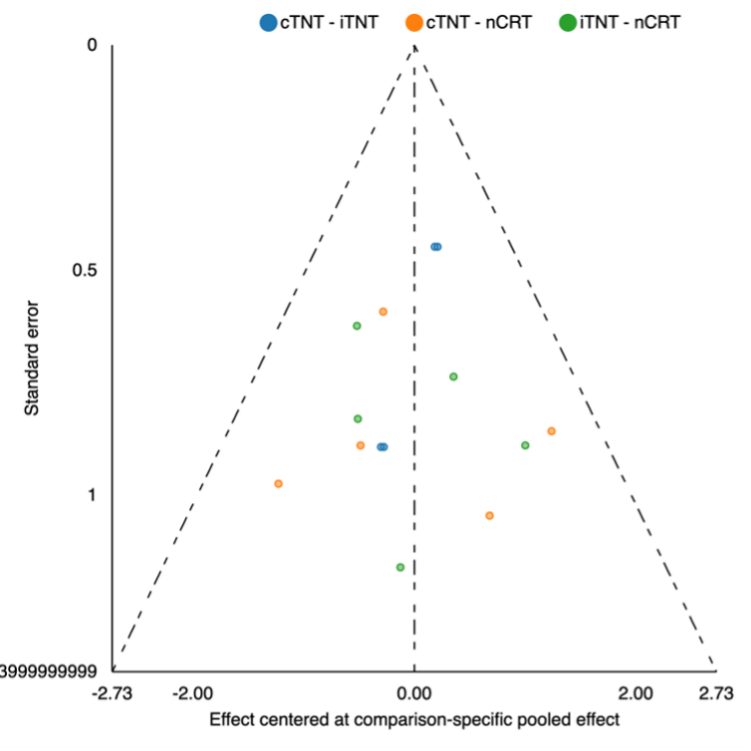 | 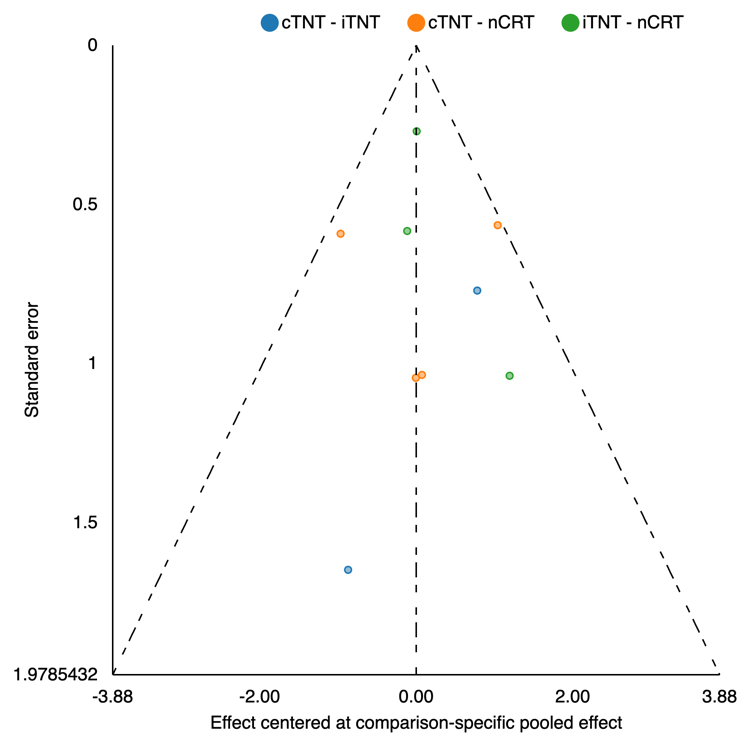 |
| Vomiting | Nausea without vomiting |
| 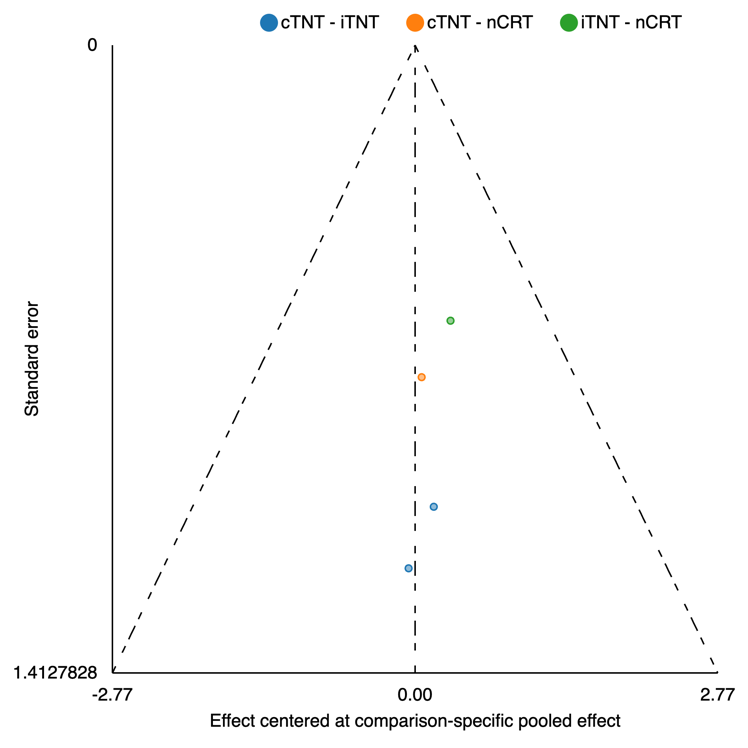 | 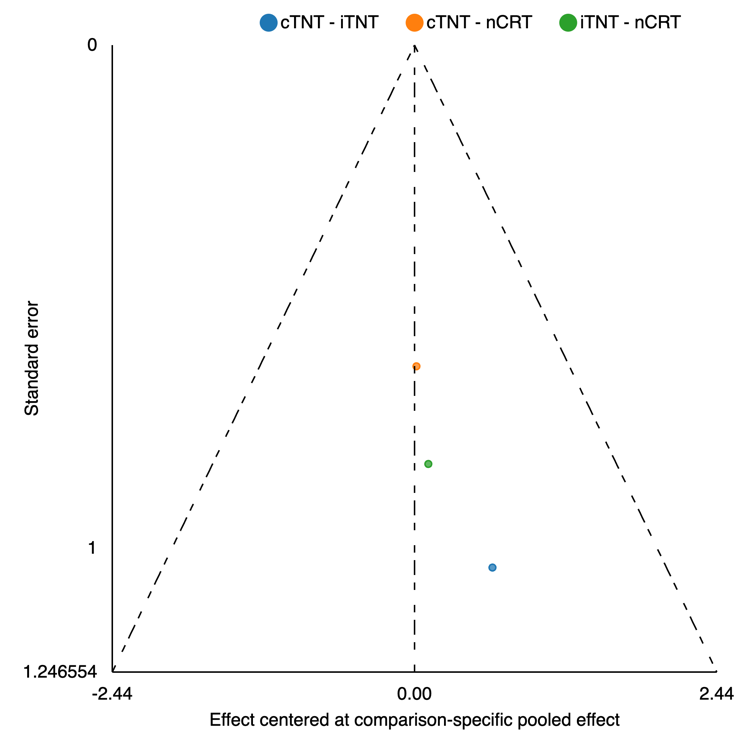 |
| Bowel obstruction | Pancreatitis |
| 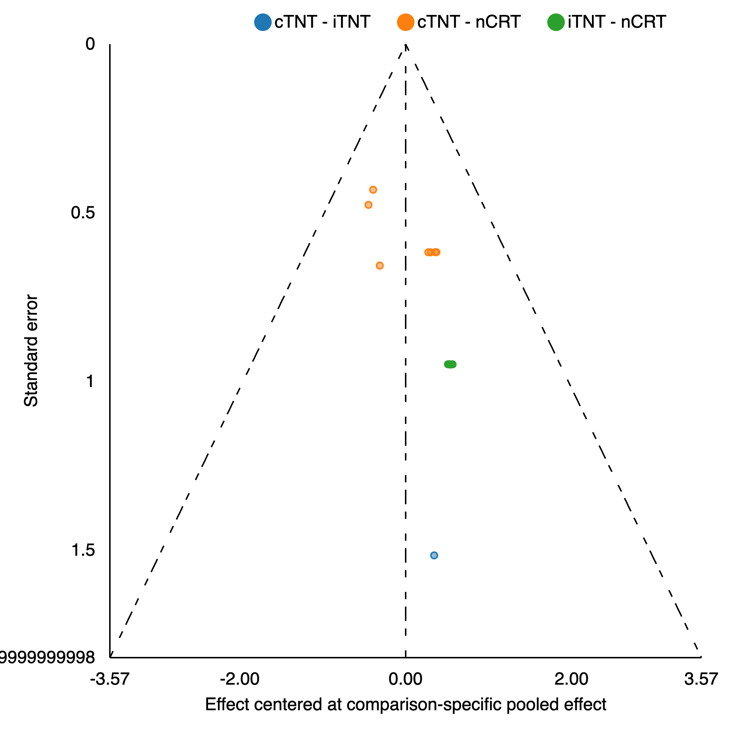 | 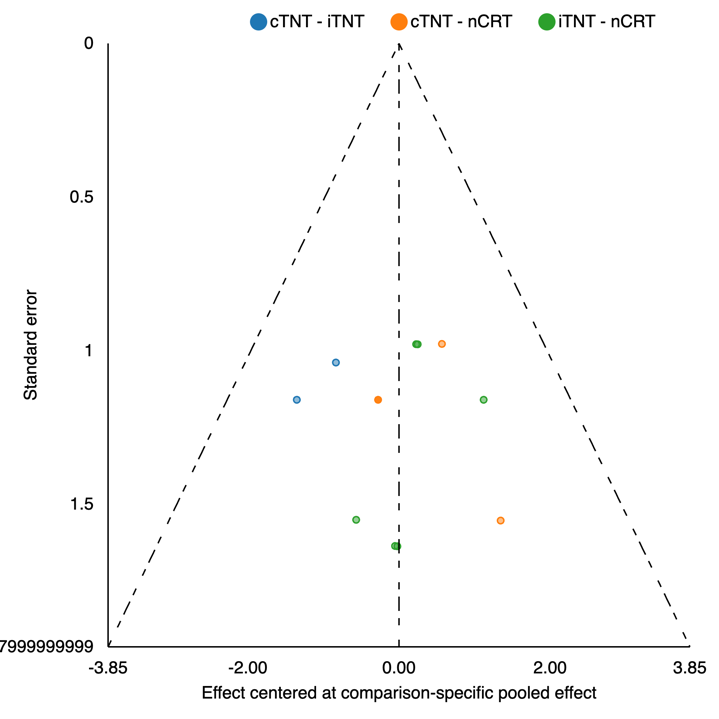 |
| Proctitis | Rectal bleeding |
| 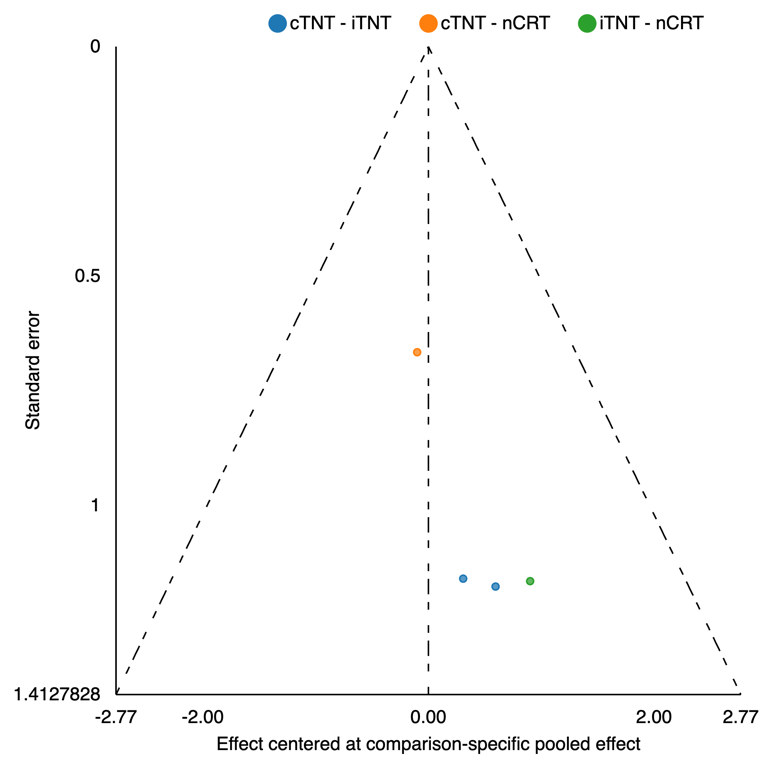 | 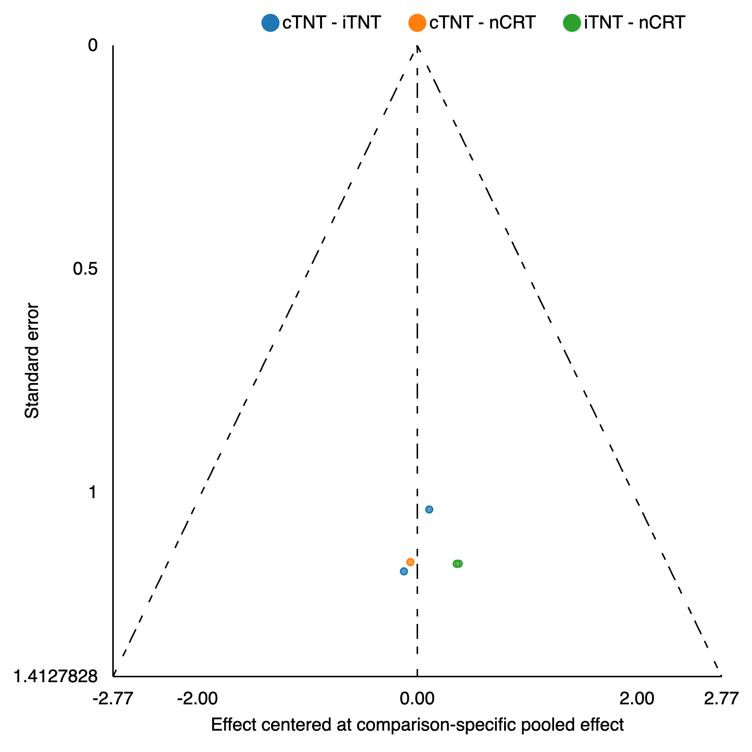 |
| Dizziness | Dysarthria |
| 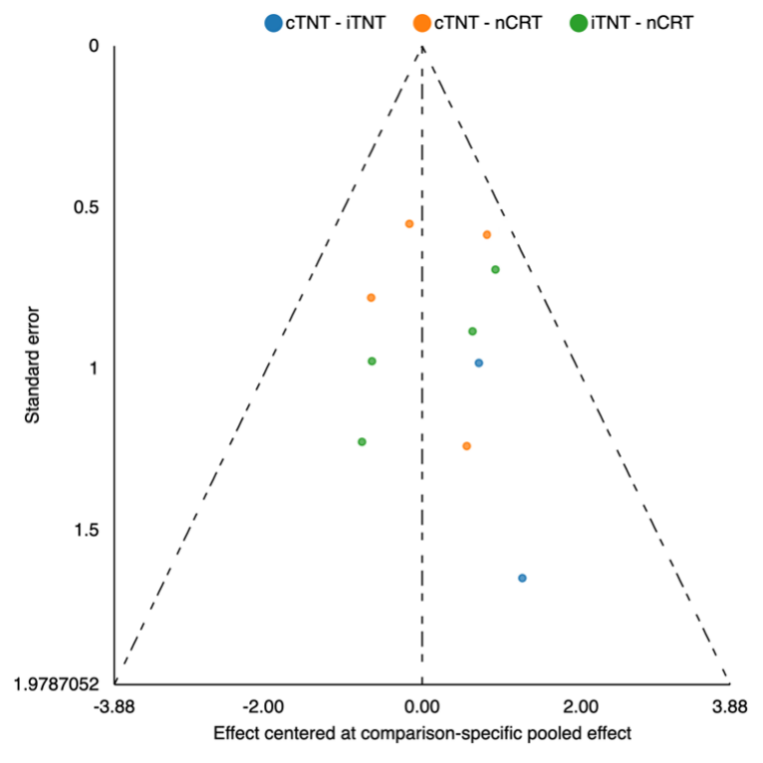 | 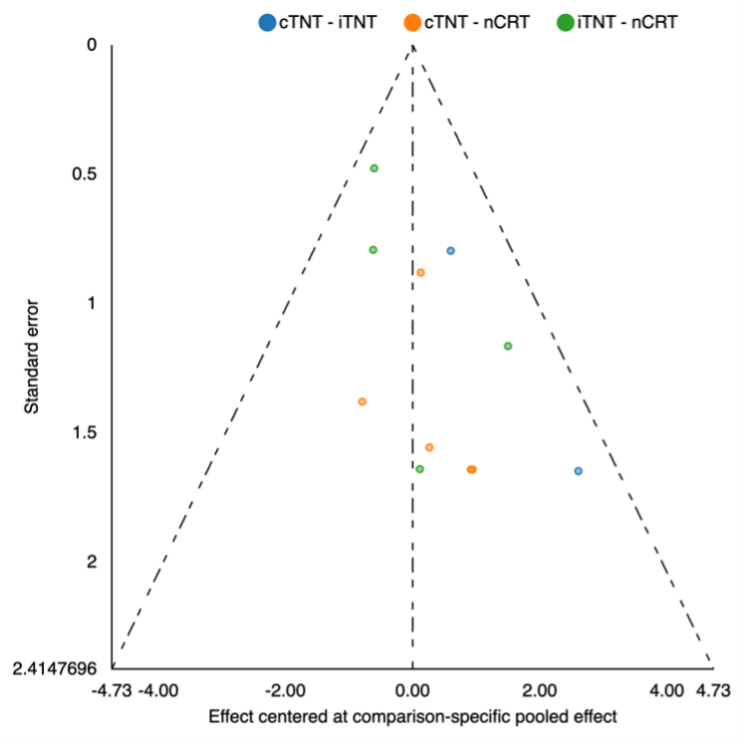 |
| Hand-foot syndrome | Neuropathy |
| 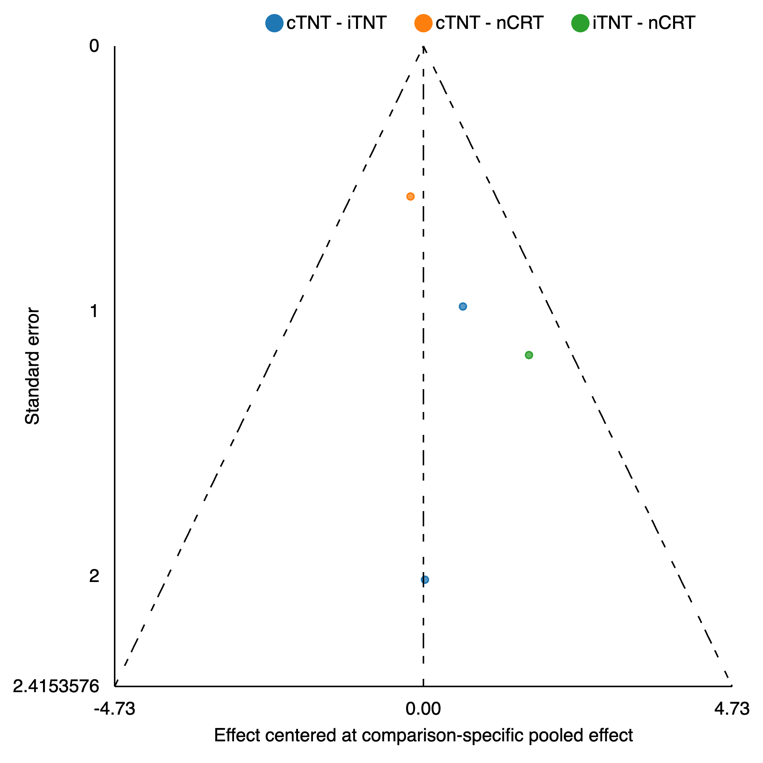 | 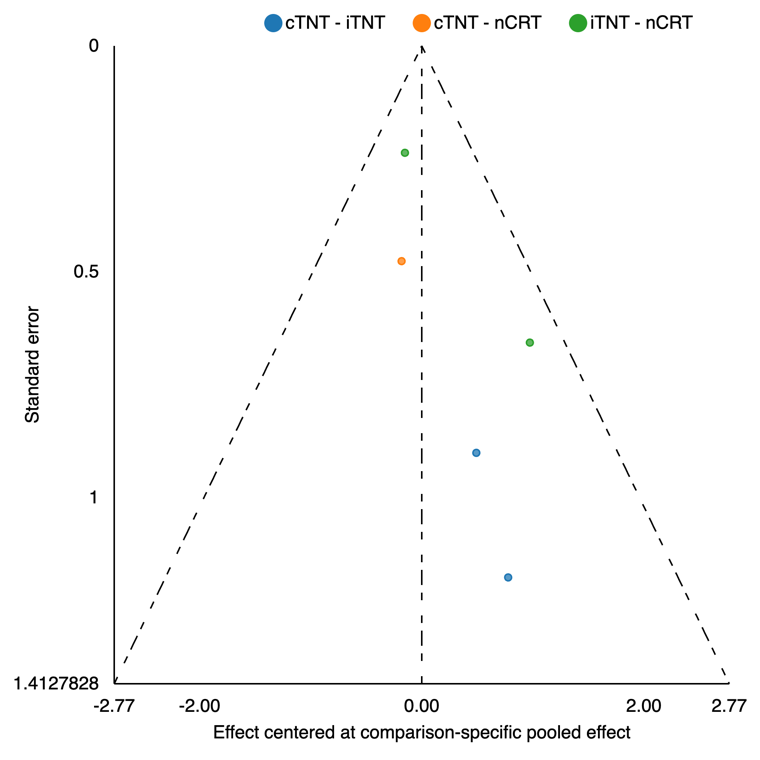 |
| Syncope | Musculoskeletal weakness |
| 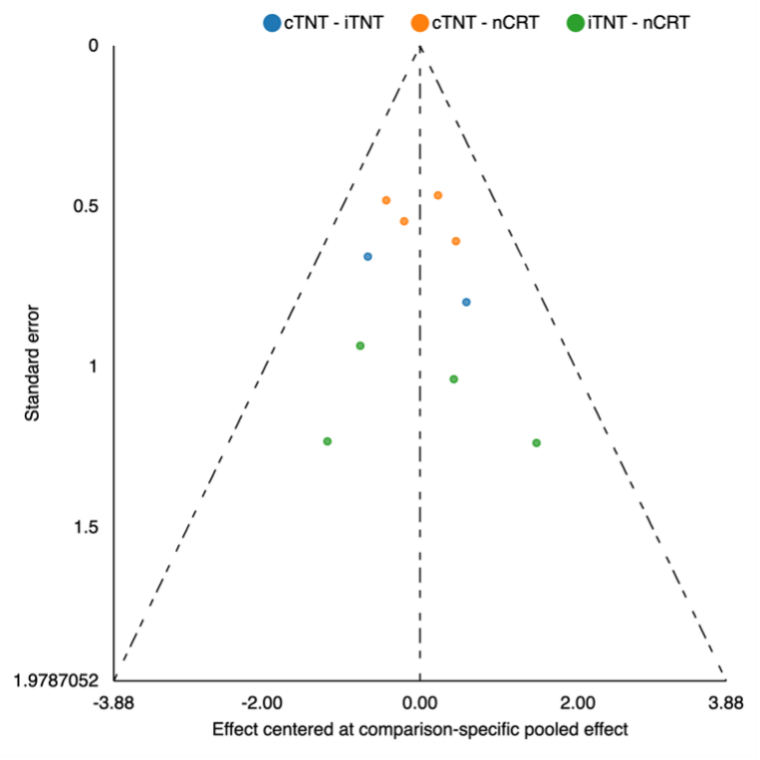 | 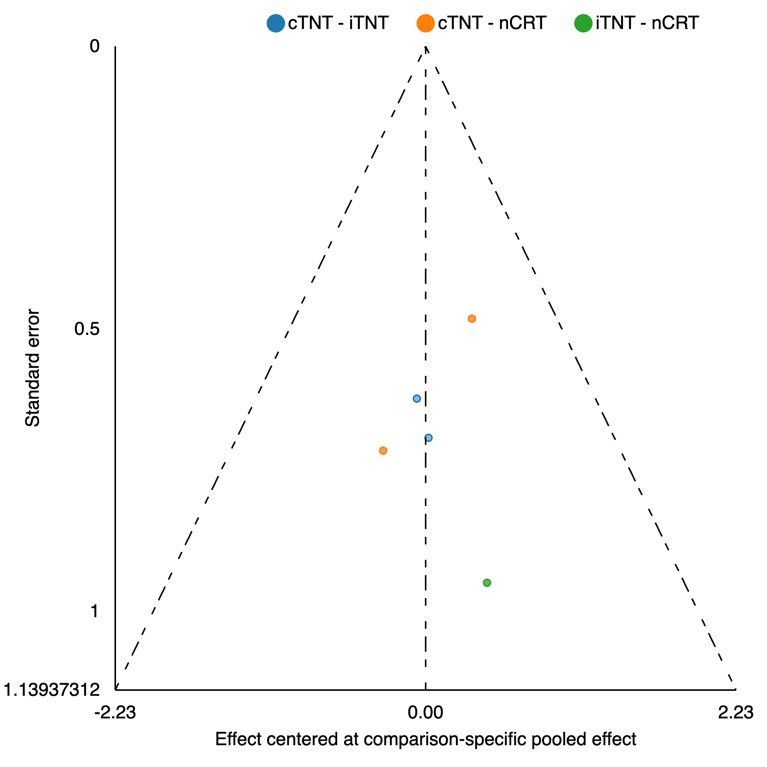 |
| Arrythmias | Venous thromboembolism |
| 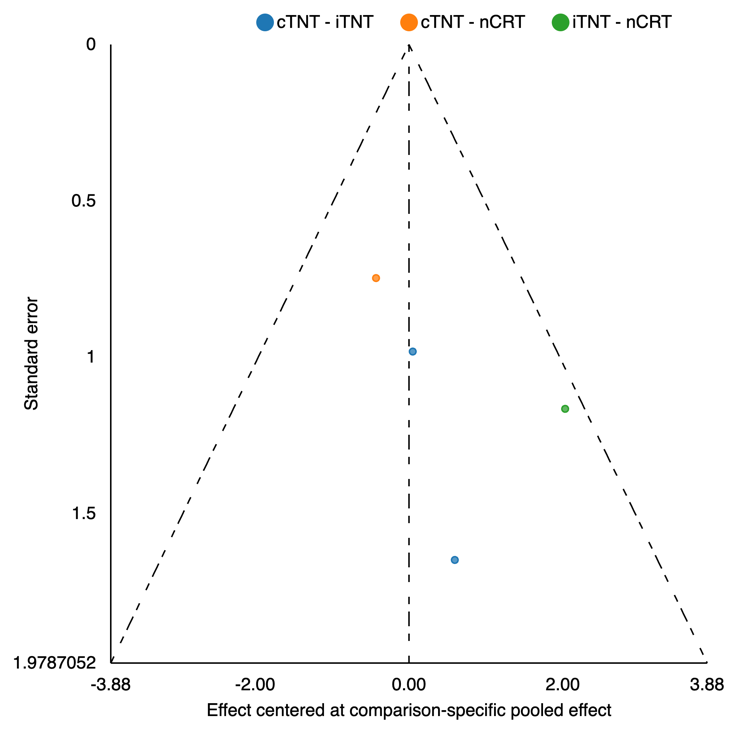 | 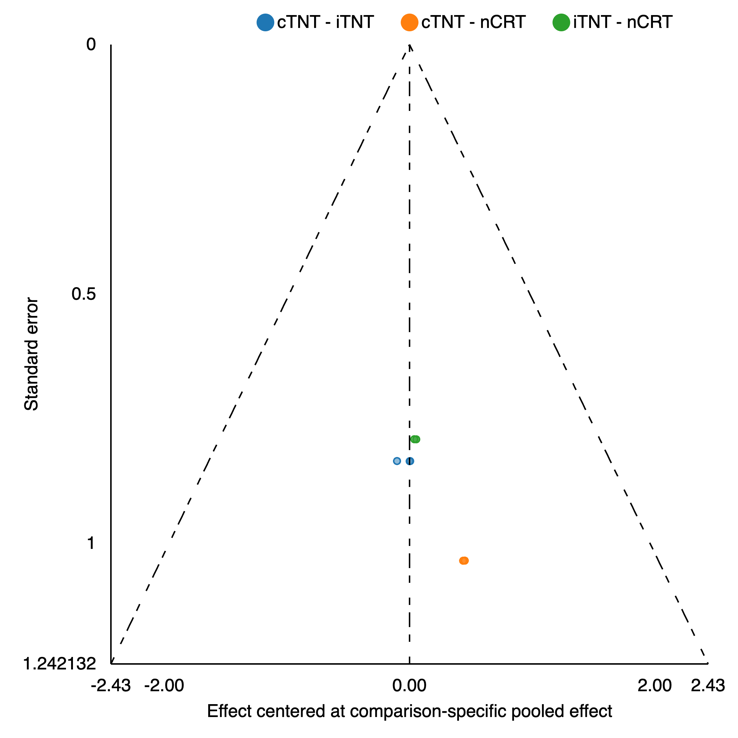 |
| Pneumonia | Urinary tract infection |
| 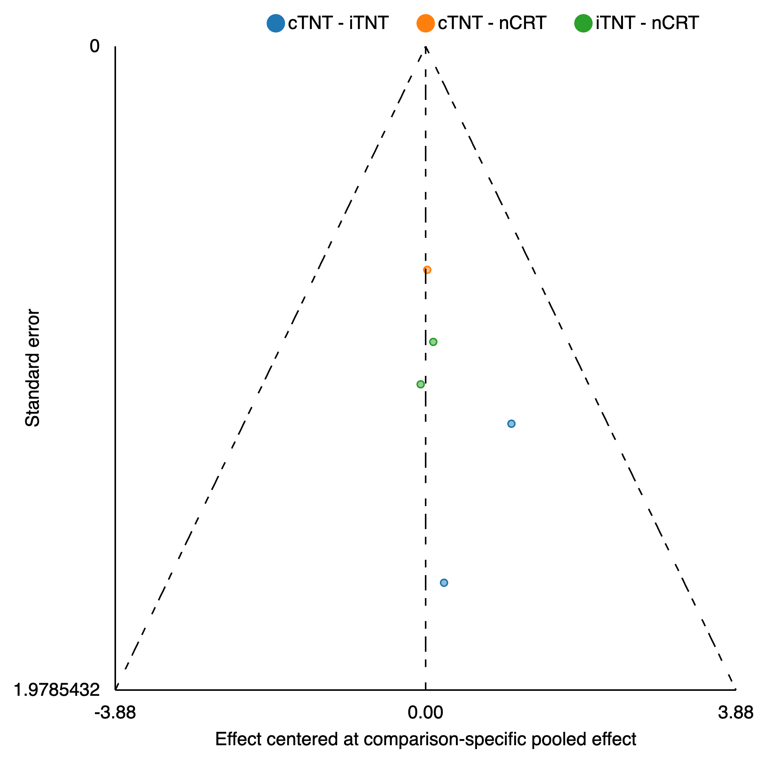 | 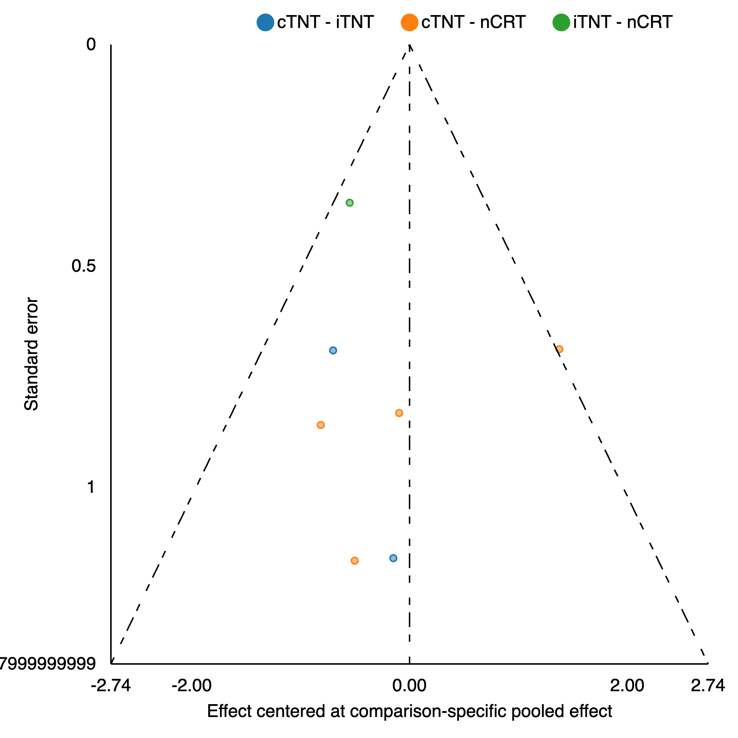 |
| Sepsis | Radiation dermatitis |
| 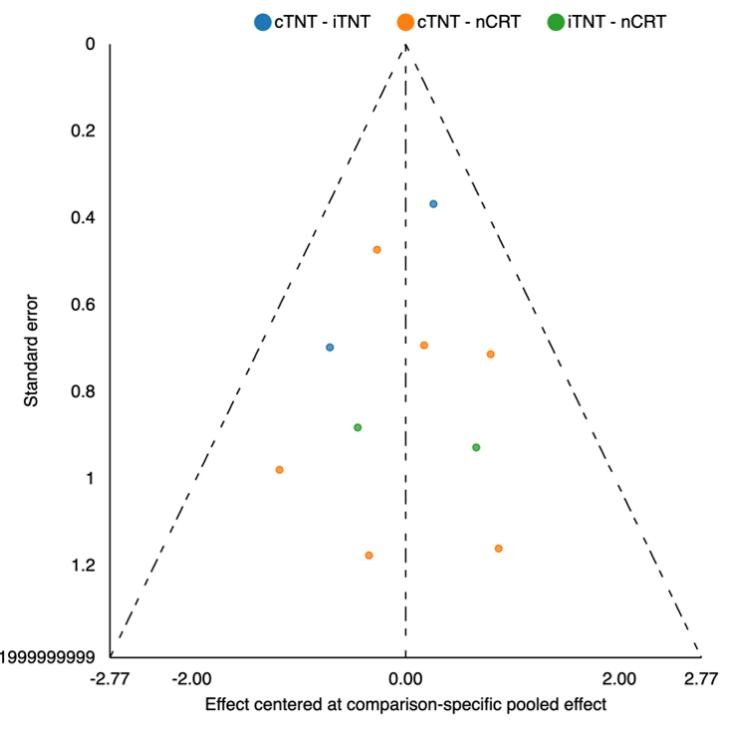 | 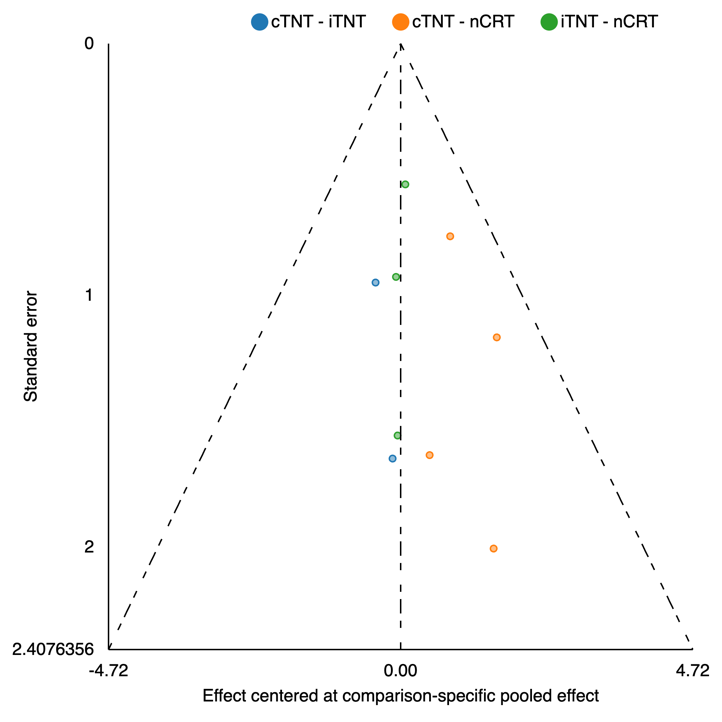 |
| Renal and electrolyte imbalance | Febrile neutropaenia |
| 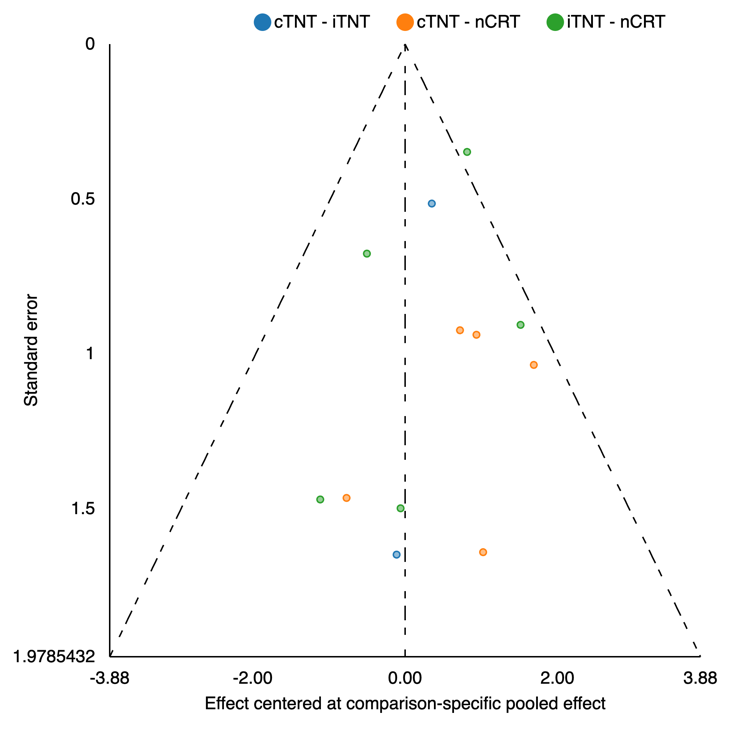 | 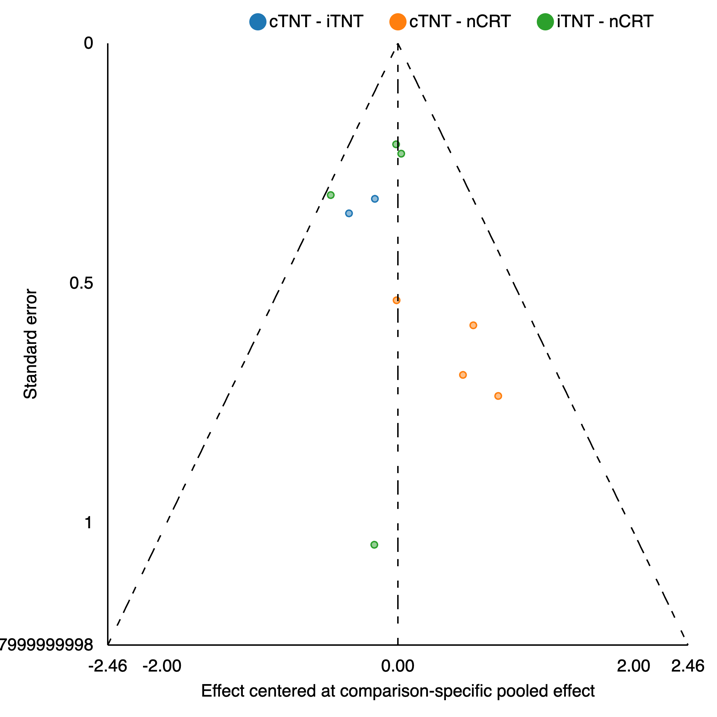 |
| Neutropaenia | Lymphopenia |
| 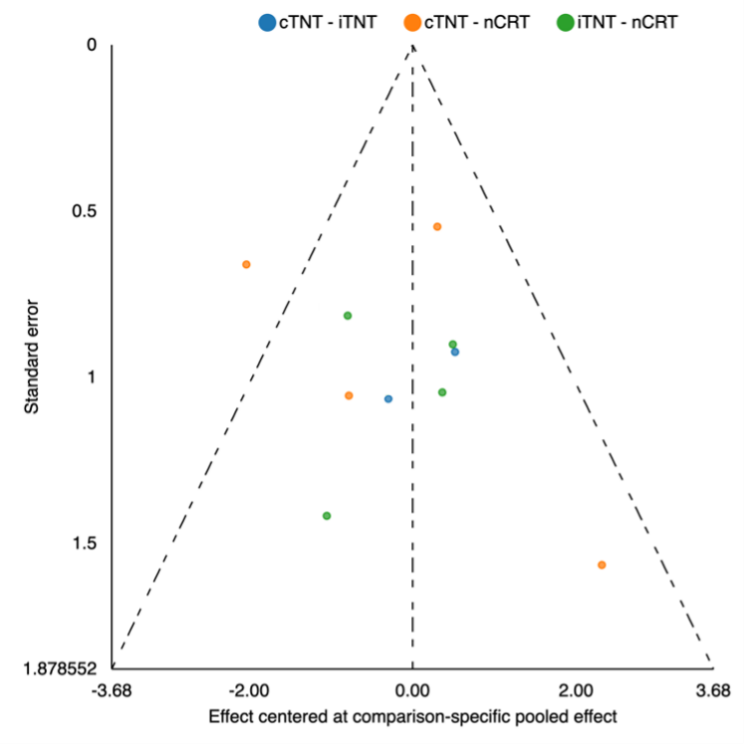 | 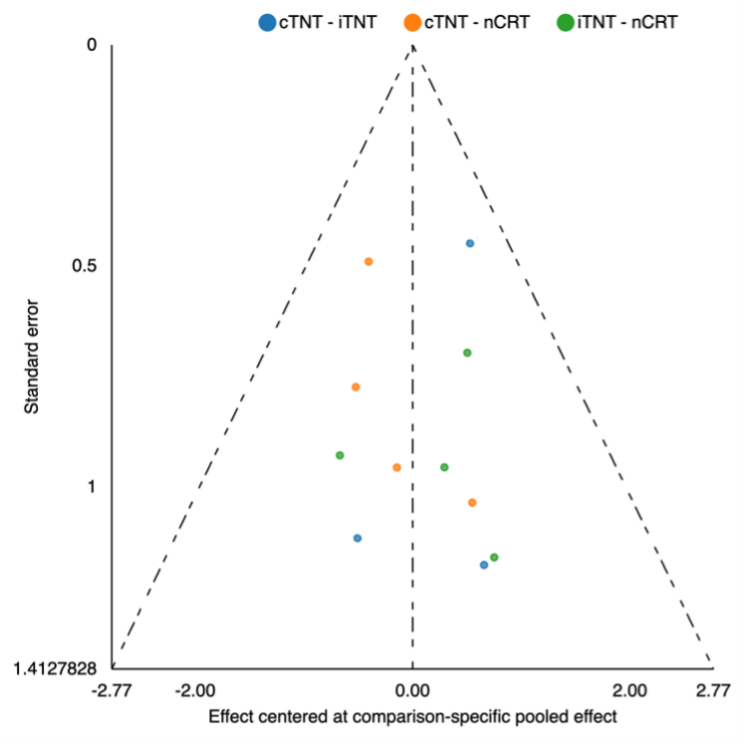 |
| Thrombocytopenia | Anaemia |
| 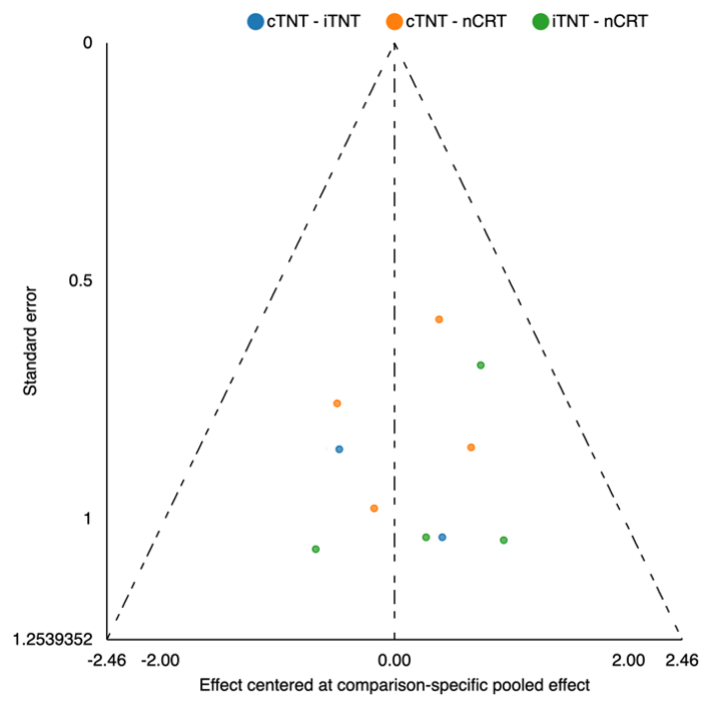 |  |
| Anaphylaxis |  |
| **Compliance outcomes** | |
| 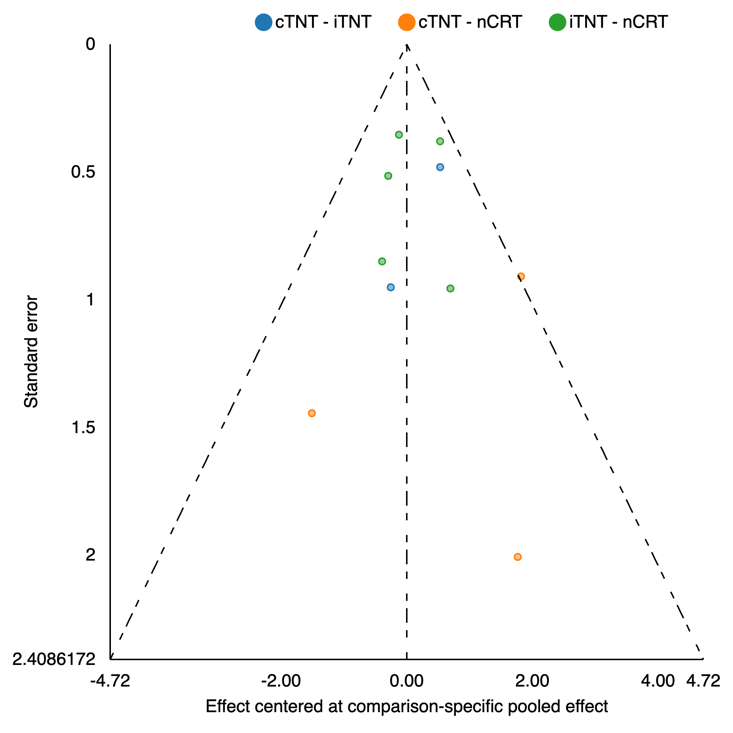 | 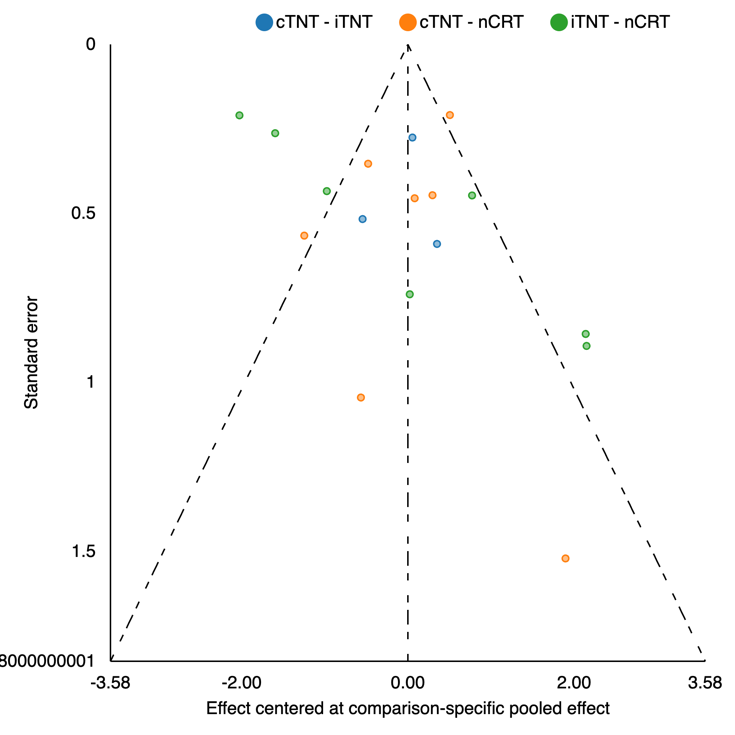 |
| Compliance with radiotherapy (≥4500Gy) | Compliance with chemotherapy (overall) >90% |
| 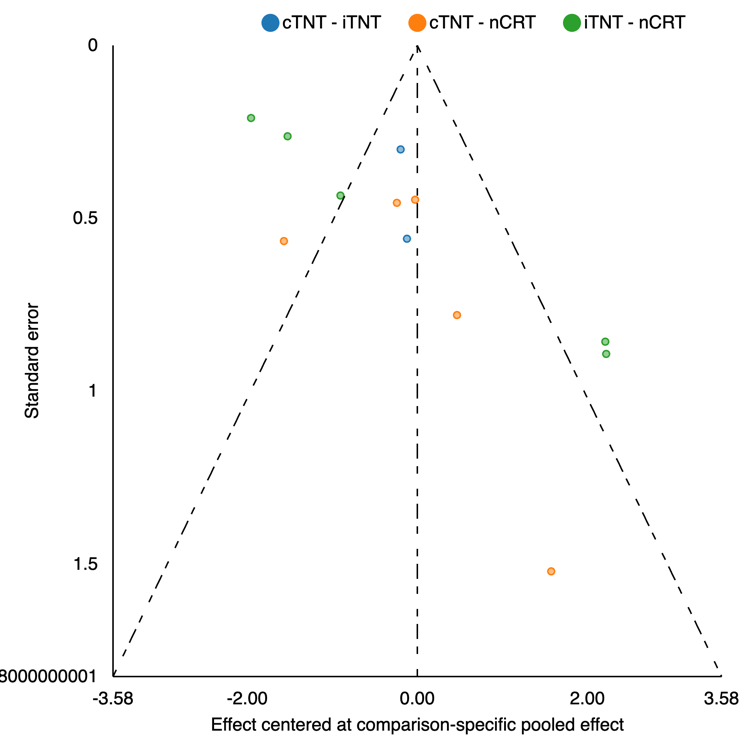 |  |
| Compliance with FOLFOX chemotherapy (>90%) | Compliance with CAPOX chemotherapy (>90%) |
| **Postoperative outcomes** | |
|  |  |
| Anastomotic leak | High stoma output/diarrhoea |
|  |  |
| Postoperative bowel obstruction | Organ/space surgical site infection |
|  |  |
| Superficial incisional surgical site infection | Urinary tract infection |
|  |  |
| Overall Clavien-Dindo Grade 3 and above postoperative complications |  |
